# New chemical probes targeting cholesterylation of Sonic Hedgehog in human cells and zebrafish†
†Electronic supplementary information (ESI) available: Tables S1–S2; Fig. S1–S9, full experimental details and procedures, ^1^H/^13^C NMR spectral data of compounds **1–6**. See DOI: 10.1039/c4sc01600a



**DOI:** 10.1039/c4sc01600a

**Published:** 2014-08-15

**Authors:** Paulina Ciepla, Antonios D. Konitsiotis, Remigiusz A. Serwa, Naoko Masumoto, Wai P. Leong, Margaret J. Dallman, Anthony I. Magee, Edward W. Tate

**Affiliations:** a Department of Chemistry , Imperial College London , Exhibition Road , London SW7 2AZ , UK . Email: e.tate@imperial.ac.uk; b Institute of Chemical Biology , Imperial College London , Exhibition Road , London SW7 2AZ , UK . Email: t.magee@imperial.ac.uk; c National Heart and Lung Institute , Imperial College London , Exhibition Road , London SW7 2AZ , UK; d Department of Life Sciences , Imperial College London , Exhibition Road , London SW7 2AZ , UK

## Abstract

Sonic Hedgehog protein (Shh) is a morphogen molecule important in embryonic development and in the progression of many cancer types in which it is aberrantly overexpressed. Fully mature Shh requires attachment of cholesterol and palmitic acid to its C- and N-termini, respectively. The study of lipidated Shh has been challenging due to the limited array of tools available, and the roles of these posttranslational modifications are poorly understood. Herein, we describe the development and validation of optimised alkynyl sterol probes that efficiently tag Shh cholesterylation and enable its visualisation and analysis through bioorthogonal ligation to reporters. An optimised probe was shown to be an excellent cholesterol biomimetic in the context of Shh, enabling appropriate release of tagged Shh from signalling cells, formation of multimeric transport complexes and signalling. We have used this probe to determine the size of transport complexes of lipidated Shh in culture medium and expression levels of endogenous lipidated Shh in pancreatic ductal adenocarcinoma cell lines through quantitative chemical proteomics, as well as direct visualisation of the probe by fluorescence microscopy and detection of cholesterylated Hedgehog protein in developing zebrafish embryos. These sterol probes provide a set of novel and well-validated tools that can be used to investigate the role of lipidation on activity of Shh, and potentially other members of the Hedgehog protein family.

## Abstract 2

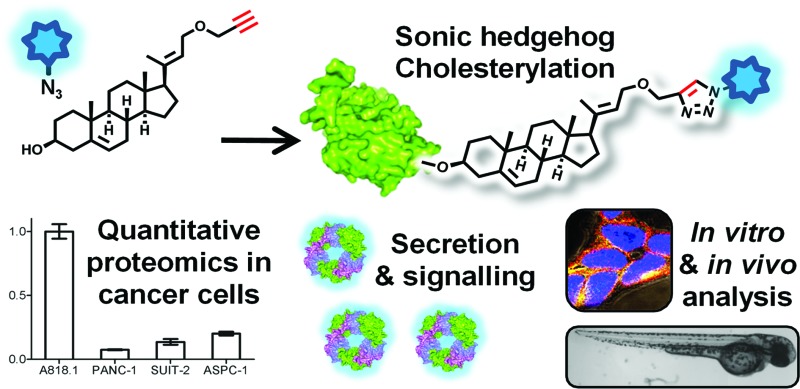
Alkynyl-cholesterol probes tag and track Hedgehog protein, illuminating the role of protein cholesterylation in secretion, transport complex formation and signalling, and enabling quantitative proteomic analysis, imaging, and detection of cholesterylation in developing zebrafish.

## Introduction

Sonic Hedgehog protein (Shh) is a vertebrate morphogen, mitogen and differentiation factor belonging to the Hedgehog protein (Hh) family. Shh plays a major physiological role in embryonic development where it is critical for appropriate growth of limbs, skin, neuronal progenitor cells, inner ear, eye, taste buds and hair follicles.^1^ The role of Shh is significantly diminished in postnatal and adult organisms, although it is important in the maintenance of stem cells, and for tissue repair and regeneration.^2^ However, Shh expression is regained in many types of cancer, and is widely recognised as a driver of carcinogenesis.^3^

**Fig. 1:**
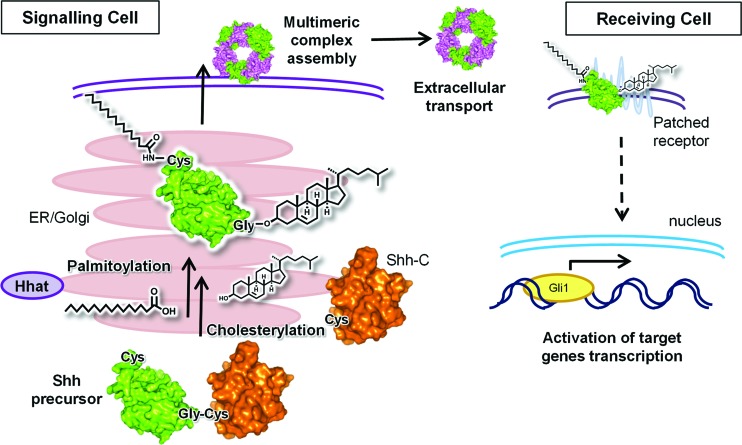
Signalling pathway of ShhN. Processing and maturation of Shh precursor in signalling cell, assembly and transport of multimeric complexes of ShhN in the extracellular space, activation of the ShhN receiving cell through binding of ShhN to its receptor Patched; purple structures represent as yet poorly understood components of the ShhN multimeric complexes.

A fully active Shh signalling molecule is produced through posttranslational processing of a precursor (46 kDa) containing two domains: the N-terminal signalling domain (ShhN) and the C-terminal catalytic domain (ShhC).^4^ Posttranslational modifications of the Shh precursor include removal of the signal peptide to reveal an N-terminal cysteine^5^ followed by N-terminal attachment of palmitate (C16:0)^6^ catalysed by Hedgehog acyltransferase (Hhat),^7,8^ and attachment of cholesterol *via* an *O*-ester linkage to the C-terminus of ShhN. This latter modification is mediated by ShhC,^9^ which shows similarity to intein domains and catalyses its own cleavage to form *N*-palmitoylated/*O*-cholesterylated ShhN (19 kDa)^10^ (Fig. 1).

Lack of one or both lipid modifications results in diminished signalling activity of the protein.^11^ As a result, many mutations of Shh found in genetic diseases, such as holoprosencephaly, are associated with defects in catalysis of processing,^12,13^ suggesting that cholesterol processing of the Shh precursor is essential for the activation of appropriate Shh signalling pathways. The same was found for ShhN palmitoylation, where mutations in ShhN palmitoylation sites or Hhat phenocopy mammalian Shh or *Drosophila* Hh null mutants.^7,14^ Mature ShhN is released from signalling cells through the formation of multimeric complexes of ShhN, which is hypothesised to allow efficient transport and wider signal distribution than hydrophobic ShhN monomers.^15^ At the receiving cell ShhN binds to a receptor, Patched (Ptc),^16^ which activates a complex cellular signalling cascade. The final step of this process consists in activation of Gli transcription factors, responsible for the activation of Shh target gene transcription (Fig. 1).

Although the ShhN signalling pathway has been extensively studied, many questions surrounding the role of ShhN cholesterylation remain unanswered. Contradictory reports have considered the mechanism^6,9,10^ and the cellular site of cholesterylation^17^ and its involvement in ShhN release from signalling cells,^18–20^ assembly of secreted molecules into higher molecular weight complexes^14,21^ and its influence on signalling.^17,22^ However, a major barrier for clear understanding of the role of Shh cholesterylation has been lack of appropriate tools to study the modification in cells. To date, a large majority of studies have relied on evidence from non-native systems, in which genetic mutation of Shh to delete lipid modification sites is followed by overexpression of the mutated protein in cells or model organisms.^12–14,18–21,23–26^ This approach is disruptive to the system under study, only considers the consequences of loss of cholesterylation rather than studying the modification itself *in situ*, and is not readily portable between different systems. A less common approach uses radioactive isotope-labelled cholesterol^9,17,27,28^ incorporated metabolically into ShhN. This tool also suffers from limitations such as long time required for the detection of a signal, use of hazardous material, and low sensitivity. It also requires large amounts of sample for analysis, and cannot provide information in a single cell format. Consequently, these methods fail to answer some key questions about Shh cholesterylation, including: is cholesterylated ShhN released from signalling cells, or is cholesterol lost during the release process?; and if released, is cholesterylated ShhN present in the high molecular weight ShhN extracellular complexes important for signalling?

Recently, however, a new approach to target cholesterylation^22^ and palmitoylation^22,29^ of ShhN has been developed that uses nonradioactive chemical tags metabolically incorporated into ShhN in living cells, and detected by bioorthogonal ligation (*e.g.* Cu(i)-catalysed [3 + 2] azide–alkyne cycloaddition, CuAAC^30^) to a visualisation reagent. Metabolic substrate analogues used in this technique must have high specificity, high signal-to-noise ratio, low cellular toxicity and a low working concentration; furthermore, they should allow labelling in various systems and conditions without interrupting the biological functions of the labelled proteins. In the case of Shh cholesterylation, in order to address the outstanding questions noted above such a tool should allow detection of modified Shh endogenously both in cells and after lysis, and following the secretion of the protein from the signalling cell, without disrupting signalling. We previously reported the first azide-tagged cholesterol probe (AzChol)^22^ that can be used to label ShhN in cells; however, AzChol has several significant limitations as a tool: it is incorporated at <15% efficiency even in highly overexpressing systems at probe concentrations (50–100 μM) that are too toxic for studies of longer-term signalling events or for application *in vivo*, and it also suffers from low signal-to-background ratio and problematic non-specific labelling. We therefore sought to design new cholesterylation probes that allow rapid and quantitative biochemical analysis of cholesterylated ShhN produced by mammalian cells. We show here that an optimised probe enables efficient and highly specific sterylated ShhN (ShhN*) labelling with no detectable toxicity.

We present the first demonstration of tracking ShhN* synthesis through release into cell culture medium and subsequent signalling to receiving cells, and single cell imaging of probe using fluorescence microscopy. Furthermore, we apply this probe to the questions noted above, including the release and packaging of ShhN* signalling complexes, and direct quantification of endogenous ShhN* levels in cancer cell lines through quantitative chemical proteomics. We also demonstrate for the first time that lipid probes can efficiently tag sterylated Hedgehog proteins *in vivo* in zebrafish embryos (*Danio rerio*), a widely used vertebrate model of development.

## Results and discussion

### New cholesterylation probes to target Sonic Hedgehog

**Fig. 2:**
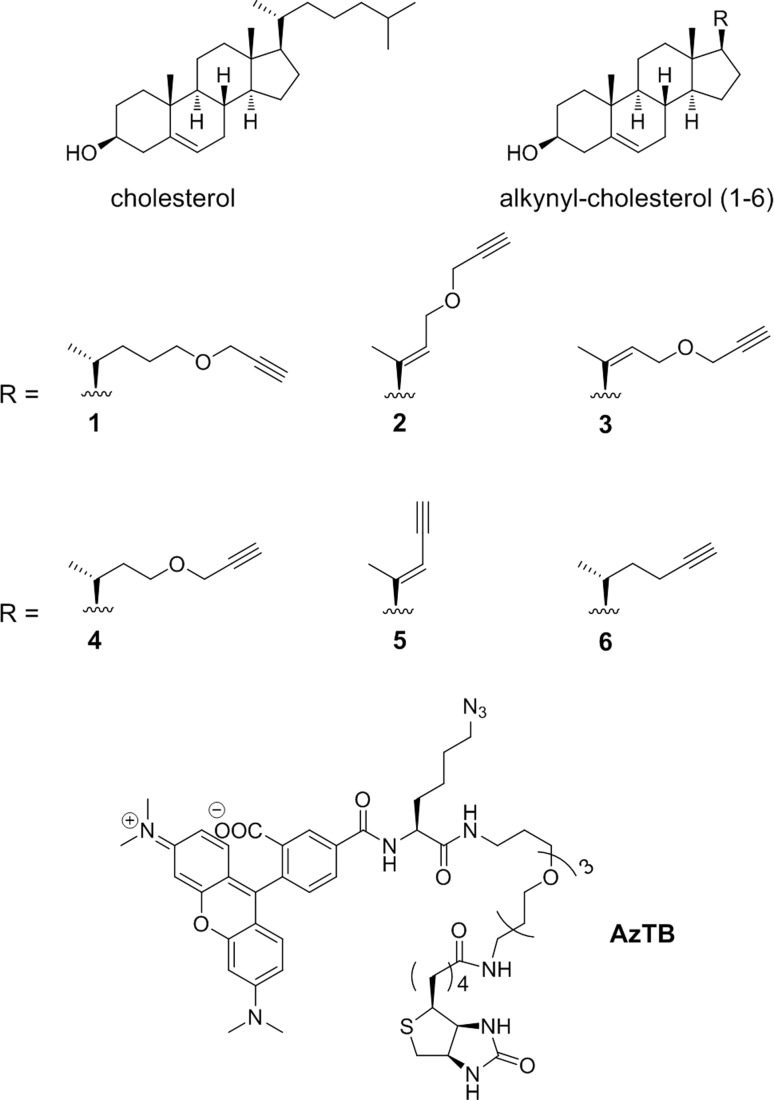
Cholesterol, alkynyl-cholesterol analogues (**1–6**) and capture reagent **AzTB**.

**Fig. 3:**
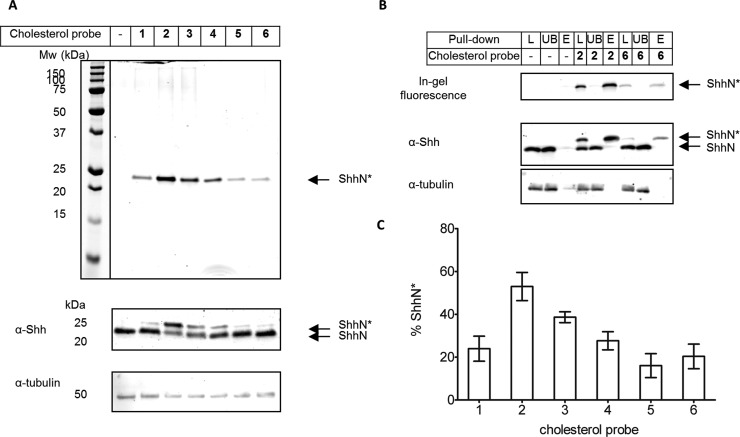
Incorporation of alkynyl-cholesterol analogues into ShhN in HEK293a Shh+ cells. Cells metabolically labelled with 5 μM **1–6**, or DMSO, for 16 hours, lysed and ligated by CuAAC to **AzTB**. (A) Proteins were separated by SDS-PAGE and analysed by in-gel fluorescence and immunoblot with antibodies against Shh or tubulin (loading control). ShhN*: ShhN tagged with alkynyl-cholesterol analogues; ShhN: processed untagged Shh protein. (B) Affinity protein purification using streptavidin-coated magnetic beads. In-gel fluorescence and immunoblotting image with antibodies against Shh or tubulin (loading control). [L] **AzTB** ligated sample, pre-purification; [UB] unbound proteins after purification; [E] proteins eluted from beads. (C) Analysis of total amount of ShhN* normalised to total processed Shh (ShhN* + ShhN), quantified by chemiluminescence following immunoblot with α-Shh antibody (*n* = 5, error bars ± SD). Significance (*p* value) measured by one-way ANOVA (SI Table 1†).

Our original design for AzChol benefitted from straightforward synthetic access to this class of probe molecules; however, we and others have observed that azide-tagged probes can often result in amplified background labelling in cells relative to comparable alkyne-tagged counterparts.^31,32^ We therefore elected to synthesise novel cholesterol analogues (**1–6**) bearing terminal alkyne functionalities (Fig. 2). Other akynyl sterols have been previously used in conjunction with CuAAC to identify cholesterol-interacting proteins,^33,34^ or cholesterol metabolism and cellular localisation;^35^ however, none of these reports has described covalent modification of proteins by alkynyl sterols. Terminal alkyne modifications were employed in place of the C20–27 aliphatic chain of cholesterol since modification at this position can be tolerated in ShhC autoprocessing.^36^ The analogues **1–4** also contained ether functionality, offering straightforward synthesis and potentially improving solubility for cell-based assays. To explore the potential of each compound to act as a substrate for ShhN cholesterylation, HEK 293a cells stably transfected to overexpress Shh (HEK293a Shh+)^37^ were treated with 5 μM of each compound for 16 hours to allow metabolic probe incorporation. The cells were then lysed and subjected to CuAAC ligation to azide-containing trifunctional capture reagent azido-TAMRA-PEG-Biotin (**AzTB**).^22^ Proteins were separated using SDS-PAGE gel and visualised by in-gel fluorescence and anti-Shh immunoblotting (Fig. 3A). All alkynyl-cholesterol analogues labelled specifically a single band at ∼23 kDa, which was identified as probe-modified ShhN (ShhN*) by immunoblot analysis. This result was in contrast to AzChol or [^3^H]cholesterol which have been suggested to be incorporated in other proteins,^9,22^ suggesting that probes **1–6** show superior selectivity towards Shh protein in these cell based assays in comparison with previously reported probes. Anti-Shh immunoblotting further revealed a shift to a higher apparent molecular weight upon CuAAC ligation to **AzTB** (Fig. 3A), further confirmed by affinity purification of biotinylated proteins with streptavidin-coated beads (Fig. 3B); this shift is readily accounted for by the increase in ShhN molecular weight following ligation to **AzTB**.

The intensity of the ShhN* band varied between compounds, highlighting differences in cellular incorporation into ShhN, and the band shift between ShhN* and ShhN allowed quantification of the extent of ShhN labelling (Fig. 3C), a parameter that is extremely difficult to determine for [^3^H]cholesterol. Compound **2** was found to have the highest incorporation (∼53% of total ShhN) under these conditions. Compounds containing an ether moiety were also incorporated more efficiently than those with hydrocarbon chains and extension or shortening of the aliphatic chain by one methylene unit did not have a significant effect on metabolic labelling (**1** *vs.* **4**). A significant increase in labelling was observed in allylic ether-linked probes (**2**, **3** and **4**), and a significant discrimination of *cis*- *versus trans*-isomers (**2** and **3**) was observed. These data suggest that ShhC autoprocessing is tolerant to small variations in probe structure, and can accommodate various alkyne-tagged cholesterol analogues.

### Probe 2 is a biomimetic substrate for monitoring Shh cholesterylation

**Fig. 4:**
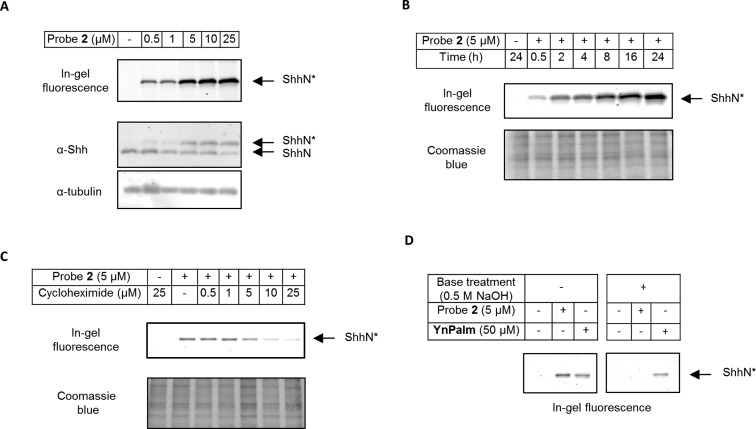
Validation of **2** as an optimised ShhN cholesterylation probe. (A) Concentration-dependent incorporation of **2** during Shh processing. HEK293a Shh+ cells were treated with the specified concentration of **2** for 16 hours, lysed and ligated *via* CuAAC to **AzTB**. Proteins were separated by SDS-PAGE and visualised by in-gel fluorescence or immunoblot against Shh or tubulin (loading control). ShhN*: ShhN tagged with alkynyl-cholesterol analogues; ShhN: untagged, processed Shh protein. (B) Progressive incorporation of **2** into ShhN over time in cells. HEK293a Shh+ cells were treated with 5 μM **2** for the period indicated, lysed and labelled with **AzTB**. Proteins were separated by SDS-PAGE and imaged by in-gel fluorescence (Coomassie blue, loading control). (C) Incorporation of **2** in ShhN is dependent on *de novo* protein synthesis. HEK293a Shh+ cells were treated with varying concentrations of cycloheximide for 1 hour prior to treatment with 5 μM **2** and cycloheximide for 16 hours, then lysed and labelled with **AzTB** capture reagent. Proteins were separated by SDS-PAGE and imaged by in-gel fluorescence (Coomassie blue, loading control). (D) **2** is linked to ShhN through a base-labile bond, consistent with a C-terminal *O*-ester linkage. HEK293a Shh+ cells treated for 16 hours with 5 μM of **2**, or 50 μM of alkynyl-palmitate analogue **YnPalm** (pentadec-14-ynoic acid), were lysed, Shh was immunoprecipitated using α-Shh antibody and Pureproteome protein G magnetic beads and labelled with **AzTB** on-resin. Proteins were separated by SDS-PAGE and imaged by in-gel fluorescence before and after soaking the gel in 0.5 M NaOH(aq) for 2 hours at room temperature. ShhN* metabolically labelled with **YnPalm**, a palmitate analogue previously shown to label Shh palmitoylation^22^ through a N-terminal amide bond, is a negative control for base hydrolysis.

Further studies were focused on probe **2** in view of its superior incorporation into ShhN. Initial experiments examined concentration- (Fig. 4A) and time- (Fig. 4B and Fig. S1A†) dependence of metabolic labelling. Labelling increased with concentration of **2** up to 25 μM, above which solubility in cell culture medium became limiting. 5 μM **2** appeared to offer a balanced level of fluorescence and degree of incorporation (ShhN*/total ShhN), and was used for further studies. Notably, as little as 0.5 μM of **2** was sufficient to achieve readily-detectable labelling of ShhN*, a > 100-fold increase in sensitivity over the previously reported AzChol probe.^22^ Extending incubation time also increased probe incorporation up to 24 hours. Detectable incorporation of **2** was observed after only 30 minutes, suggesting rapid cellular uptake of the probe and transport to the Shh precursor processing site. However, steady state incorporation was established between 8 and 16 hours, suggesting that the rate of Shh precursor synthesis may be a limiting factor in labelling ShhN*. To test this theory, cellular protein synthesis was inhibited by varying concentrations of cycloheximide (0 to 25 μM) for 1 hour, followed by incubation with 5 μM **2** and cycloheximide for 16 hours (Fig. 4C and S1B†). Degree of incorporation of the probe decreased progressively with increasing concentration of cycloheximide, suggesting that **2** is incorporated only into newly translated Shh protein. These two observations suggest that ShhN* posttranslational modification occurs rapidly following protein synthesis, in agreement with previous studies describing autocatalysis and attachment of cholesterol as an early process in the ShhN secretion pathway, occurring predominately in endoplasmic reticulum (ER) or Golgi apparatus of ShhN producing cells.^17^ The known mechanism of Shh processing involves formation of an ester bond between the carboxylic acid of the C-terminal glycine residue of ShhN and the 3β-hydroxyl group of cholesterol.^9,10^ To confirm that this mechanism is conserved for **2**, protein from HEK293a Shh+ cells treated with **2** or **YnPalm** (alkynyl-palmitate analogue pentadec-14-ynoic acid)^22^ were ligated by CuAAC to **AzTB**, and separated on an SDS PAGE gel that was further treated with 0.5 M NaOH(aq) (Fig. 4D).^9^ Base abolished labelling of ShhN* with **2**, consistent with ester hydrolysis, whereas **YnPalm** remained bound to the protein, consistent with base-stable N-terminal amide-linked palmitate. Taken together, these data show that key mechanistic details of incorporation of endogenous cholesterol in ShhN* are conserved for **2**, and that **2** is an excellent biomimetic probe for ShhN cholesterylation.

### ShhN* in morphogenic gradient formation and signalling

**Fig. 5:**
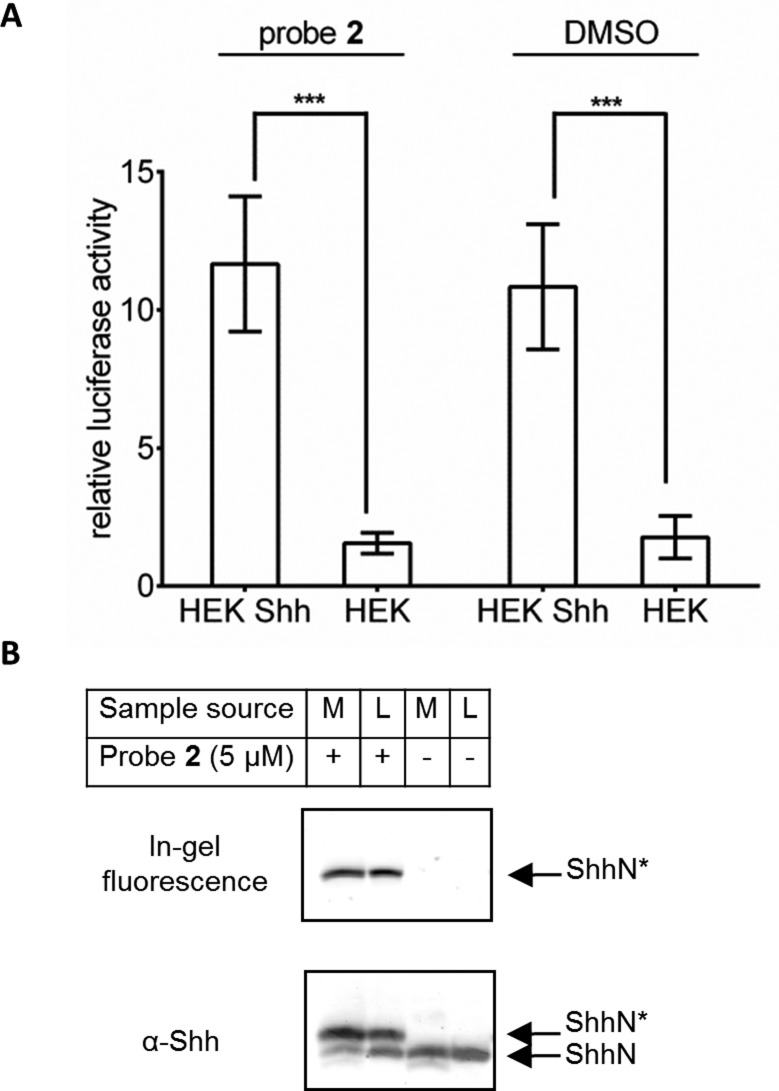
(A) ShhN* metabolically labelled with **2** retains full signalling activity. Medium was harvested from HEK293a Shh+ cells metabolically labelled with **2** or DMSO for 48 hours, and capacity to induce the Hh pathway was measured over 30 hours using NIH-3T3 cells stably transfected with firefly luciferase fused with a Shh pathway Gli promoter, and a Renilla luciferase constitutive reporter (Shh Light2 cells).^38^ Reporter activity (firefly luciferase) was normalised to Renilla luciferase and further normalised to reporter activity elicited by medium from HEK293a cells. Error bars represent ± SD, *p* value measured from two-tailed, unpaired *t* test; (***) < 0.001, *n* = 5 (HEK293a) or *n* = 7 (HEK293a Shh+) independent replicate experiments. (B) ShhN* metabolically labelled with **2** is exported efficiently from cells. Medium was harvested from HEK293a Shh+ cells metabolically labelled with **2** or DMSO for 48 hours, medium [M] and cell lysate [L] were immunoprecipitated with α-Shh antibody and Pureproteome protein G magnetic beads, and labelled with **AzTB** on-resin. Proteins were separated by SDS-PAGE and imaged by in-gel fluorescence or immunoblotting against Shh. ShhN*: ShhN tagged with alkynyl-cholesterol analogues; ShhN: untagged, processed Shh protein.

To further investigate the scope of probe **2** we explored ShhN signalling in the context of labelling Shh cholesterylation. A Hh reporter assay (Fig. 5A) was established whereby HEK 293a Shh+ or untransfected HEK293a (which do not natively express Shh) cells were incubated with **2** for 48 hours, and cell culture media were harvested and added to Shh Light2 reporter cells. Shh Light2 cells express firefly luciferase upon Hh signalling pathway activation, and express Renilla luciferase constitutively as an internal control for signal intensity.^38^ This assay showed no significant difference between Shh signalling pathway activation when HEK293a Shh+ cells were treated with vehicle control (DMSO) or with **2**. To confirm that ShhN* can participate directly in ShhN signal activation, release of ShhN and ShhN* to cell culture media was studied (Fig. 5B). Cells incubated with probe **2** showed similar ratios of ShhN* to total ShhN in both cell lysate and in cell media, showing that ShhN* is released by the signalling cell. The amount of unlabelled ShhN in cells treated with **2** (lower molecular weight band) was substantially reduced relative to control without altering the level of Shh pathway activation, suggesting that ShhN* participates in ShhN pathway activation and retains similar signalling activity to ShhN.

We next sought to demonstrate the potential of probe **2** in enabling new experiments to explore the role and function of ShhN cholesterylation. Movement of ShhN in the extracellular space is thought to be facilitated through the formation of multimeric Shh complexes, and these structures are thought to be the species of ShhN most active in signalling.^14,21^ However, all previous studies of the significance of post-translational lipidation of these complexes have been based on loss of function through genetic mutation and deletion of a specific modification site, or sites,^14,21,26,39^ and thus do not directly address the role of Shh lipidation *in situ*.

**Fig. 6:**
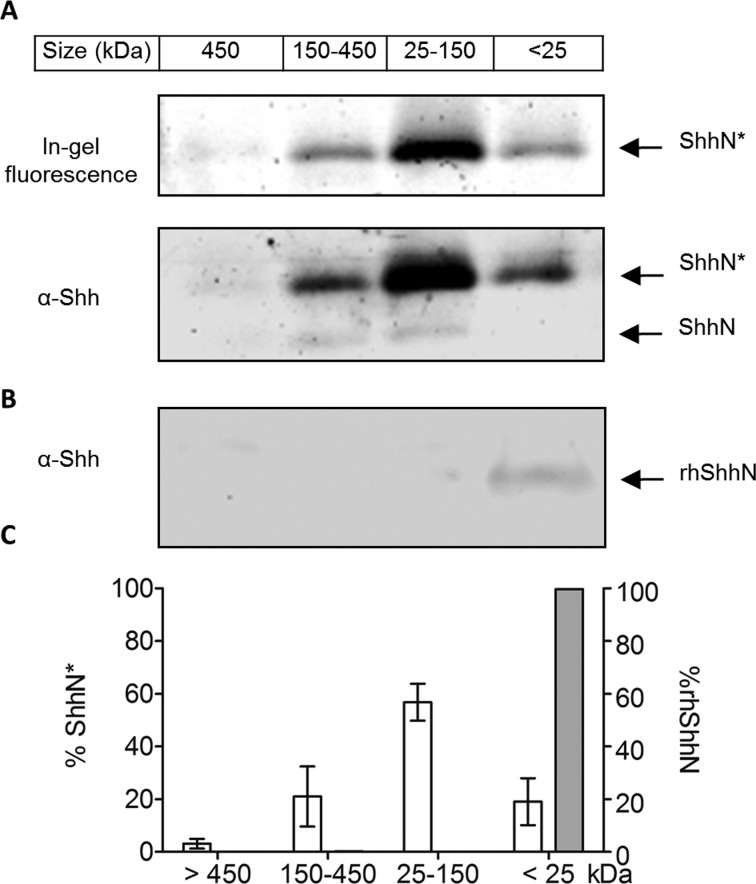
ShhN* metabolically labelled with **2** is efficiently incorporated into multimeric complexes following release from HEK293a Shh+ cells. (A) Cells were metabolically labelled with 5 μM **2** for 48 hours, the culture medium was harvested, concentrated and subjected to size exclusion chromatography (SEC). Fractions were immunoprecipitated and labelled as in Fig. 4. Immunoprecipitated proteins were separated by SDS-PAGE. In-gel fluorescence image and immunoblot against Shh. ShhN*, ShhN tagged with alkynyl-cholesterol analogues; ShhN, untagged, processed Shh protein. (B) 1 μg of rhShhN protein was diluted and incubated for 48 hours in cell culture medium before being subjected to SEC separation. Fractions were immunoprecipitated and proteins were separated by SDS-PAGE. Immunoblot against Shh shown. (C) Percentage of ShhN* or rhShhN found in different fractions, normalised to total ShhN* or total rhShhN found in all fractions. White bar; ShhN*; grey bar; rhShh. Quantification obtained by mean fluorescence of in-gel fluorescence images (for ShhN*) or immunoblotting (for rhShhN), (*n* = 4 for ShhN*; independent replicate experiments, *n* = 1 for rhShhN). Error bars represent ± SD.

To measure multimerised cholesterylated ShhN*, medium from HEK293a Shh+ cells was harvested after 48 hours of treatment with **2** and fractionated by size exclusion chromatography (SEC). Each fraction was immunoprecipitated using anti-Shh antibody 5E1, ligated to **AzTB**, and visualised (Fig. 6A). These experiments showed that >80% ShhN* is found in higher order multimeric complexes, with most found in fractions up to 150 kDa in size (57%) and smaller amounts in multimers up to 450 kDa (21%) or above (∼2.5%), while *E. coli*-derived, recombinant, human ShhN protein (rhShhN) lacking lipid modifications was found exclusively in the monomer fraction (<25 kDa) (Fig. 6B).

Most of the fractionated ShhN molecules were present in the ShhN* form as anti-Shh immunoblotting showed only a faint band representing endogenously processed ShhN, suggesting that ShhN* is the predominant species of processed Shh found in the medium.

Probe **2** thus provides visualisation and quantitative analysis of multimeric species of ShhN containing a C-terminal sterol modification. The data above suggest that cholesterol is present in most of the soluble ShhN released from signalling cells in the model system used, and these species are efficiently assembled into functional ShhN multimers.

### Characterisation of ShhN* in pancreatic cancer cell lines

**Fig. 7:**
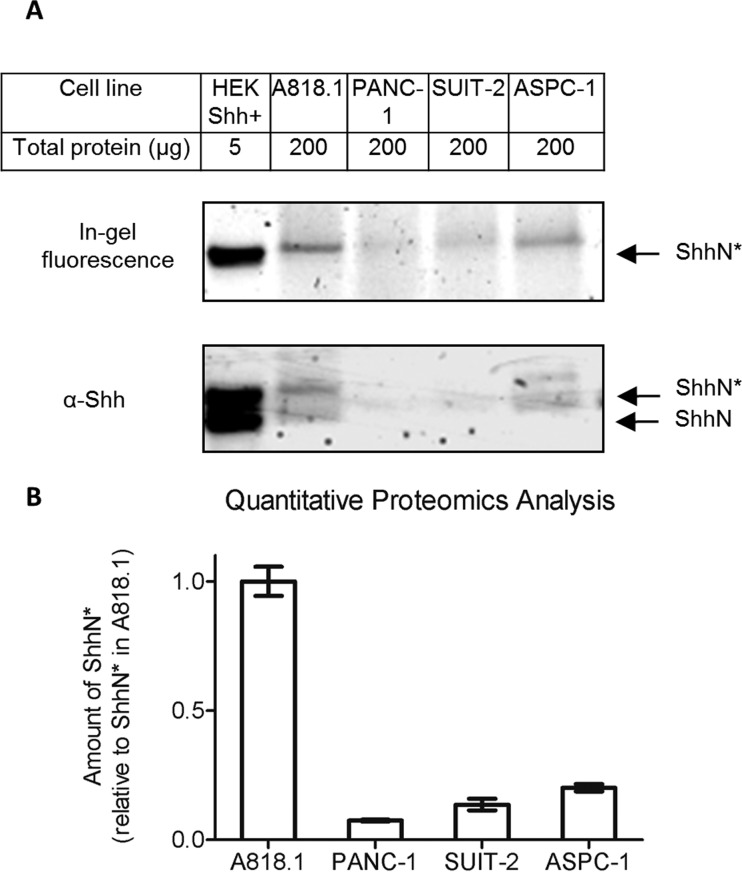
**2** Enables highly sensitive quantitation of ShhN* in a range of pancreatic cancer cell lines by quantitative proteomics. Cells were metabolically labelled with 5 μM **2** for 16 hours, and lysed. (A) Cell lysate immunoprecipitated using anti-Shh antibody was labelled with **AzTB**, proteins separated by SDS-PAGE and imaged (in-gel fluorescence and immunoblot). ShhN*, ShhN tagged with alkynyl-cholesterol analogues; ShhN, untagged, processed Shh protein. (B) The cell line indicated was treated with 5 μM **2** for 16 hours, and the lysate (without immunoprecipitation) was spiked in the ratio 100 : 1 (based on total protein content) with lysate from HEK293a Shh+ cells cultured in heavy isotope-containing SILAC medium (R10K8) in the presence of **2**. The spiked lysates were labelled with **AzTB**, affinity purified on NeutrAvidin agarose beads and subjected to on-bead digest and proteomic analysis (nanoLC-MS/MS). Total lysate analysed in each sample was 300 μg. Quantification represents the mean SILAC ratio of sample to HEK293a Shh+ R10K8, standard normalised to the mean value obtained for A818.1 cells (*n* = 3 independent replicate experiments). Error bars represent ± SD.

We next sought to determine whether **2** could be used to study the presence and quantify the level of ShhN* in different cell lines expressing endogenous levels of Shh, as opposed to an overexpression system (HEK293a Shh+ cells). For this study we selected pancreatic ductal adenocarcinoma (PDAC) cells, since Shh was found to be expressed in 70% of PDACs (normal pancreas samples express no detectable Shh),^40^ where it is involved in increased proliferation, survival and invasion.^41,42^ Thus, four different PDAC cell lines: PANC-1, A818.1, SUIT-2 and ASPC-1, were incubated with **2**. All of these lines express Shh transcripts (Fig. S2†).^43,44^ Cells were treated with 5 μM **2** for 16 hours, followed by lysis and CuAAC ligation to **AzTB**. In-gel fluorescence and immunoblotting (Fig. 7A) showed that the amount of ShhN* in these cancer cell lines is dramatically lower than in HEK293a Shh+ cells; nevertheless, ShhN* was visible by in-gel fluorescence and immunoblotting in A818.1 and ASPC-1. A faint fluorescent band was also found for PANC-1 and SUIT-2 cells; however, these lines did not give a signal for ShhN by Western blot, suggesting that ShhN expression in these cells is very low, and that fluorescence imaging enabled by **2** provides a superior detection limit to immunoblotting with an anti-Shh antibody. However, these data provided only a qualitative idea of the presence of ShhN* in these cells, and we next explored the potential of **2** to enable quantitative determination of ShhN* levels through chemical proteomic analysis. Cancer cell line lysate was combined with 1% (by total protein content) of lysate originating from HEK293a Shh+ cells metabolically labelled with ^13^C_6_^15^N_4_-arginine and ^13^C_6_^15^N_2_-lysine (R10K8) during incubation for 16 hours with 5 μM **2**; this material acts as an internal ‘spike-in’ standard for comparative quantification.^45^ Shh processing or labelling with **2** was unaffected by SILAC (Stable Isotope Labelling with Amino acids in Cell culture) labelling of HEK293a Shh+ with R10K8 as shown by in-gel fluorescence or immunoblotting (Fig. S3†). The mixed lysate was further treated with **AzTB**, affinity purified on NeutrAvidin® beads, and subjected to on-bead tryptic digest and quantitative proteomic analysis by nanoLC-MS/MS (Fig. 7B and SI Table 2†). Multiple unique ShhN* peptides were found in all samples, demonstrating the capacity of **2** to label endogenous ShhN even at very low native levels in diverse pancreatic cancer cell lines. For quantification, relative abundance was obtained by reference to the spike-in standard, and then normalised to the ShhN* abundance in A818.1 cells, which express the highest level of ShhN. The relative amounts of ShhN* found in ASPC-1, SUIT-2 and PANC-1 were 0.202 (±0.014), 0.136 (±0.023) and 0.075 (±0.004), respectively, consistent with the qualitative trends from in-gel fluorescence and immunoblotting analysis of the same samples.

These data highlight the power of probe **2** to enable quantitative mass-spectrometric analysis of ShhN labelling, and provide an insight into previously unknown and subtle differences in expression levels of lipidated ShhN in PDAC cell lines.

### Single cell imaging of probe **2**

**Fig. 8:**
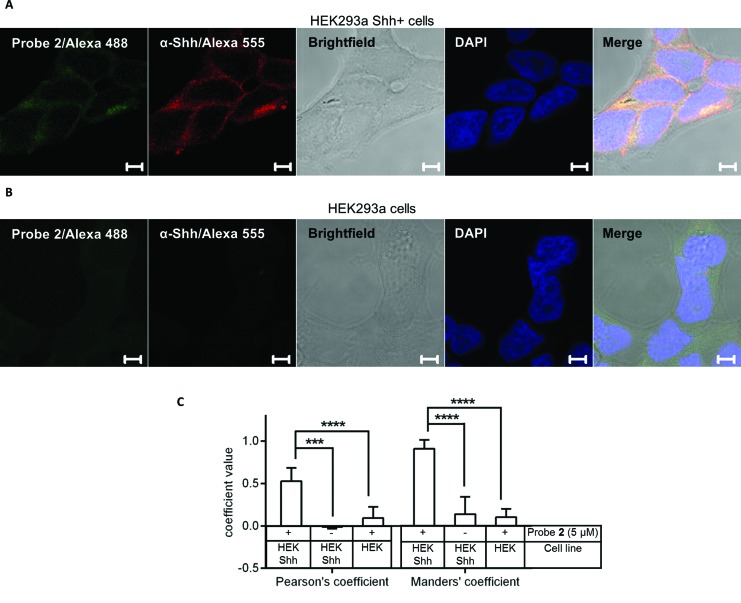
Imaging probe **2** in cells by fluorescence microscopy. HEK293a Shh+ cells (A) or HEK293a cells (B) were metabolically labelled with 5 μM **2** for 16 hours, fixed with 3% PFA, permeabilised with –20 °C MeOH, and labelled with AlexaFluor488 by CuAAC (20 μM az-Alexa488, 1 mM TCEP, 100 μM TBTA and 1 mM CuSO_4_), counterstained with rabbit anti-Shh antibody followed by AlexaFluor555-conjugated goat anti-rabbit, and DAPI (nuclear stain), mounted and imaged with a Zeiss LSM-510 Inverted laser scanning confocal microscope. Green channel, labelling with **2**/AlexaFluor488; red channel, anti-Shh/AlexaFluor555-rabbit antibody; white channel, brightfield; blue channel, nuclear/DAPI staining; merge of all channels. Scale bar represents 5 μm. (C) Co-localisation analysis by Pearson's correlation (P) and Manders' (M1) coefficient for green channel (labelling with **2**). Quantification represents mean coefficient value calculated for multiple cells: HEK293a Shh+ **2** (*n* = 27); HEK293a Shh+ DMSO (*n* = 23); HEK293a **2** (*n* = 28). Error bars represent ± SD, *p* value measured from one-way ANOVA, (***) < 0.001, (****) < 0.0001.

Finally, the high signal to background shown by in-gel analysis of ShhN* labelling by **2** encouraged us to explore probe visualisation in the context of a single cell by fluorescence microscopy. HEK293a Shh+ cells were labelled with probe **2** or vehicle control (DMSO), fixed with paraformaldehyde, permeabilised with methanol (MeOH), and finally subjected to CuAAC ligation to azido-Alexa488 followed by confocal fluorescence microscopy. The fluorescence signal obtained in cells treated with probe was significantly higher than DMSO control (Fig. 8A and S5A†). It was also higher than signal found in untransfected HEK293a cells treated with the probe, not natively expressing Shh (Fig. 8B). A comparison between non-permeabilised and MeOH-permeabilised cells showed a significant decrease in fluorescence upon MeOH treatment (Fig. 8A and S5B†), demonstrating that treatment with MeOH is an effective method to extract unbound lipid from cell membranes, in agreement with previous studies on acyl-modified proteins.^31^ However, it should also be pointed out that HEK293a cells showed low, but detectable fluorescent signal, suggesting that either MeOH permeabilisation did not fully remove the unbound sterol present or that other unidentified sterylated proteins are labelled by the probe, whose expression levels are below the detection limit of in-gel fluorescence imaging but are detectable by fluorescence microscopy. We next investigated the localisation of **2** in HEK293a in Shh+ by immunostaining with Shh antibody prior to fluorescence microscopy imaging (Fig. 8A, S5A and C†).

The results obtained from imaging over 20 individual cells in three different experiments (HEK293a Shh+ treated with **2**, HEK293a treated with **2** or HEK293a Shh+ treated with DMSO) were taken for further analysis to determine standard colocalisation coefficients (Pearson and Manders) between Alexa488 (green channel) and anti-Shh-Alexa555 (red channel) (Fig. 8B). Pearson's coefficient reflects mutual colocalisation through the spread of distribution of pixels within two different channels determined by the intensity of a pixel present in one channel *versus* intensity of a corresponding pixel in the second channel, where 1.0 represents absolute mutual colocalisation.^46^ The coefficient of 0.53 (±0.08) obtained for HEK293a Shh+ cells incubated with **2** was significantly higher than for controls, and showed that whilst **2** might be highly colocalised with Shh, Shh is only partially colocalised with **2**, consistent with partial labelling of ShhN* (Fig. 3). Manders' coefficient represents colocalisation on a per-channel basis, through the sum of pixel intensities in a channel for which the intensity of pixels in a second channel is above zero, divided by total pixel intensities in the first channel for each channel separately, where 1.0 represents absolute colocalisation.^46^ Manders' coefficient was calculated for the green channel in order to analyse the degree to which probe labelling colocalised with Shh. In HEK293a Shh+ cells treated with **2** a value of 0.91 (±0.05) was obtained, demonstrating a high level of colocalisation of **2** with Shh and suggesting that the image of probe **2**, which could in principle visualise all sterylated proteins in the cells, is highly correlated with Shh expression in the same cells.

### Labelling sterylated Hh proteins (HhN*) *in vivo*

**Fig. 9:**
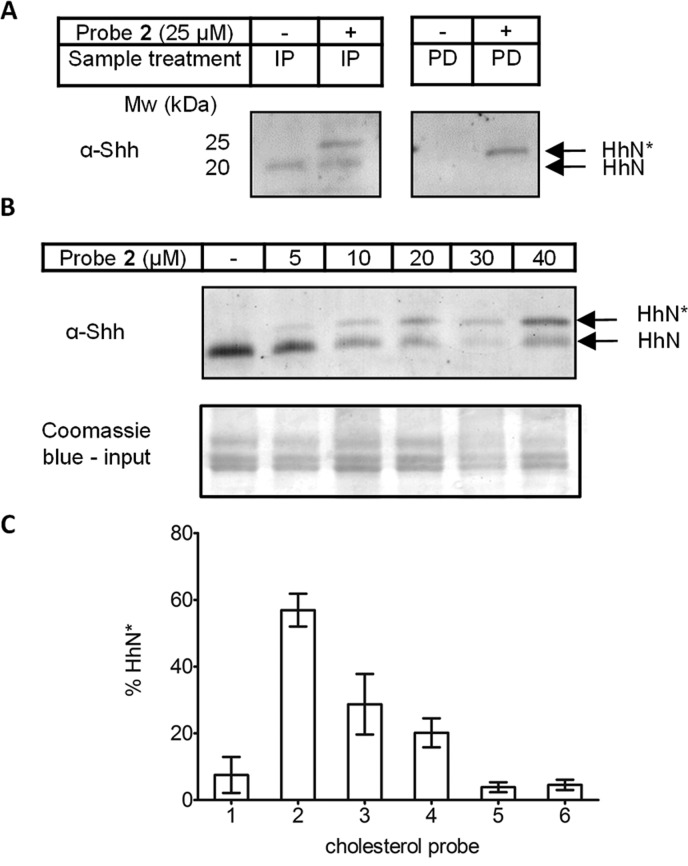
Probe **2** enables the visualisation of sterylated Hh proteins in zebrafish embryos. Fertilised zebrafish eggs were collected and dechorionated (4–5 hpf) and placed in zebrafish water containing DMSO or (A) 25 μM **2**, (B) specified concentration of **2**, or (C) 25 μM of probes **1–6**, for 43–44 hours. After this time embryos were lysed. (A) Whole organism lysate (200 μg of total proteins) was immunoprecipitated using α-Shh antibody and Pureproteome protein G magnetic beads and labelled with **AzTB** on resin [IP] or ligated to **AzTB** and affinity purified using streptavidin-coated magnetic beads [PD]. (B and C) Whole organism lysate (200 μg of total proteins) was immunoprecipitated using α-Shh antibody and Pureproteome protein G magnetic beads and labelled with AzTB. Supernatant from immunoprecipitation was kept as a loading control. After treatment, proteins were separated by SDS-PAGE and imaged by Western blotting (Coomassie blue of the IP input; loading control). HhN*, HhN tagged with alkynyl-cholesterol analogues; HhN, untagged, processed Hh proteins. (C) Analysis of total amount of HhN* normalised to total processed Hh (HhN* + HhN), quantified by chemiluminescence following immunoblot (Fig. S4E†) with α-Shh antibody (*n* = 3, error bars ± SD).

Probe **2** was proven to label Hh protein in cell-based assays; we next explored whether **2** can label Hh in zebrafish embryos (*Danio rerio*). Zebrafish are an attractive model to study vertebrate development,^47,48^ because of their fast, well-defined and highly conserved developmental processes and high fecundity. Furthermore, zebrafish express five different members of the Hh family: Sonic homolog a and b (also known as Tiggy Winkle), Indian homolog a and b (also known as Echidna) and Desert Hedgehog. To perform such an experiment, fertilised eggs obtained from breeding wild-type adult zebrafish were dechorionated 4–5 hours post-fertilisation (hpf) and kept in a solution of zebrafish water containing 25 μM of **2** for 43–44 hours. Following treatment, embryos did not suffer any visible phenotypic changes (Fig. S4A–D†) and viability was comparable to embryos treated with vehicle control (DMSO) for up to the 48 hpf tested. After this time embryos were lysed and the whole-animal lysate was collected, subjected to immunoprecipitation (IP) using 10H6 anti-Shh antibody (Abcam®), ligated to **AzTB**, and visualised (Fig. 9A, left panel). Similarly to the cell-based assays the immunoblot image showed two bands (∼23 and 19 kDa) in the samples treated with probe **2**, in contrast to only a single immunoblot band (at ∼19 kDa) observed in control (vehicle-treated) embryos. This suggested successful visualisation of Hedgehog proteins in zebrafish lysate and furthermore, successful labelling of sterylated Hh by **2** *in vivo*. To confirm this finding, affinity purification of all biotinylated proteins with streptavidin-coated beads was performed directly on whole embryonic lysate following CuAAC ligation with **AzTB** (Fig. 9A, right panel). This treatment revealed in the immunoblot with anti-Shh antibody that only the higher molecular weight Hh band in sample treated with **2** is biotinylated, and was therefore assigned as HhN*. Our further studies focused on the concentration dependence of HhN* labelling with probe **2** in zebrafish embryos (Fig. 9B). Labelling of HhN* increased with concentration of **2** up to 40 μM. However, even 5 μM **2** was sufficient to obtain a detectable signal of HhN* by immunoblotting. Interestingly, a higher working concentration of probe **2** could be achieved in zebrafish water *versus* cell culture medium.

Finally, we were also interested to explore the potential for other sterol probes (**1–6**) to label HhN*. Fertilised, dechorionated embryos (4–5 hpf), were placed for up to 48 hpf in zebrafish water containing 25 μM of each compound (Fig. S4E†). This study was performed in its entirety on three separate days to obtain a quantitative comparison of HhN* with total HhN found by immunoblotting (Fig. 9C). Remarkably, the results showed that labelling of HhN* in whole organism lysate closely resembled the pattern of labelling efficiency obtained in cell culture experiments, where probe **2**-treated samples achieved the greatest probe incorporation (57% of total HhN) under the conditions used, followed by probes **3** and **4**. Probe **1** showed a very faint band of HhN* in immunoblotting, which was further quantified as 7.5% of total HhN, while probes **5** and **6** showed barely detectable HhN*.

All of these data highlight the power of probe **2** to label Hh proteins not only *in vitro* but also *in vivo* in developing zebrafish embryos. Furthermore, to our knowledge this study is the first successful example of studying protein lipidation, and one of only a few reports of posttranslational modification,^49–52^ *in vivo* using a metabolic chemical tagging approach, combined with bioorthogonal ligation reaction in a living organism.

## Conclusions

Here we have described the design and characterisation of new tools to study Shh cholesterylation. The optimal molecule, **2**, shows superior selectivity and sensitivity, labelling ShhN at a probe concentration at least 100-fold lower than a previously reported azide-tagged cholesterol analogue, and with substantially greater labelling efficiency.^22^ Importantly, **2** also permits highly sensitive labelling and detection, with very low background in in-gel fluorescence based studies. These significant advantages may derive in part from use of an alkyne tag, which previous reports suggest may provide superior signal-to-background,^31,32^ increased specificity or affinity for Shh, and improved uptake into cells. Furthermore, **2** showed no signs of toxicity within concentration ranges useful for labelling both in cell based studies, and in zebrafish embryos up to 48 hpf. Building on these useful properties, probe **2** enabled the first *in situ* studies of Shh sterylation in Shh complexes, and multiple assays were used to demonstrate that it preserves the key mechanistic and signalling properties of ShhN in cell culture. These include incorporation into ShhN* through the formation of an ester bond at an early stage following protein synthesis, release from the signalling cell, formation of ShhN multimeric complexes, and activation of transcription of Shh target genes.

To demonstrate the potential utility of **2**, we have presented a number of new insights that can derived from studying ShhN* *in situ*, in contrast to previous studies looking at loss of function (*i.e.* absence of lipidation) on mutation of Shh. For example, **2** was used to provide evidence that cholesterylated ShhN exists in high molecular weight extracellular complexes, as well as to determine the preferred size range of transport complexes that incorporate ShhN*. Furthermore, labelling with **2** provided the first quantitative determination of the relative amount of ShhN* between four pancreatic cancer cell lines, showing significant variation. In future it will be interesting to compare these differences with the ability of each cell line to signal in a paracrine or autocrine fashion, reflecting the distance that the signal can travel. The mode of Hh signalling is thought to be an important factor in clinical PDAC since it determines the degree of communication between the tumour and its environment. In imaging experiments in single cells, robust and sensitive fluorescent detection of **2** was significantly colocalised with ShhN. Although the limited resolution of confocal microscopy prevents direct detection of covalent ShhN modification in cells, these data are consistent with the degree and specificity of labelling observed by in-gel fluorescence. Finally, we have shown successful visualisation of lipidated Hedgehog proteins for the first time in a developing vertebrate organism.

In summary, we have shown here that **2** is a powerful and highly biomimetic tool for studying C-terminal ShhN lipidation. We anticipate that it will find particular utility in exploring the role of lipidation in secretion and intercellular trafficking of members of the Hedgehog protein family. Furthermore, its low toxicity and high labelling efficiency allow probing ShhN* *in vivo* in the context of a developing organism or cancer model, studies that are currently on-going in our labs.
