# Supplementary material for: New chemical probes targeting cholesterylation of Sonic Hedgehog in human cells and zebrafish
Source: Chem Sci. 2014 Aug 15;5(11):4249–59. doi: 10.1039/c4sc01600a (PMC4285107; doi:10.1039/c4sc01600a)
Supplement: Supplementary file 1 [file SC-005-C4SC01600A-s001.pdf]

Electronic Supplementary Material (ESI) for Chemical Science.
This journal is © The Royal Society of Chemistry 2014

New chemical probes targeting cholesterylation of Sonic Hedgehog in human cells and zebrafish

Paulina Ciepla,[a,b] Antonios D. Konitsiotis,[c] Remigiusz A. Serwa,[a] Naoko Masumoto,[a,b] Wai P. Leong, [a] Margaret J. Dallman, [b,d] Anthony I. Magee, * [b,c] and Edward W. Tate, * [a,b]

[a] Department of Chemistry, Imperial College London, Exhibition Road, London SW7 2AZ, UK

[b] Institute of Chemical Biology, Imperial College London, Exhibition Road, London SW7 2AZ, UK

[c] National Heart and Lung Institute, Imperial College London, Exhibition Road, London SW7 2AZ, UK

[d] Department of Life Sciences, Imperial College London, Exhibition Road, London SW7 2AZ, UK

Supplementary Information

Contents:

S2 Table S1: Summary of significance (p value) for graph Fig. 3 C.

S2 Table S2: Summary of results of proteomic analysis of PDAC cell lines treated with probe 2.

S3-5 Supplementary Figures

S6-25 Experimental Details

S26-41 NMR spectra of compounds 1-6

S42-45 Full gel images

S46 References

Table S1 Summary of significance (p value) for Fig. 3 C. Comparison of incorporation of novel alkynyl-cholesterol analogues into ShhN in HEK293a Shh+ cells. Analysis of total amount of ShhN* normalized to total processed Shh (ShhN*+ShhN), quantified by chemiluminescence following Western blot with α-Shh antibody (n=5, error bars ± SD). Significance (p value) measured by one-way ANOVA.

Samples	Mean Diff.	95% CI of diff.	Significant?	Summary	Adjusted P Value
1 vs. 2	-27.76	-37.29 to -18.23	Yes	****	< 0.0001
1 vs. 3	-14.69	-24.23 to -5.160	Yes	***	0.0005
1 vs. 4	-3.686	-13.22 to 5.848	No	ns	0.9796
1 vs. 4	-3.686	-13.22 to 5.848	No	ns	0.9796
1 vs. 5	7.913	-1.622 to 17.45	No	ns	0.1791
1 vs. 6	3.611	-5.923 to 13.15	No	ns	0.983
2 vs. 3	13.07	3.531 to 22.60	Yes	**	0.0021
2 vs. 4	24.07	14.54 to 33.61	Yes	****	< 0.0001
2 vs. 5	35.67	26.14 to 45.21	Yes	****	< 0.0001
2 vs. 6	31.37	21.84 to 40.90	Yes	****	< 0.0001
3 vs. 4	11.01	1.474 to 20.54	Yes	*	0.0139
3 vs. 5	22.61	13.07 to 32.14	Yes	****	< 0.0001
3 vs. 6	18.31	8.771 to 27.84	Yes	****	< 0.0001
4 vs. 5	11.6	2.065 to 21.13	Yes	**	0.0082
4 vs. 6	7.298	-2.237 to 16.83	No	ns	0.2739
4 vs. 6	7.298	-2.237 to 16.83	No	ns	0.2739
5 vs. 6	-4.301	-13.84 to 5.233	No	ns	0.9292

Table S2 Summary of results of proteomic analysis of PDAC cell lines treated with probe **2**. The cells were treated with **2** (5 µM) for 16 hours in standard culture medium (S). Cells were lysed and the lysates (without immunoprecipitation) were spiked in the ratio 100:1 (based on total protein content) with an internal standard lysate derived from HEK293a Shh+ cells cultured in heavy (H) isotope-containing SILAC medium ($^{13}C_6{}^{15}N_4$-arginine and $^{13}C_6{}^{15}N_2$-lysine) in the presence of **2** (5 µM). The spiked lysates were labelled with **AzTB**, affinity purified on NeutrAvidin agarose beads and subjected to on-bead digest and proteomics analysis (nanoLC-MS/MS). Total lysate analysed in each sample was 300 µg.

Cell line	A818.1			ASPC-1			PANC-1			SUIT-2		
Sample	1	2	3	1	2	3	1	2	3	1	2	3
Ratio H/S	2.107	1.882	1.988	0.373	0.402	0.428	0.157	0.145	0.142	0.259	0.321	0.234
Unique peptides	7	8	8	7	8	6	5	7	5	7	7	6
Average of H/S ratio	1.992			0.402			0.149			0.272		
StDev of H/S ratio	0.113			0.028			0.008			0.045		

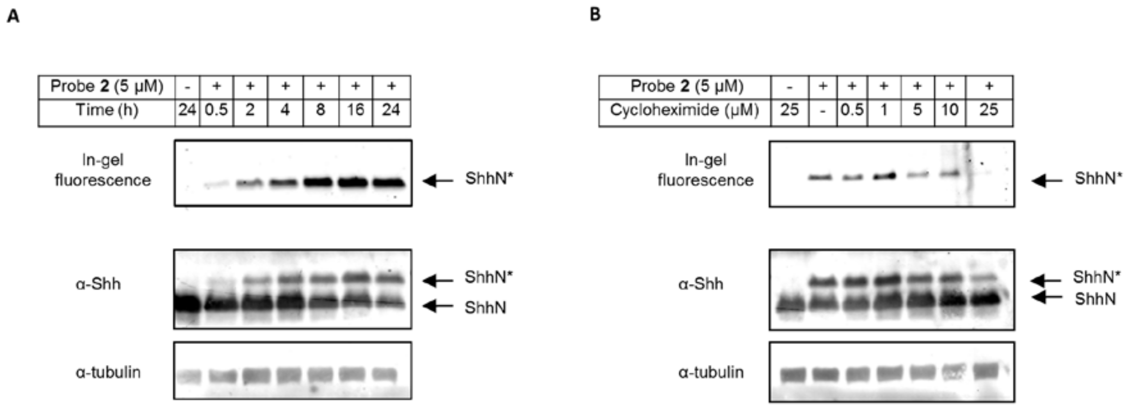

Fig. S1 Validation of **2** as an optimized ShhN cholesterylation probe. (A) Progressive incorporation of **2** into Shh over time in cells. HEK293a Shh+ cells were treated with 5 μM **2** for the period indicated, lysed and labelled with **AzTB**. Proteins were separated by SDS-PAGE and imaged by in-gel fluorescence or immunoblot against Shh or tubulin (loading control). (B) Incorporation of **2** into Shh is dependent on *de novo* protein synthesis. HEK293a Shh+ cells were treated with varying concentrations of cycloheximide for 1 hour prior to treatment with 5 μM **2** and cycloheximide for 16 hours, then lysed and labelled with **AzTB**. Proteins were separated by SDS-PAGE and imaged by in-gel fluorescence or immunoblot against Shh or tubulin (loading control). ShhN*: ShhN tagged with alkynyl-cholesterol analogues; ShhN: untagged, processed Shh protein.

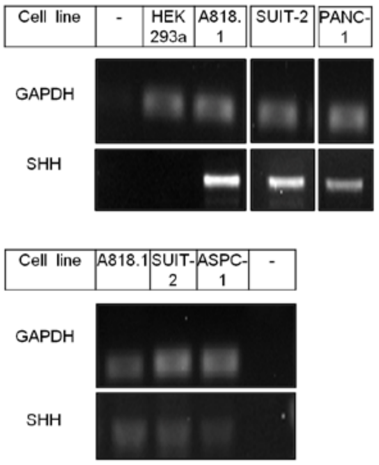

Fig. S2 Pancreatic ductal adenocarcinoma cell lines express Shh transcripts. Cells from 5 different cell lines (HEK293a – negative control; A818.1, ASPC-1, PANC-1 and SUIT-2) were cultured until subconfluent. RNA was extracted, followed by cDNA synthesis and polymerase chain reaction (PCR) with specific primers for human GAPDH (loading control) and Shh. The PCR product was resolved by electrophoresis on 1.7% agarose gel and visualized by ethidium bromide staining. Two experiments were performed on two separate days.

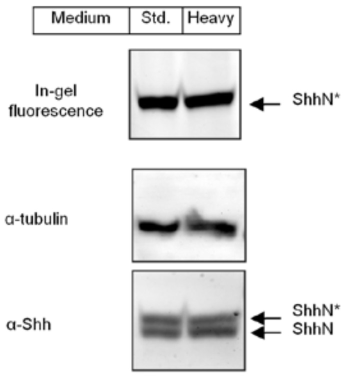

Fig. S3 Shh expression and labelling is unaffected by culture in heavy isotope medium. HEK293a Shh+ cells treated with standard medium or defined heavy medium (R10K8) after 5 passages were metabolically labelled with 5 µM **2** for 16 hours. Cells were lysed and labelled with **AzTB**. Proteins were separated by SDS-PAGE, and imaged (in-gel fluorescence or immunoblot with anti-Shh or anti-tubulin (loading control) antibodies). ShhN*, ShhN tagged with alkynyl-cholesterol analogues; ShhN, untagged, processed Shh protein.

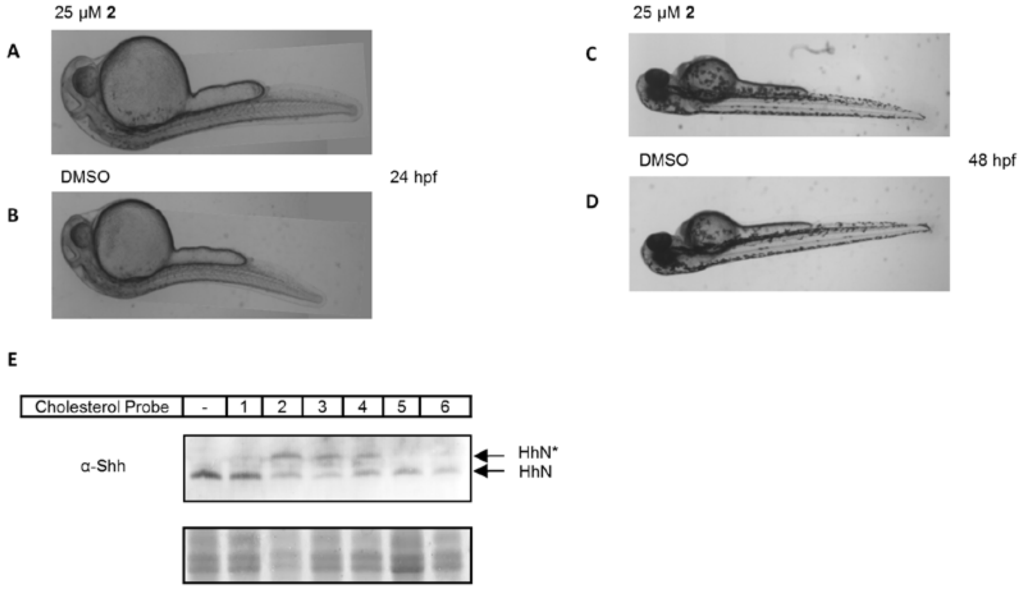

Fig. S4 Probe **2** enables the visualisation of sterylated Hh proteins in zebrafish embryos and their development is unaffected by the probe treatment. Fertilised zebrafish eggs were collected and dechorionated (4-5 hpf) and placed in zebrafish water containing (B and D) DMSO or (A and C) 25 µM **2** or (C) 25 µM of probes **1**-**6**, for 43-44 hours. (A-D) No visible phenotypic changes were observed between zebrafish embryos treated with DMSO (B and D) or 25 µM **2** (A and C) up to 24 hpf (A and B) or 48 hpf (C and D). The images shown are a montage of two separate images of the same embryo. (E) After 43-44 hours of treatment embryos were lysed. Whole organism lysate (200 µg of total proteins) was immunoprecipitated using α-Shh antibody and Pureproteome protein G magnetic beads and labelled with **AzTB**. Supernatant from immunopreipitation was kept as a loading control. After treatment, proteins were separated by SDS-PAGE and imaged by immunoblotting (Coomassie blue of the IP input; loading control). HhN*, HhN tagged with alkynyl-cholesterol analogues; HhN, untagged, processed zebrafish Hh protein.

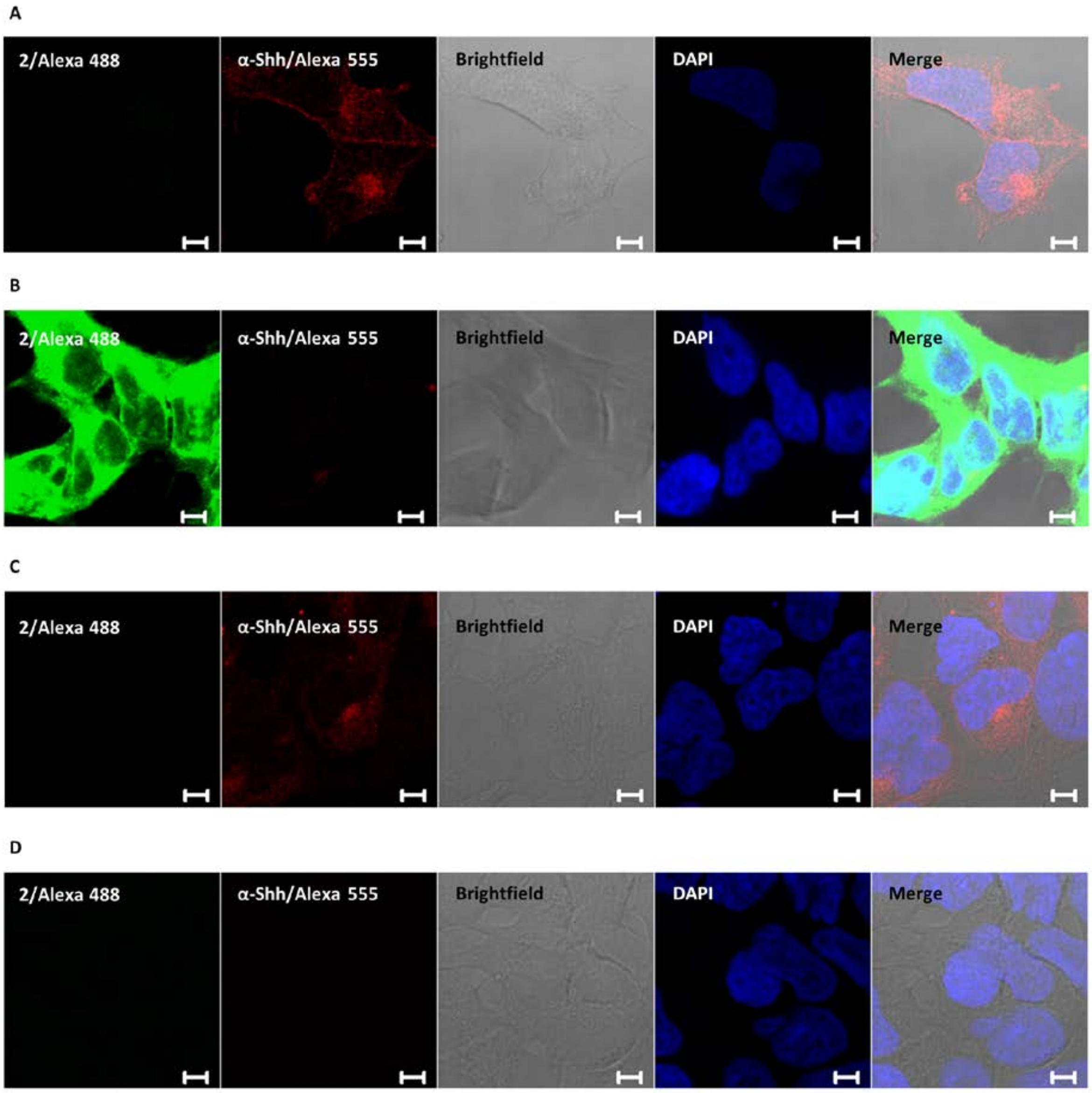

Fig. S5 Imaging **2** in cells by fluorescence microscopy. Cells were metabolically labelled with 5 µM **2** or DMSO for 16 hours, fixed with 3 % PFA, non-permeabilised or permeabilised with -20 ^{0}C MeOH, and labelled with AlexaFluor488 by CuAAC (20 µM az-Alexa488, 1 mM TCEP, 100 µM TBTA and 1 mM $CuSO_4$), counterstained with rabbit anti-Shh antibody followed by AlexaFluor555-conjugated goat anti-rabbit, and DAPI (nuclear stain), mounted and imaged with a Zeiss LSM-510 Inverted laser scanning confocal microscope. Green channel, labelling with **2**/AlexaFluor488; red channel, α-Shh/AlexaFluor555 rabbit anti-Shh antibody; white channel, brightfield; blue channel, nuclear/DAPI staining; merge of all channels. Scale bar represents 5 µm. (A) HEK293a Shh+ cells treated with DMSO. (B) HEK293a Shh+ cells treated with 5 µM **2**, but non-permeabilised. (C) HEK293a Shh+ cells treated with 5 µM **2**, reacted with DMSO instead of az-Alexa 488 and counterstained with anti-Shh rabbit antibody and AlexaFluor555-conjugated goat anti-rabbit. (D) HEK293a Shh+ cells treated with 5 µM **2**, reacted with DMSO instead of az-Alexa 488 and counterstained with AlexaFluor555-conjugated goat anti-rabbit.

Experimental Details

1. Experimental Procedure

Abbreviations:

AMBIC, ammonium bicarbonate; BSA, Bovine serum albumin*;* EDTA, Ethylenediaminetetraacetic acid; FA, Formic Acid; PBS, phosphate buffer saline pH 7.4; PBS-T, PBS containing 0.05% Tween-20; PCR, polymerase chain reaction; TBTA, Tris[(1-benzyl-1H-1,2,3-triazol-4-yl)methyl]amine; TCEP, tris(2-carboxyethyl)phosphine.

1.1 Metabolic labelling of cells with alkynyl-cholesterol analogues (1-6) or alkynyl-palmitate (YnPalm). HEK293a, HEK293a Shh+, A818.1, ASPC-1, PANC-1 and SUIT-2 cells were cultured in DMEM, high glucose content medium, supplemented with 10% foetal bovine serum (FBS). Cells were cultured in a humid, 37°C, 5% CO2 incubator. For labelling of Shh with the cholesterol analogues (**1-6**) or **YnPalm**, 4 x 10^5 or 1.2 x 10^6 cells were seeded in a 6-well plate or 10 cm plate respectively in standard medium. After 6-8 hours, medium was exchanged for feeding medium (DMEM, 3% FBS, and a specified concentration of analogue **1-6** in DMSO or 50 μM **YnPalm** in DMSO, or the same volume of DMSO used as negative control). Cells were harvested and lysed after 16 hours (unless otherwise specified).

1.2 Cell treatment with cycloheximde and metabolic labelling with alkynyl-cholesterol analogue 2. HEK293a Shh+ cells were seeded at 4 x 10^5 cells in a 6-well plate in standard medium. After 6 hours, medium was exchanged for treatment medium (DMEM, 3% FBS containing specified concentration of cycloheximide in DMSO or equal volume of DMSO as negative control); after 1 hour, **2** was also added to the treatment medium (to achieve a final concentration of 5 μM, or equal volume of DMSO as negative control). Cells were harvested and lysed after 16 hours.

1.3 Preparation of heavy amino acids-labelled SILAC HEK293a Shh+ cells. HEK293a Shh+ cells were grown for 5 passages in DMEM supplemented with $^{13}C_6{}^{15}N_4$-arginine and $^{13}C_6{}^{15}N_2$-lysine and 10% dialysed FBS (Dundee Cell Products), before being used for proteomics experiments.

1.4 Preparation of cell lysates. Cells after treatment were harvested by placing on ice and rinsed with ice-cold PBS (2 mL, x 2). Lysis buffer (0.1% SDS, 1% Triton X-100, EDTA-free Complete protease inhibitor (Roche Diagnostics) dissolved in PBS; 100 μL) was added to the cells and incubated for 10 minutes. After lysis, cellular matter was scraped off the surface of the well. DNA was sheared by passing multiple times through a 0.5 mm syringe needle. The lysate was centrifuged at 16,000 x g for 10 minutes to remove insoluble material. The supernatant was collected and used for further experiments or stored at –80 °C.

1.5 Zebrafish stock and husbandry. Fish were maintained according to standard practices and all procedures conformed to U.K. Home Office regulations (ASPA 1986) under Animal Project Licence no. PPL 70/7472. Adult zebrafish strains AB were kept at 28 ºC on 14 hours light and 10 hours dark cycle. Embryos were obtained from natural spawnings and were maintained in System water (combination of tap and RO (reverse osmosis) water).

1.6 Metabolic labelling of zebrafish embryos with alkynyl-cholesterol analogues (1-6). Strain AB zebrafish embryos were placed in zebrafish water (system water containing

0.0001% methylene blue, 25 mL). The embryos were treated as described before in Laughlin *et.al.*[1] with some adjustments. At approximately 4-5 hpf the embryos were enzymatically dechorionated by incubation in 1 mg/mL solution of pronase (Roche Diagnostics) in zebrafish water at room temperature for approximately 7 minutes. The solution with embryos was poured into a beaker with zebrafish water (200 mL) and swirled gently to remove excess Pronase. The solution was removed (not completely) and more zebrafish water was added. The cleaning process was repeated 4 times and during this most of the chorions were also removed. Then, using a Pasteur pipette, the embryos were transferred to into 1% agarose-coated wells of a 48-well plate containing 250 µL of feeding solution (zebrafish water and a specified concentration of analogue **1-6** in DMSO or the same volume of DMSO used as negative control). The embryos were moved to new wells every 24 hours, at the same time the solution was replenished. The solution of compounds and DMSO were prepared fresh every time.

1.7 Preparation of zebrafish lysates. Embryos were euthanized by addition of MS-222 solution (250 mg/L) to each well of 48-well plate. The embryos were then transferred to 1.5ml Eppendorf tubes and were dechorionated using a protocol described previously by Link *et.al.*[2] in a calcium-containing solution. The dechorionated embryos were treated as described previously by Hinz *et.al.*[3] with adjustment. The embryos were washed with ice-cold lysis solution (PBS containing EDTA-free Complete protease inhibitor (Roche Diagnostics, 200 µL). The solution was removed and replaced by fresh lysis solution (50 µL). Zebrafish embryos were homogenised with a Kimble® Kontes Disposable pellet pestle with cordless motor. SDS was added to the lysate to a final concentration of 2% and Benzonase® Nuclease (0.5 µL, Sigma Aldrich, UK) was also added. The lysate was vortexed at room temperature for 5 minutes and heated at 95 ºC for 10 minutes. Lysate was allowed to cool down on ice for 5 minutes, before addition of more lysis solution (50 µL) and Triton X-100 to a final concentration of 0.2%. The lysate was centrifuged at 16,000 x g for 10 minutes at 4 ºC to remove insoluble material. The supernatant was collected and used for further experiments or stored at –80 °C.

1.8 Copper-catalyzed [3+2] cycloaddition (CuAAC) reaction in lysate. Cell or whole organism lysate (50-200 µg of total proteins) was taken and proteins were precipitated by addition of 4 volumes of methanol, 1 vol of chloroform and 3 vols of water. The sample was centrifuged for 5 min at 13,000 rpm and the pellet was washed with methanol. The protein precipitates were resuspended in 2% SDS in PBS and further dissolved to 1 mg/mL of total protein concentration with PBS and final SDS concentration of 0.2%. The samples were reacted with click chemistry reaction cocktail containing **AzTB** (10 mM stock in DMSO) at 100 µM final concentration, $CuSO_4$ (50 mM stock in water) at final concentration of 1 mM, TCEP (50 mM stock in water) at final concentration of 1 mM and TBTA (10 mM stock in DMSO) at final concentration of 100 µM. The reaction was vortexed for 1 hour at room temperature and EDTA (100 mM in water stock) was added to final concentration of 10 mM. Proteins were precipitated by addition of 4 vols of methanol, 1 vol of chloroform and 3 vols of water. The sample was centrifuged for 5 min at 13,000 rpm and the pellet was washed with methanol (2 x). The protein precipitates were resuspended in 2% SDS in PBS and further dissolved to 1 mg/mL of total protein concentration and final SDS concentration of 0.2% with PBS. An aliquot of this sample was taken for SDS-PAGE analysis.

1.9 Affinity purification of biotinylated proteins. The resuspended proteins in 0.2% SDS (50 µL) were incubated with 5 µL Dynabeads (Streptavidin-coupled beads, Invitrogen, UK)

for 1.5 hour on a rotating wheel. The unbound fraction (UB) was collected and then proteins were eluted from the beads by boiling for 10 minutes at 90 °C in 2% SDS and eluted sample (E) was collected. The aliquots of UB and E were taken for SDS-PAGE analysis.

1.10 Immunoprecipitation. 50 μg of proteins from cell lysate (unless otherwise specified), 2 mL of cell culture medium or 2 mL of PBS-eluted SEC fractions were immunoprecipitated with 2 or 5 μg 5E1 anti-Shh Antibody (produced in-house from hybridoma cell line and purified) for cell based experiments or 1 μl of anti-Shh Antibody [10H6] (Abcam, UK) for zebrafish embryo experiments. For each sample, 25 μL of Pureproteome protein-G magnetic beads (Merck Millipore, UK) were used and all samples were incubated overnight at 4 °C on a rotating wheel. The beads were washed (5 x) with lysis buffer and resuspended in 20 μL of PBS and freshly premixed click reaction reagents at appropriate concentrations (as described above) were added (if CuAAC required). After 1 hour of vortex mixing, the beads were washed (5 x) with lysis buffer and mixed with 25% (v/v) of NuPAGE® LDS 4 x sample loading buffer (Invitrogen, UK) and 20% (v/v) 2-mercaptoethanol and boiled for 10 min at 90 °C. After centrifugation, supernatant was collected and used in SDS-PAGE analysis.

1.11 Base Treatment of Gels. After protein separation by SDS-PAGE, the gel was soaked in fixing solution (40% MeOH, 10% acetic acid) for 1 hour, washed with deionised water (3 x 5 min) and scanned for pre-treatment fluorescence. The gel was then soaked in 0.5 M NaOH(aq) (in 40% MeOH) for 2 hours, washed with deionised water, soaked again in fixing solution for 0.5 hour, washed with deionised water (3 x 5 min) and scanned to determine residual labelling.

1.12 SDS-PAGE. Separation of proteins was performed by SDS-PAGE (sodium dodecyl sulfate-polyacrylamide gel electrophoresis), using 15% Bis-Tris gels and 10 x MOPS SDS running buffer, containing: 1M Tris base, 2% SDS and 20 mM EDTA (AGTC Bioproducts, UK) diluted to 1x in water. All samples were prepared by boiling for 10 minutes at 90 °C in 25% (v/v) of NuPAGE® LDS 4 x sample loading buffer (Invitrogen, UK) and 20% (v/v) 2-mercaptoethanol. The protein ladder used for comparison of molecular weight was Precision Plus Protein® Standards All Blue (Bio-Rad, UK). All gels were run using the Mini-PROTEAN® Tetra Cell System (Bio-Rad, UK) and power supply unit (Bio-Rad, UK) at 70-140 V. The typical run took 80 minutes. Fluorescently-tagged proteins were imaged (540 nm excitation and 595 nm emission, channel Cy3) using an Ettan DIGE Imager (GE Healthcare) and images were analysed with ImageQuantTM TL software. Gels were also subject to Coomassie Brilliant Blue R-250 staining or immunoblotting.

1.13 Shh mRNA expression. Cells (HEK293a, A.818.1, ASPC-1, PANC-1 and SUIT-2) were cultured in 6-well plates until subconfluent. Subsequently, total RNA extraction was done with Trizol (Invitrogen, UK) according to the manufacturer's instructions. cDNA was synthesized by random priming from 1 μg of total RNA with the GoScript reverse transcriptase kit (Promega, UK). PCR was performed with specific primers for human GAPDH (sense, 5'-TTCATTGACCTCAACTACAT-3'; antisense, 5'-GTGGCAGTGATGGCATGGAC-3'); and human *Shh* (sense, 5'-CGCACGGGGACAGCTCGGAAGT-3'; antisense,5'-CTGCGCGGCCCTCGTAGTGC-3').[4] PCR was done using Taq DNA Polymerase (New England Biolabs Ltd, UK) following the manufacturer's instructions. Cycling conditions of the PCR were: 30 cycles of 30 seconds at 95 °C, 30 seconds at 60 °C (for GAPDH) or 68 ^{o}C (for *Shh*), and 1 minute/Kb at 68 °C. PCR products were resolved by electrophoresis on 1.7% agarose gels. Gels were run using Horizon 11.14 Horizontal Gel Electrophoresis System (Life Technology Inc., UK) and

power supply unit (Kikusi Electronics Corp., JP) at 50-100 V. The typical run took 30 minutes. The DNA was visualized by ethidium bromide staining using UV transillumination in a Molecular Imager ChemiDoc™ XRS System (Bio-Rad, UK) and images were analysed with Image Lab software.

1.14 Immunoblotting. Proteins were transferred from SDS-PAGE gels to a PVDF membrane (Millipore, UK) using a semi-dry transfer unit (TE77X, Hoefer, USA). The electrode buffer (1% SDS, 192 mM glycine and 25 mM Tris (pH 8.3)) was used to soak the blotting paper and membrane before the transfer. The transfer was carried out for 1.5 hours at 1 mA cm-2. The membrane was blocked with blocking solution (5% w/v milk powder (Marvel), 0.002% sodium azide dissolved in PBS) for 1 hour at room temperature and washed with PBS-T. Shh detection was with H-160 primary antibody (1:200 in 5 mL blocking solution, rabbit polyclonal IgG, Santa Cruz Biotechnology, UK); for α-tubulin detection we used anti-α-tubulin primary antibody (1:5000 in 5 mL blocking solution; mouse monoclonal IgG1, Santa Cruz Biotechnology, UK). Antibody was applied overnight at 4°C followed by washing with PBS-T (4 x 10 mL for 5 minutes at room temperature). Secondary antibody (Shh – 1:20,000 in 5 mL PBS-T, goat anti-rabbit IgG (HRP), Southern Biotech, UK; or 1:1000 mouse anti-rabbit IgG (HRP) VeriBlot, Abcam, UK; α-tubulin – 1:20,000 in 5 mL PBS-T, goat anti-mouse IgG1 (HRP), Southern Biotech, UK) was applied for 45 minutes at room temperature, followed by washing (5 x 10 mL PBS-T). Visualization was carried out by enhanced chemiluminescence (Pierce ECL2 Western Blotting Substrate, Thermo Scientific, UK) according to manufacturer's instructions and on an Ettan DIGE Imager (GE Healthcare, UK), excitation at 480 nm, emission at 530 nm (channel Cy2). Images were analysed with ImageQuantTM TL software.

1.15 Hh Reporter Assay. Shh Light 2 reporter cells were cultured in standard medium, supplemented with 150 µg/mL Zeocin and 400 µg/mL G418. Cells were seeded at 5 x 10^4 in a 96-well plate in standard medium. After 24 hours, medium was exchanged for conditioned medium collected from HEK293a or HEK293a Shh+ cells treated with **2** (5 µM) or DMSO. After incubation for 30 hours, cellular firefly and Renilla luciferase activities were measured by chemiluminescence using Dual Luciferase® reporter Assay System (Promega, UK) according to manufacturer's instructions and using a FluoStar Optima (BMG LabTech, UK).

1.16 Size Exclusion Chromatography (SEC) of cell culture conditioned medium. HEK293a Shh+ cells were seeded at 1.2 x 10^6 in 10 cm plates in medium (DMEM, 0.5% FBS supplemented with 5 µM **2**). After 24 hours, medium was exchanged for new medium (DMEM, 0.5% FBS supplemented with 5 µM **2**), used to maximize the amount of ShhN* present in the cell culture medium. After 24 hours, medium collected from 2 replicate plates was concentrated using Centrifugal Filter Units (Amicon®-Ultra-4-10K, Merck Millipore). Otherwise, 1 µg of recombinant Human Sonic Hedgehog/Shh (C24II), N-Terminus (R&D Systems, UK) was dissolved in culture medium (DMEM, 0.5% FBS). After 48 hours incubation in a cell culture incubator, medium was collected and concentrated as before. The concentrated fractions were loaded onto a BioBasic SEC 300 filtration column (Thermo Fisher) using an ÄKTA Purification System (GE Life Sciences, UK). Fractions were eluted using PBS and pooled size fractions were subjected to immunoprecipitation. The SEC standard contained: Blue Dextran 10 mg/mL, Apoferritin 10mg/mL, Alcohol Dehydrogenase 10 mg/mL, BSA 10 mg/mL and Cytochrome C 5 mg/mL and was run before each set of samples.

1.17 Proteomics analysis. A total of 1 mg of proteins from cancer cell lines treated with **2** (5 µM) was spiked (1%) with a standard (HEK293a Shh+ cell proteins labelled with $^{13}C_6{}^{15}N_4$-arginine and $^{13}C_6{}^{15}N_2$-lysine, and **2**). The protein mixtures were precipitated and subjected to CuAAC reaction, carried out at 2 mg/mL of protein, and affinity purified using NeutrAvidin beads (50 µL). The enrichment was carried out at a concentration of 1 mg/mL, 0.2% SDS, for 2 h at room temperature (rt) on a vortex mixer. The supernatant was removed and the beads were washed with 1 % SDS in PBS (1 mL, x 3), 4 M urea in 50 mM ammonium bicarbonate (AMBIC) (1 mL, x3), and 50 mM AMBIC (1 mL, x5). After removal of excess wash solution, the sample was reduced with dithiothreitol (5 µL, 100 mM stock in 50 mM AMBIC – final concentration 10 mM) for 30 minutes at 55 °C and allowed to cool to rt. The beads were washed with 50 mM AMBIC (1 ml, x2). Cysteines were alkylated with iodoacetamide (5 µL of 100 mM stock in 50 mM AMBIC - final concentration 10 mM) at rt for 30 min in the dark. The beads were washed with 50 mM AMBIC (1 mL, x 2). The bead-associated proteins were digested with trypsin (5 µg, Sequencing Grade Modified Trypsin from Promega) overnight at 37 °C. The samples were centrifuged and the supernatant separated. The beads were washed with 50 mM AMBIC and centrifuged. The washes were combined with the corresponding supernatants, acidified with 0.5% TFA and stage-tip purified utilizing SDB-XC discs (3M) according to published protocol.[5] Peptides were extracted with 79% acetonitrile in water and dried in a Speed-vac (Thermo Fisher Scientific). Analysis was performed on an Easy nLC-1000 coupled to a Q Exactive mass spectrometer via an easy-spray source (all Thermo Fisher Scientific), using a reverse phase Acclaim PepMap RSLC column (50 cm × 75 µm inner diameter; Thermo Fisher Scientific). The peptides were separated using a 2 h acetonitrile gradient in 0.1 % formic acid at a flow rate of 250 nL/min. The Q Exactive was operated in data-dependent mode with survey scans acquired at a resolution of 75,000 at m/z 200 (transient time 256 ms). The 10 most abundant isotope patterns with charge +2 and higher from the survey scan were selected with an isolation window of 3.0 m/z and fragmented by Higher-energy C-trap dissociation with normalized collision energies of 25. The maximum ion injection times for the survey scan and the MS/MS scans (acquired with a resolution of 17500 at m/z 200) were 250 and 80 ms, respectively. The obtained data were processed using MaxQuant software v1.3.0.5, and the peptides were identified from the MS/MS spectra searched against the database (human proteome+isoforms from Uniprot) using Andromeda search engine.[6] Cysteine carbamidomethylation was selected as a fixed, and methionine oxidation as a variable modification. All other parameters were used as preset in the MaxQuant.

1.18 Fluorescence imaging. HEK293a or HEK293a Shh+ cells were seeded at 6 x 10^5 in 24-well plates on sterile coverslips in standard medium. After 24 hours, medium was exchanged for new medium (DMEM, 3% FBS, and 5 µM of **2** in DMSO, or the same volume of DMSO as negative control). After 16 hours, cells were pre-fixed by addition of paraformaldehyde (PFA, 3%) to a final concentration of 1.5% for 3 min. Cells were washed once with PBS, fixed (3% PFA for 10 min at room temperature) and washed twice with ice-cold PBS. Cells were permeabilised (-20°C MeOH for 10 min) and washed with PBS (3 x 5 min, with gentle agitation). Cells were blocked with 5% BSA for 45 min at rt and washed with PBS (3 x 5 min, with gentle agitation). Cells were treated with freshly pre-mixed CuAACreaction solution (azido-Alexa 488 (10 mM stock in DMSO) at 20 µM final concentration, $CuSO_4$ (50 mM stock in water) at final concentration of 1 mM, TCEP (50 mM stock in water) at final concentration of 1 mM and TBTA (10 mM stock in DMSO) at final

concentration of 100 μM in PBS) for 1 hour at room temperature. Cells were washed (3 x 5 min in 1% Tween-20 and 0.5 mM EDTA in PBS, followed by 2 x 5 min in PBS with gentle agitation) and the sample was ready for antibody labelling. Cells were incubated for 1 hour with anti-Shh antibody (H-160, rabbit IgG; dilution 1:100) in 5% BSA-PBS and washed with PBS (3 x 5 min, with gentle agitation). Samples were further labelled with Alexa 555-conjugated goat anti-rabbit IgG (H+L) (2 mg/mL, dilution 1:500 in 5% BSA, Invitrogen) and DAPI (1:10000 in 5% BSA-PBS, Sigma Aldrich) for 30 min and finally washed with PBS (3 x 5 min, with gentle agitation) and distilled water (2 x 5 min, with gentle agitation), before mounting (ProLong Gold, Invitrogen). Confocal images were collected with a Zeiss LSM-510 inverted laser scanning confocal microscope equipped with a Plan-Apochromat 63 x 1.40 oil Ph3 M27 objective. DAPI, Alexa 488 and Alexa 555 fluorophores were excited with a Diode laser at 405/30 nm, argon laser at 488nm and HeNe1 laser at 543 nm, respectively, and the emission was collected through band pass 445/50 nm, 575-640 nm and 515-565 nm filters, respectively. Images were analysed with Volocity software.

2. Synthesis of sterol analogues (1-6)

General: The reagents used during all synthesis processes were obtained from commercial sources (Sigma-Aldrich, VWR, Fisher Scientific) and used without further purification. Reactions were followed by TLC using aluminium-backed silica plate (Merck, TLC Silica Gel 60, F_{254}) and visualised under UV irradiation at 254 nm or *p*-anisaldehyde staining. Automated flash column chromatography was carried out using a Biotage Isolera One (Biotage GB Limited, Hengoed, UK). The purity of the compound was determined using NMR spectroscopy and accurate mass spectroscopy and AT-IR spectroscopy. ^{1}H, ^{13}C and NOE NMR spectra were measured by a Bruker AM-400 spectrometer in $CDCl_3$ using non-deuterated solvent peaks as an internal standard. Chemical shifts (δ) are reported in parts per million (ppm) and coupling constants (J) are reported in Hertz (Hz). Assignment abbreviations: s (singlet), d (doublet), dd (double doublet), t (triplet), q (quartet), quin (quintet), sept (septet), m (multiplet), ster (steroid proton not further assigned). Mass spectrometry was performed using electrospray ionisation (ESI) on an AUTOSPEC P673 spectrometer by L. Heigh at the Chemistry Department Mass Spectrometry Service at Imperial College London. Infra-red spectroscopy was performed using Spectrum 100 FT-IR Spectrometer with Universal ATR Sampling Accessory (Perkin Elmer).

Abbreviations:

DCM , Dichloromethane; EtOAc, Ethyl acetate; THF, Tetrahydrofurane; TBAF, Tetra-n-butylammonium fluoride.

2.1 Synthesis of Chol-5-en-3β-ol-24-propargyl ether (1)

The synthetic route for compounds **7** and **8** uses the method reported by Bharucha *et al.*.[7]

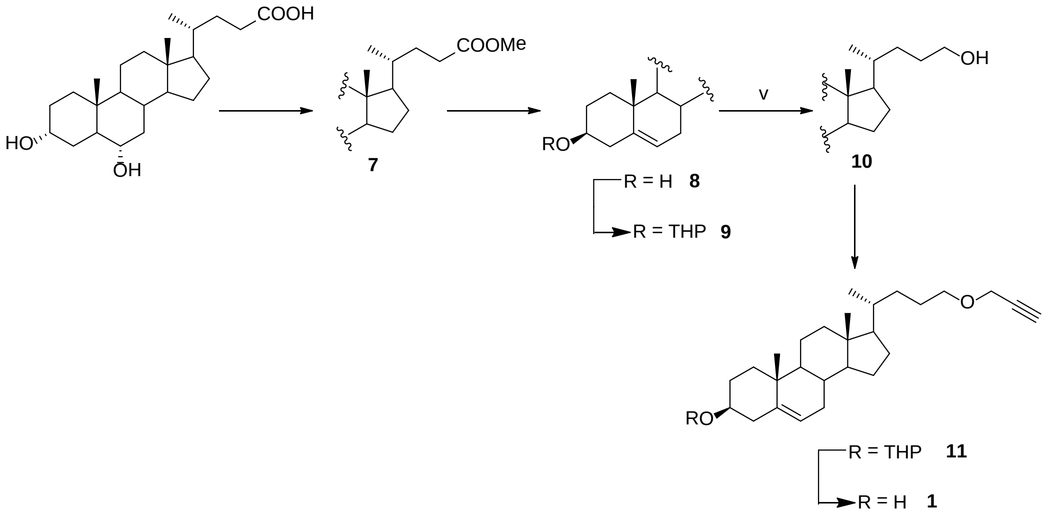

Methyl hyodeoxycholate (7)

Para-toluenesulphonic acid monohydrate (0.45 g, 2.4 mmol) was added to a solution of hyodeoxycholic acid (4.5 g, 11.5 mmol) in dry methanol (35 mL). The reaction was stirred overnight, and then quenched with addition of 0.5 M sodium hydroxide (10 mL) to pH 7. The reaction mixture was extracted with EtOAc and washed three times with saturated sodium bicarbonate. The organic layer was died over $MgSO_4$ and concentrated under reduced pressure. The residue was purified by flash column chromatography (DCM-methanol, 8:2) to afford the white solid (**7**) (3.50 g, 75%). ^{1}H NMR (400 MHz, $CDCl_3$) δ 4.03 (1H; dt, *J* = 4.4, 12.0, 9.6 Hz, *H6*), 3.64 (3H; s, *COOMe*), 3.60 (1H; m; *H3*), 2.37 – 2.15 (2H, m, ster.), 1.96 – 1.73 (5H; m, ster.), 1.60 – 1.53 (4H; m, ster.), 1.48-1.23 (10H, m, ster.), 1.18-0.99 (7H, m, ster.), 0.89 (6H; d, 5.2 Hz; *H21*, *H19*) , 0.61 (3H; s; *H18*); ^{13}C NMR (101 MHz, $CDCl_3$) δ 175.28, 72.06, 68.53, 56,68, 56.42, 52.02, 48.94, 43.34, 40.45, 40.34, 36.45, 36.08, 35.87, 35.42, 35.32, 31.56, 31.45, 30.69, 29.76, 28.63, 24.70, 24.02, 21.26, 18.75, 12.52; IR (neat) 3342, 2932, 2868, 1737, 1445, 1168, 1030, 954, 630 cm^{-1}; HRMS m/z calculated for $C_{25}H_{41}O_3$ $[M\text{-}OH]^+$ 389.3050, found 389.3063.

Methyl 3β-hydroxy-5-cholen-24oate (8)

To the stirring solution of **7** (874 mg, 2.1 mmol) in dry pyridine (7 mL) was added methanesulfonyl chloride (0.5 mL, 6.6 mmol) at rt. After 15 minutes the reaction mixture was taken up in EtOAc and washed with 1.0 M HCl, water and saturated sodium chloride. The organic layer was died over $MgSO_4$ and concentrated under reduced pressure. 1.2 g of the crude product was directly dissolved in DMF (9 mL) and water (1 mL) without further purification. After addition of potassium acetate (160 mg, 1.6 mmol), the mixture was heated at 110 °C overnight, poured into water and extracted three times with EtOAc. The combined extracts were washed twice with saturated sodium chloride, dried over $MgSO_4$ and concentrated under reduced pressure. The residue was purified by flash column chromatography (hexane-EtOAc, 4:1) to afford the white solid (**8**) (275 mg, 33%). ^{1}H NMR

(400 MHz, $CDCl_3$) δ 5.33 (1H, d, J = 5.2, *H6*), 3.64 (3H, s, *COO<u>Me</u>*, 3.50 (1H, septet, J = 4.8, 11.2, 16.0 Hz, *H3*), 2.37-2.15 (4H, m, ster.), 1.99-1.91 (2H, m, ster.), 1.87-1.72 (4H, m, ster.) 1.60-1.22 (11H, m, ster.), 1.17-1.00 (5H, m, ster.), 0.98 (3H, s, *H19*), 0.91 (3H, d, J = 6.4 Hz, *H21*), 0.65 (3H, s, *H18*); ^{13}C NMR (101 MHz, $CDCl_3$) δ 174.81, 140.76, 121.67, 71.78, 56.72, 55.76, 51.52, 50.07, 42.36, 42.29, 39.73, 37.25, 36.49, 35.38, 31.88, 31.64, 31.06, 31.02, 28.12, 24.26, 21.07, 19.41, 18.31, 11.87; IR (neat) 3480, 2935, 2869, 1714, 1431, 1299, 1278, 1257, 1100, 1069, 998, 960, 862, 800 cm^{-1}; HRMS m/z calculated for $C_{25}H_{41}O_3$ $[M+H]^+$ 389.3050, found 389.3061.

Methyl 3β- tetrahydropyranyloxy-5-chol-en-24-oate (9)

3,4-Dihydro-2H-pyran (0.11 mL, 1.21 mmol) was added to a solution of **8**, (150 mg, 0.39 mmol, 1 equiv.) and para-toluenesulphonic acid (5 mg, 0.026 mmol) in dry THF (2 mL) at room temperature. After 24 hours, the reaction mixture was extracted with EtOAc and washed three times with saturated sodium bicarbonate. The organic layer was died over $MgSO_4$ and concentrated under reduced pressure to afford the white solid (**9**) (160 mg, 88%). ^{1}H NMR (400 MHz, $CDCl_3$) δ 5.32 (1H, t, J = 5.6, 11.2 Hz, *H6*), 4.67 (1H, m, *THP*), 3.87 (1H, m, *THP*), 3.62 (3H, s, *COO<u>Me</u>*), 3.46 (2H, m, *THP*, *H3*), 2.35-2.13 (4H, m, ster.), 1.96-1.68 (9H, m, ster.), 1.58-0.99 (20H, m, ster.), 0.96 (3H, s, *H19*), 0.94-0.86 (4H,m, ster., 0.89 (d, J = 6.4 Hz, *H21*)), 0.63 (3H, s, *H18*); ^{13}C NMR (101 MHz, $CDCl_3$) δ 174.83, 121.56, 121.48, 97.02, 96.84, 75.99, 62.98, 56.70, 55.75, 51.52, 50.12, 42.36, 40.23, 39.72, 38.76, 37.19, 36.77, 35.38, 31.87, 31.05, 31.02, 29.68, 28.12, 25.48, 24.26, 21.04, 19.79, 19.39, 18.31, 11.86; IR (neat) 2934, 2867, 1734, 1441, 1168, 1134, 1112, 1059, 1022, 974, 911, 869 cm^{-1}.

3-β-tetrahyropyranyloxy-chol-5-en-24-ol (10)

A solution of lithium aluminium hydride (60.7 mg, 1.6 mmol) in dry THF (2 mL) was added dropwise over 10 minutes to a solution of **9** (92.2 mg, 0.2 mmol) in dry THF (2 mL). After 5 hours the reaction was quenched with saturated sodium bicarbonate, filtered and extracted with EtOAc. After decanting to remove the unwanted lithium hydroxide (purple precipitate), the EtOAc layer was washed with saturated sodium chloride, dried over $MgSO_4$ and concentrated under reduced pressure to afford the white solid (**10**) (82 mg, 94%). ^{1}H NMR (400 MHz, $CDCl_3$) δ 5.32 (1H, t, J = 5.6, 11.2 Hz, *H6*), 4.69 (1H, m, *THP*), 3.89 (1H, m, *THP*), 3.58 (2H, dd, J = 4.4, 10.0 Hz, *H24*), 3.47 (2H, m, *THP*, *H3*), 2.34-2.15 (2H, m, ster., *H17*), 2.00-1.78 (6H, m, ster.), 1.72-1.38 (16H, m, ster.), 1.28-1.00 (8H, m, ster.), 0.98 (3H, s, *H19*), 0.92 (3H, dd, J = 6.4 Hz, *H21*), 0.66 (3H, s, *H18*). ^{13}C NMR (101 MHz, $CDCl_3$) δ 141.07, 140.90, 121.58, 121.51, 97.00, 96.83, 75.99, 63.62, 62.86,56.73, 55.97, 50.15, 42.33, 40.24, 39.75, 37.44, 36.77, 35.60, 31.84, 31.29, 29.68, 29.39, 28.24, 27.97, 25.48, 24.27, 21.04, 20.04, 19.40, 18.69, 11.87; IR (neat) 3300, 2936, 2868, 1735, 1440, 1375, 1199, 1112, 1068, 1023, 974, 911, 869, 809 cm^{-1}; ESI (+) MS m/z found for $C_{29}H_{47}O_2$ $[M-H_2O]^+$ 427.4.

3β-tetrahydropyranyloxy-chol-5-en-24-propargyl ether (11)

The reaction was performed under strictly anhydrous conditions. To a stirring solution of sodium hydride (28 mg 1.2 mmol) in dry THF (1 mL) at 0 °C under nitrogen was added a mixture of **10** (52 mg, 0.1 mol) and 18-crown-6 (217 mg, 0.8 mol) dissolved in dry THF

(2 mL). The reaction was stirred for 30 minutes at 0 °C then propargyl bromide (30 µL, 0.3 mmol) was added and the reaction mixture was warmed up to 50 °C and stirred overnight. After 17 hours the reaction mixture was cooled to rt, extracted with EtOAc and washed twice with water and twice with saturated sodium chloride. The organic layer was died over $MgSO_4$ and concentrated under reduced pressure. The residue was purified by flash column chromatography (hexane-EtOAc, 4:1) to afford the white solid (**11**) (47 mg, 82%). ^{1}H NMR (400 MHz, $CDCl_3$) δ 5.32 (1H, t, J = 6, 11.2 Hz *H6*), 4.68 (1H, d, J = 4.8 Hz, *THP*), 4.10 (2H, d, J = 2.4 Hz, O<u>CH$_2$</u>CCH), 3.88 (1H, m, *THP*), 3.45 (4H, m, *H24*, *THP*, *H3*), 2.38 (1H, t, J = 2.4, 4.0 Hz OCH_2C<u>CH</u>) 2.33-2.12 (2H, m, ster., *H17*), 2.00-1.76 (6H, m, ster.), 1.71-1.34 (17H, m, ster.), 1.25-1.01 (6H, m, ster.), 0.97 (3H, s, *H19*), 0.90 (3H, d, J = 6.8, *H21*), 0.64 (3H, s, *H18*); ^{13}C NMR (101 MHz, $CDCl_3$) δ 141.58, 141.40, 122.04, 121.97, 97.47, 97.32, 80.59, 76.51, 74.53, 71.29, 63.29, 58.47, 57.24, 56.50, 50.67, 42.83, 40.27, 39.28, 37.95, 37.70, 37.26, 36.06, 32.64, 32.39, 31.80, 30.20,28.70, 28.50, 26.60, 26.00, 24.77, 21.55, 20.59, 19.88, 19.13, 12.36.

Chol-5-en-3β-ol-24-propargyl ether (1)

To a stirring solution of **11** (45 mg, 0.09 mmol) in dry DCM/methanol 1:1 mixture (1 mL) was added a solution of para-toluenesulphonic acid monohydrate (2 mg, 0.01 mol) in dry methanol (1 mL) at rt. The reaction was stirred overnight, before the reaction mixture was extracted with DCM and washed three times with water. The organic layer was died over $MgSO_4$ and concentrated under reduced pressure. The residue was purified by flash column chromatography (hexane-EtOAc, 4:1) to afford the white solid (**1**) (17 mg, 46%); ^{1}H NMR (400 MHz, $CDCl_3$) δ 5.32 (1H, d, J = 5.2 Hz, *H6*), 4.10 (2H, d, J= 2.4 Hz, O<u>CH$_2$</u>CCH), 3.45 (3H, m, *H24*, *H3*), 2.39 (1H, t, J = 2.4 Hz, OCH_2C<u>CH</u>) 2.30-2.16 (2H, m, ster.), 2.01-1.76 (5H, m, ster.), 1.68-1.33 (12H, m, ster.), 1.28-1.00 (7H, m, ster.), 0.98 (3H, s, *H19*), 0.91 (3H, d, J = 6.4, *H21*), 0.65 (3H, s, *H18*); ^{13}C NMR (101 MHz, $CDCl_3$) δ 141.27, 122.19, 80.58, 74.58, 72.28, 71.32, 58.49, 57.25, 56.48, 50.59, 42.83, 42.79, 40.26, 37.75, 37.00, 36.06, 32.63, 32.39, 32.14, 28.71, 26.59, 24.78, 21.58, 19.91, 19.13, 12.37; IR (neat) 3422, 3313, 2923, 2852, 1465, 1378, 1104, 1044, 1011, 958 cm^{-1}; HRMS *m/z* calculated for $C_{27}H_{41}O$ $[M-OH]^+$ 381.3152, found 381.3152.

2.2Synthesis of 24-norchol-5,20(22-trans)-dien-3β-ol-23-propargyl ether (2)

The synthetic rout for compounds **13**, **14** and **15**, **16** uses the method reported by Rahman *et al.*,[8], for synthesis of analogues saturated at position C5,6.

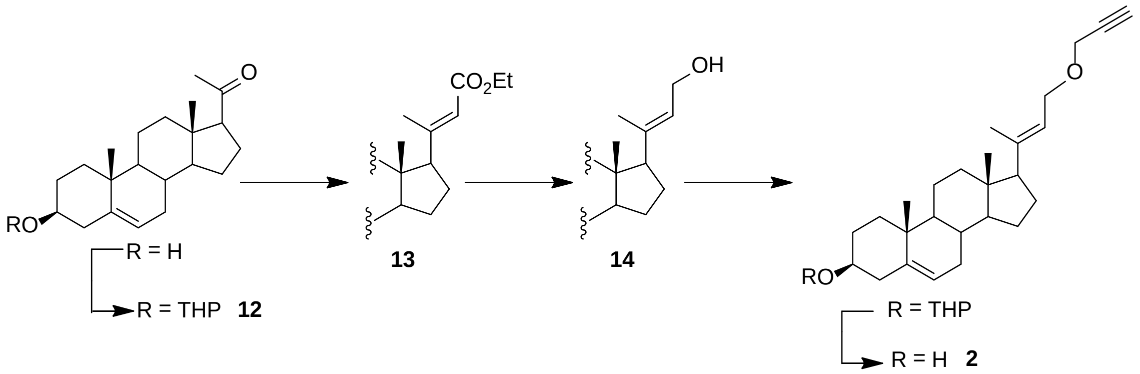

3β-(tetrahydropyranyloxy)pregn-5-en-20-one (12)

To a stirring solution of pregn-5-en-3β-ol-20-one (2.0 g, 6.3 mmol) and para-toluenesulphonic acid monohydrate (50 mg, 0.3 mmol) in dry THF (5 mL), was added dihydropyrane (1.6 mL, 19.0 mmol) at rt. After 17 hours, the reaction mixture was extracted with EtOAc and washed three times with sodium bicarbonate. The organic layer was died over Na_2SO_4 and concentrated under reduced pressure to afford **12** (2.5 g, 99%) as a white solid. ^{1}H NMR (400 MHz, $CDCl_3$) δ 5.32 (1H, t, *J* = 6.0, 11.6 Hz, *H6*), 4.69 (1H, m, *THP*), 3.88 (1H, m, *THP*), 3.47 (2H, m, *THP*, *H3*), 2.51 (1H, t, J = 9.2, 18.0 Hz, *H17*), 2.34-2.13 (3H, m, ster.), 2.09 (3H, s, *H21*), 2.03-1.78 (5H, m, ster.), 1.72-1.38 (14H, m, ster.), 1.25-1.01 (3H, m, ster.), 0.98 (3H, s, *H19*), 0.59 (3H, s, *H18*); ^{13}C NMR (101 MHz, $CDCl_3$) δ 209.75, 141.27, 141.09, 121.46, 121.38, 97.17, 97.00 76.07, 63.91, 63.09, 57.11, 50.21, 44.20, 40.39, 39.04, 37.65, 37.40, 37.00, 32.04, 31.73, 31.45, 29.85, 28.14, 25.68, 24.68, 23.00, 21.25, 20.18 19.56, 13.41; IR (neat) 2932, 2904, 2872, 2848, 1705, 1452, 1439, 1376, 1355, 1199, 1185, 1155, 1134, 1114, 1032, 972, 912 cm^{-1}; HRMS *m/z* calculated for $C_{21}H_{31}O$ [M-OTHP]$^+$ 299.2369, found 299.2384.

Ethyl 3β-tetrahydropyranyloxy-24-norchol-5,20(22)-dien-23-oate (13 – trans and 15 - cis)

To a stirring solution of diisopropylamine (1.9 mL, 13.5 mmol) dissolved in dry THF (20 mL) was added 1.6 M n-butyllithium in THF (8.5 mL, 13.5 mmol) at −78 °C. The reaction was stirred for 15 minutes at −78 °C prior to addition of ethyl(trimethylsilyl) acetate (2.5 mL, 13.5 mmol). After stirring at the same temperature for 30 minutes, a solution of **12** (2.4 g, 6.0 mmol) in dry THF (5 mL) was added, and the mixture was left overnight to slowly warm to rt. After 20 hours, the reaction mixture was extracted with Et_2O and washed three times with diluted sodium bicarbonate. The organic layer was died over $MgSO_4$ and concentrated under reduced pressure to give a crude residue (3.1 g) containing both **13** and **16**. The crude residue (2.0 g) was purified by flash column chromatography (hexane-Et_2O, 4:1) to afford the pure, white solid (**13**) (415 mg, 15 %); ^{1}H NMR (400 MHz, $CDCl_3$) δ 5.67 (1H, s, *H22*), 5.32 (1H, t, *J* = 8.0, 16.0 Hz, *H6*), 4.70 (1H, t, J = 2.0, 6.8 Hz, *THP*), 4.13 (2H, q, J = 6.0, 11.6 Hz, O$\underline{CH_2}$$CH_3$), 3.89 (1H, m, *THP*), 3.48 (2H, m, *THP*, *H3*), 2.35-2.16 (6H,

m, ster., 2.16 (3H, d J = 1.2, *H21*)), 2.00-1.34 (20H, m, ster.), 1.27-0.91 (6H, m, ster., 1.27 (3H, t, J = 7.2, 14.4 Hz, $OCH_2\underline{CH_3}$)) , 0.99 (3H, s, *H19*), 0.57 (3H, s, *H18*); ^{13}C NMR (101 MHz, $CDCl_3$) 167.55, 160.58, 141.69, 121.82, 121.75, 116.51, 97.49, 97.36, 76.46, 63.34, 60.92, 59.96, 57.16, 50.74, 45.03, 40.74, 39.06, 37.97, 37.72, 37.33, 32.68, 32.31, 31.80, 30.18, 28.48, 26.01, 25.53, 21.21, 20.54, 19.92, 14.87, 13.46; IR (neat) 2938, 2904, 1710, 1634, 1438, 1378, 1213, 1200, 1144, 1113, 1075, 1057, 1025, 990, 974, 910, 867, 809, 729 cm^{-1}; HRMS *m/z* calculated for $C_{30}H_{47}O_4$ $[M+H]^+$ 471.3469 found 471.3468; and the pure, white solid (**15**) (151 mg, 5%) ^{1}H NMR (400 MHz, $CDCl_3$) δ 5.70 (1H, s, *H22*), 5.27 (1H, t, *J* = 5.2, 10.8 Hz, *H6*), 4.64 (1H, d, J = 4.0, *THP*), 4.04 (2H, dq, J = 1.2, 6.8, 14.0, 21.2 Hz, $O\underline{CH_2}CH_3$), 3.85 (2H, m, *THP*), 3.43 (2H, m, *THP*, *H3*), 2.28-2.05 (2H, m, ster.), 1.93-1.08 (30H, m, ster., 1.81 (3H, d' J = 0.8 Hz, *H21*), 1.18 (3H, t, J = 7.2, 14.4 Hz, $OCH_2\underline{CH_3}$)), 0.92 (3H, s, *H19*), 0.61 (3H, s, *H18*); ^{13}C NMR (101 MHz, $CDCl_3$) δ 166.91, 141.39, 141.22, 121.67, 121.60, 119.11, 97.28 97.12, 76.20, 63.16, 63.11, 59.57, 56.10, 50.45, 50.01, 47.22, 40.46, 36.86, 32.16, 31.50, 28.19, 25.70, 25.15, 24.85, 24.20, 20.93, 20.30, 19.61, 14.53, 14.36; IR (neat) 2937, 2909, 2875, 2825, 1711, 1627, 1463, 1450, 1439, 1427, 1376, 1200, 1152, 1112, 1094, 1077, 1053, 1024, 974, 865, 800, 730cm^{-1}; HRMS *m/z* calculated for $C_{25}H_{37}O_4$ $[M\text{-}OTHP]^+$ 369.2788 found 369.2795; and fraction containing both isomers (1.43 g, 51%).

3β-tetrahydropyranyloxy-24-norchol-5,20(22-trans)-dien-23-ol (14)

To a stirring solution of **13** (404 mg, 0.86 mmol) in dry THF (5 mL) was added lithium aluminium hydride (294 mg, 7.7 mmol) in THF (2 mL) dropwise over 10 minutes at rt. After 3.5 hours, the reaction was quenched by the addition of aqueous sodium bicarbonate (2 mL). The reaction mixture was filtered, extracted with EtOAc and washed three times with saturated sodium chloride. The organic layer was died over $MgSO_4$ and concentrated under reduced pressure. The residue was purified by flash column chromatography (hexane-EtOAc, 4:1) to afford the white solid (**14**) (297 mg, 81%). ^{1}H NMR (400 MHz, $CDCl_3$) δ 5.41 (1H, t, J = 6.4, 13.2 Hz, *H22*), 5.31 (1H, t, J = 6.0, 11.2 Hz, *H6*), 4.69 (1H, s, *THP*), 4.19 (2H, sept., J = 6.8, 12.4, 19.2, 38.0 Hz, *H23*), 3.88 (1H, m, *THP*), 3.47 (2H, m, *THP*, *H3*), 2.33-2.13 (2H, m, ster., 2.16 (1H, t, J = 13.2, 26.4 Hz, *H17*)), 2.03-1.37 (24H, m, ster., 1.66 (3H, s, *H21*)), 1.24-1.01 (4H, m, ster.), 0.97 (3H, s, *H19*), 0.53 (3H, s, *H18*); ^{13}C NMR (100MHz, $CDCl_3$) δ 141.6, 139.34, 124.89, 121.95, 97.46, 97.26, 76.45, 63.39, 60.08, 59.19, 56.86, 50.85, 44.03, 40.72, 39.15, 37.95, 37.34, 32.65, 32.33, 31.77, 30.16, 28.45, 25.97, 25.21, 24.74, 21.51, 20.56, 19.91, 18.60, 13.38; IR (neat) 3396, 2935, 2903, 1457, 1301, 1134, 1113, 1057, 1021, 991, 751, 735, 624, 604 cm^{-1}; HRMS *m/z* calculated for $C_{26}H_{42}O_3$ $[M\text{-}OH]^+$ 411.3258, found 411.3283.

24-norchol-5,20(22-trans)-dien-3β-ol-23-propargyl ether (2)

The reaction was performed under anhydrous conditions. To a stirring solution of sodium hydride 60% dispersion in mineral oil (94 mg, 2.34 mmol) in dry THF (1.0 mL) at 0 °C under nitrogen was added a mixture of **14** (100 mg, 0.23 mol) and 18-crown-6 (618 mg, 2.35 mol) dissolved in dry THF (5 mL). The reaction was stirred for 30 minutes at 0 °C then propargyl bromide (63 µL, 0.7 mmol) was added. The reaction mixture was warmed to 50 °C and stirred for 17 hours, then cooled to rt, extracted with EtOAc and washed twice with water and twice with saturated sodium chloride. The organic layer was died over $MgSO_4$ and concentrated under reduced pressure. The residue obtained (83.6 mg) was used without purification for the next reaction. The residue was dissolved in dry DCM/methanol

1:1 mixture (3 mL) and a solution of para-toluenesulphonic acid monohydrate (14.7 mg, 0.07 mol) dissolved in dry methanol (1 mL) was added at rt. The reaction was stirred overnight, then extracted with DCM and washed three times with water. The organic layer was died over $MgSO_4$ and concentrated under reduced pressure. The residue was purified by flash column chromatography (hexane-EtOAc, 4:1) to afford the white solid (**2**) (81 mg, 91%). ^{1}H NMR (400 MHz, $CDCl_3$) δ 5.32 (2H, m, *H6, H22*), 4.12 (4H, m, *H23*, O<u>CH₂</u>CCH), 3.50 (1H, m, *H3*), 2.40 (1H, t, J = 2.4 Hz, OCH_2C<u>CH</u>) 2.30-2.16 (2H, m, ster.), 2.06-1.91(2H, m, ster., 2.03 (1H, t, J = 10.8, 18.4 Hz, *H17*)), 1.84-1.75 (4H, m, ster.), 1.68 (3H, s *H21*), 1.65-1.38 (8H, m, ster.), 1.23-1.05 (4H, m, ster.), 0.98 (3H, s, *H19*), 0.94-0.81 (1H, m, ster.), 0.55 (3H, s, *H18*); ^{13}C NMR (101 MHz, $CDCl_3$) δ 141.00, 140.89, 121.81, 121.07, 80.28, 74.36, 71.97, 66.51, 59.03, 57.03, 56.56, 50.48, 43.77, 42.48, 38.87, 37.46, 36.76, 32.37, 32.00, 31.84, 24.93, 24.46, 21.26, 19.63, 18.52, 13.14; IR (neat) 3425, 3298, 2934, 2902, 2890, 2848, 1440, 1376, 1266, 1105, 1069, 1042, 1022, 953, 801, 735, 702, 660, 604 cm^{-1}; HRMS *m/z* calculated for $C_{26}H_{37}O$ $[M\text{-}HO]^+$ 365.2839, found 365.2844.

The NOESY spectrum shows no NOE interaction of proton *H17* (2.01 ppm) and protons of *H23* (4.09 ppm). There is also no NOE interaction found between protons *H22* (5.32 ppm) and *H21* (1.69 ppm), however their scalar coupling is found in the COSY spectrum. Further, NOE interaction as well as scalar coupling was found between protons *H23* (4.09 ppm) and *H21* (1.69 ppm). All these observations have confirmed the *trans* conformation of **2**.

2.3 Synthesis of 24-norchol-5,20(22-cis)-dien-3β-ol-23-propargyl ether (3)

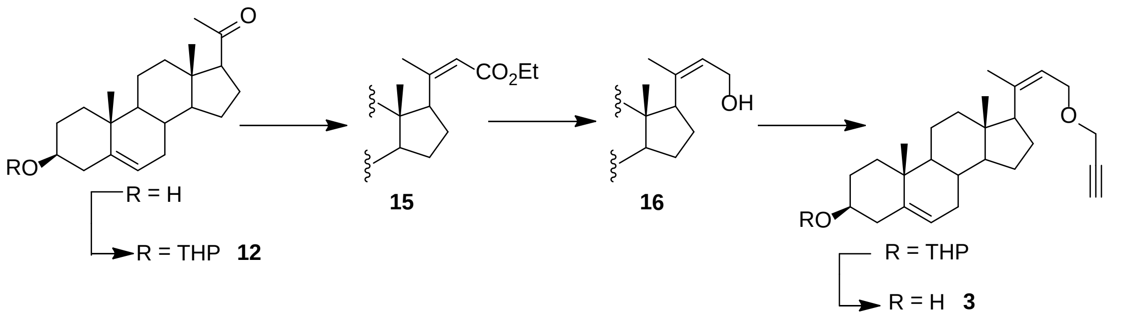

3β-tetrahydropyranyloxy-24-norchol-5,20(22-cis)-dien-23-ol (16)

To a stirring solution of **15** (129 mg, 0.27 mmol) in dry THF (3 mL) was added lithium aluminium hydride (53 mg, 1.4 mmol) in THF (4 mL) dropwise over 10 minutes at rt. After 3 hours 45 minutes, the reaction was quenched by the addition of aqueous sodium bicarbonate (2 mL). The reaction mixture was filtered, extracted with EtOAc and washed three times with saturated sodium chloride. The organic layer was died over $MgSO_4$ and concentrated under reduced pressure. The residue was purified by flash column chromatography (hexane-EtOAc, 4:1) to afford the white solid (**16**) (84 mg, 71%). ^{1}H NMR (400 MHz, $CDCl_3$) δ 5.54 (1H, t, J = 6.4, 13.2 Hz, *H22*), 5.33 (1H, s, *H6*), 4.69 (1H, d, J = 4.8 *THP*), 4.29-4.00 & 3.72-3.58 (2H, m, *H23*), 3.88 (1H, m, *THP*), 3.44 (2H, m, *THP*, *H3*), 2.48-2.14 (2H, m, ster., 2.46 (1H, t, J = 9.6, 19.2, *H17*)), 2.00-1.76 (5H, m, ster.), 1.74 (3H, s, *H21*), 1.72-1.37 (13H, m, ster.), 1.27-1.00 (5H, m, ster.), 0.98 (3H, s, *H19*), 0.94-0.78 (2H, m, ster.), 0.52 (3H, s, *H18*); IR (neat) 3393, 2939, 2904, 2869, 2851, 1452, 1438, 1377, 1352, 1135, 1116, 1036, 1023, 973, 954, 869 cm^{-1}; HRMS *m/z* calculated for $C_{26}H_{42}O_3$ $[M\text{-}OH]^+$ 411.3258, found 411.3267.

24-norchol-5,20(22-cis)-dien-3β-ol-23-propargyl ether (3)

The reaction was performed under anhydrous conditions. To a stirring solution of sodium hydride 60% dispersion in mineral oil (67 mg, 1.7 mmol) in dry THF (1.0mL) at 0 °C under nitrogen was added a mixture of **16** (75 mg, 0.17 mol) and 18-crown-6 (518 mg, 1.9 mol) dissolved in dry THF (5 mL. The reaction was stirred for 30 minutes at 0 °C then propargyl bromide (46.5 µL, 0.5 mmol) was added. The reaction mixture was warmed to 50 °C and stirred for 17 hours, then cooled to rt, extracted with EtOAc and washed twice with water and twice with saturated sodium chloride. The organic layer was died over $MgSO_4$ and concentrated under reduced pressure. The residue obtained (61 mg), was used without purification for the next reaction. The residue was dissolved in dry DCM/methanol 1:1 mixture (3 mL) and a solution of para-toluenesulphonic acid monohydrate (5.2 mg, 0.03 mol) dissolved in dry methanol (1 mL) was added at rt. The reaction was stirred overnight, then extracted with DCM and washed three times with water. The organic layer was died over $MgSO_4$ and concentrated under reduced pressure. The residue was purified by flash column chromatography (hexane-EtOAc, 4:1) to afford the yellow oil (**3**) (24 mg, 36%). ^{1}H NMR (400 MHz, $CDCl_3$) δ 5.47 (1H, t, J= 5.6 Hz, *H22*), 5.33 (1H, d, J = 4.4 Hz, *H6*), 4.24-3.90 (4H, m, *H23*, O$\underline{CH_2}$CCH), 3.50 (1H, m, *H3*, ster.), 2.47 (1H, t, J = 9.6 Hz, *H17*), 2.40 (1H, t, J = 2.4 Hz, $OCH_2C\underline{CH}$)), 2.28-2.17 (2H, m, ster.), 2.00-1.80 (5H, m, ster.), 1.75 (3H, s, *H21*), 1.68-1.33 (8H, m, ster.), 1.27-1.01 (4H, m, ster.), 0.98 (3H, s, *H19*), 0.94-0.77 (1H, m, ster.), 0.62 (3H, s, *H18*); ^{13}C NMR (101 MHz, $CDCl_3$) δ 141.04, 140.67, 124.57, 121.78, 80.27, 74.37, 71.95, 66.23, 57.19, 56.00, 51.56, 50.62, 46.03, 42.42, 37.98, 37.48, 36.82, 32.13, 31.79, 25.01, 24.75, 23.44, 21.25, 20.94, 19.61, 14.06; IR (neat) 3413, 3309, 2936, 2903, 2871, 2850, 2248, 1453, 1437, 1377, 1101, 1056, 1022, 954, 908, 728, 646 cm^{-1}; HRMS *m/z* calculated for $C_{26}H_{37}O$ $[M\text{-}OH]^+$ 365.2839, found 365.2846.

The NOESY spectrum shows a NOE interaction of proton *H17* (2.48 ppm) and protons of *H23* (4.23 ppm). There is both NOE interaction in the NOESY and scalar coupling in the COSY spectrum found between protons *H22* (5.47 ppm) and *H2*1 (1.75 ppm). However, there is no NOE interaction only scalar coupling found between protons *H23* (4.21-3.90 ppm) and *H21* (1.75 ppm). All these observations have confirmed the *cis* conformation of **3**.

2.4 Synthesis of Chol-5-en-3β-ol-23-propargyl ether (4)

The synthetic rout for compounds **18**, **19** and **20** uses the method reported by Schteingart and Hohmann,[9] and recently followed by Sepe *et al.*.[10] This method was also used for synthesis of compounds **21** and **22**.

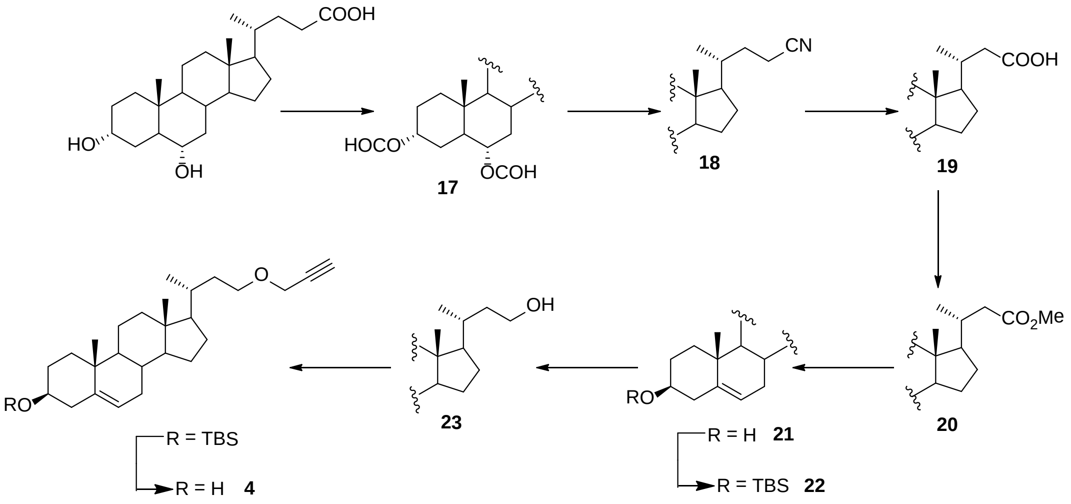

3,6-diformyloxy-5-cholan-24-oic acid (17)

Hyodeoxycholic acid (3.2 g, 8.2 mmol) was dissolved in 90 % formic acid (13.2 mL) and 70 % perchloric acid (33 µL) and heated at 50 °C for 1.5 h. The temperature was lowered to 40 °C over 25 minutes, before addition of acetic anhydride (10.5 mL) over 10 min. The reaction mixture was stirred for further 10 min. The solution was cooled to rt and poured into water (20 mL) with vigorous stirring. The precipitate was filtered, washed with water (30 mL), and dissolved in Et_2O. The solution was dried over $MgSO_4$ and concentrated under reduced pressure to afford white solid (**17**) (3.5 g, 95%). ^{1}H NMR (400 MHz, $CDCl_3$) δ 8.02 (1H, d, J = 0.4, CO<u>H</u>), 7.99 (1H, d, J = 0.8, CO<u>H</u>), 5.28 (1H; dt, *J* = 2.8, 9.6, 21.6 Hz, *H6*), 4.81 (1H; sept., J = 4.4, 10.8, 15.6, 31.2, *H3*), 2.37 – 2.20 (2H, m, ster.), 2.03 – 1.03 (25H; m, ster.), 0.97 (3H, s, *19H*), 0.90 (3H; d, J = 6.4 Hz; *H21*) , 0.63 (3H; s; *H18*); ^{13}C NMR (101 MHz, $CDCl_3$) δ 179.81, 160.85, 160.77, 73.75, 71.11, 56.21, 56.05, 45.56, 43.08, 40.02, 36.33, 35.46, 35.12, 34.82, 31.42, 31.04, 30.87, 28.26, 26.59, 26.32, 24.26, 23.44, 20.86, 18.43, 12.22; IR (neat) 2940, 2872, 2343, 1719, 1458, 1381, 1176, 985, 960, 913, 734, 682, 662, 651, 627, 620, 611 cm^{-1}; HRMS m/z calculated for $C_{25}H_{39}O_4$ $[M\text{-}OCOH]^+$ 403.2843, found 403.2848.

3,6-diformyloxy-24-nor-5-cholane-23-nitrile (18)

A mixture of **17** (3.5 g, 7.8 mmol) in trifluoroacetic acid (12.3 mL) and trifluoroacetic anhydride (3.3 mL, 23.4 mmol) was stirred in an ice-bath until dissolution occurred. Sodium nitrite (653 mg, 9.4 mmol) was then added in small portions over 1.5 hours, and the reaction mixture was stirred at 5 °C for 1 h, then at 40 °C for 3 h. The solution was allowed to cool to room temperature and poured into 2 M NaOH solution mixed with ice (25 mL). Enough EtOAc was added to dissolve a formed precipitate and the mixture was transferred to the

separating funnel. The organic layer was washed three times with 1M NaOH. The solution was dried over Na_2SO_4 and concentrated under reduced pressure. The residue was purified by flash column chromatography (DCM-methanol, 9:1) to afford the white solid (**18**) (2.4 g, 75%). ^{1}H NMR (400 MHz, $CDCl_3$) δ 8.02 (1H, d, J = 0.8, CO<u>H</u>), 7.99 (1H, d, J = 0.8, CO<u>H</u>), 5.28 (1H; quintet, *J* = 4.8, 10.4, 21.6 Hz, *H6*), 4.81 (1H; sept., J = 4.8, 11.2, 16.0, 32.0, *H3*), 2.35 – 2.19 (2H, m, ster.), 1.96 – 1.07 (26H; m, ster., 1.13 (3H, d, J = 6.4, *H21*)), 0.97 (3H, s, *19H*), 0.65 (3H; s; *H18*); ^{13}C NMR (101 MHz, $CDCl_3$) δ 161.11, 161.02, 119.37, 73.93, 71.25, 56.32, 55.28, 45.85, 43.44, 40.26, 39.93, 36.62, 35.42, 35.11, 33.99, 31.65, 28.54, 26.89, 26.63, 25.29, 24.49, 23.73, 21.10, 19.79, 12.60; IR (neat) 2942, 2870, 2243, 1714, 1464, 1455, 1381, 1169, 1119, 1089, 1047, 1026, 1016, 960, 929, 734, 703, 620 cm^{-1}; HRMS m/z calculated for $C_{25}H_{38}NO_4$ $[M+H]^+$ 416.2795, found 416.2823.

24-nor-hyodeoxycholic acid (19)

A solution of **18** (2.4 g, 5.8 mmol) in 10% KOH in water/EtOH (1:1, 100 mL) was refluxed for 40 h, solvents were evaporated and after addition of silica, the mixture was dry-loaded onto a flash chromatography column. The crude material was purified by flash chromatography (DCM-methanol, 9:1) to afford white solid (**19**) (1.5 g, 68%). ^{1}H NMR (400 MHz, MeOD) δ 3.94 (1H; dt, *J* = 4.4, 9.2, 11.6, 21.2 Hz, *H6*), 3.44 (1H; sept., J = 4.4, 10.8, 15.6, 30.8 Hz, *H3*), 2.36 – 2.09 (1H, m, ster.), 1.97-1.71 (6H, m, ster.), 1.63-0.91 (23H; m, ster., 0.93 (3H, d, J = 6.0, *H21*)), 0.86 (3H, s, *19H*), 0.66 (3H; d; J = 6.0, *H18*); ^{13}C NMR (101 MHz, MeOD) δ 179.63, 72.92, 69.17, 58.19, 57.97, 50.40, 44.69, 41.83, 37.49, 37.34, 36.74, 36.07, 35.78, 35.57, 31.68, 30.53, 29.84, 25.82, 24.66, 22.45, 20.54, 20.05, 13.02; IR (neat) 3355, 2936, 2868, 1707, 1660, 1423, 1414, 1378, 1315, 1299, 1258, 1202, 1081, 1040, 1031, 921 cm^{-1}; HRMS m/z calculated for $C_{23}H_{37}O_3$ $[M-OH]^+$ 361.2735 found 361.2742.

Methyl 3,6-dihydroxy-24-nor-5-cholan-23-oate (20)

Para-toluenesulphonic acid monohydrate (0.5 g, 2.8 mmol) was added to a solution of **19** (1.5 g, 4.0 mmol) in dry methanol (60 mL).The solution was stirred at rt for 30 hours, then neutralized with diluted sodium hydroxide to pH 7. MeOH was evaporated and the crude product was extracted with ethyl acetate and washed three times with water and once saturated sodium chloride. The organic layer was died over $MgSO_4$ and concentrated under reduced pressure. The residue was purified by flash column chromatography (DCM-methanol, 9:1) to afford the white solid (**20**) (0.9 g, 56%). ^{1}H NMR (400 MHz, $CDCl_3$) δ 4.03 (1H; dt, *J* = 4.8, 9.6, 21.6 Hz, *H6*), 3.63 (3H; s, *COO<u>Me</u>*), 3.59 (1H; m, *H3*), 2.42 – 2.38 (1H, m), 2.02–1.50 (12H; m, ster.), 1.43 – 0.99 (13H; m, ster.), 0.0.94(3H, d, J = 6.4, *H21*), 0.88 (3H; s, *H19*) , 0.65 (3H; s; *H18*); ^{13}C NMR (101 MHz, $CDCl_3$) δ 174.60, 72.08, 68.54, 56.64, 56.53, 51.94, 48.87, 43.43, 41.91, 40.31, 40.26, 36.45, 36.03, 35.44, 35.31, 34.30, 30.70, 29.68, 28.72, 24.67, 23.99, 21.21, 20.01, 12.56; IR (neat) 3352, 2937, 2866, 1732, 1446, 1437, 1263, 1227, 1195, 1159, 1088, 1038, 1028, 734, 702, 657, 645, 627, 614 cm^{-1}; HRMS m/z calculated for $C_{24}H_{39}O_3$ $[M-OH]^+$ 375.2894, found 375.2921.

Methyl 3β-hydroxy-24-nor-5-chol-23-enoate (21)

To a stirring solution of **20** (711 mg, 1.8 mmol) in dry pyridine (6 mL) was added methanesulfonyl chloride (420 µL, 5.3 mmol) at rt. After 15 minutes the reaction mixture was taken up in EtOAc and washed once with 1.0 M HCl, water, and saturated sodium chloride. The organic layer was died over $MgSO_4$ and concentrated under reduced pressure. The crude material (896 mg) was dissolved in DMF (7.5 mL) and water (1.1 mL) and used without further purification. After addition of potassium acetate (130 mg, 1.3 mmol), the mixture was refluxed at 110 °C overnight, poured into water and extracted with EtOAc. The combined extracted where washed twice with saturated sodium chloride, dried over $MgSO_4$ and concentrated under reduced pressure. The residue was purified by flash column chromatography (hexane-EtOAc, 4:1) to afford the white solid (**21**) (242 mg, 36%). ^{1}H NMR (400 MHz, $CDCl_3$) δ 5.32 (1H, q, J = 2.0, 3.2, 5.2 Hz, *H6*), 3.64 (3H, s, *COO<u>Me</u>*, 3.48 (1H, m, *H3*), 2.43-2.15 (3H, m, ster.), 2.00-1.75 (7H, m, ster.), 1.62-1.41 (8H, m, ster.), 1.34-1.00 (7H, m, ster.), 0.99 (3H, s, *H19*), 0.96 (3H, d, J = 6.0, *H21*), 0.94-0.87 (1H, m, ster.), 0.70 (3H, s, *H18*); ^{13}C NMR (101 MHz, $CDCl_3$) δ 173.95, 140.67, 121.49, 71.66, 56.62, 55.80, 51.26, 49.92, 42.34, 42.17, 41.34, 39.51, 37.13, 36.37, 33.69, 31.77, 31.73, 31.53, 28.09, 24.12, 20.92, 19.44, 19.28, 11.78.; IR (neat) 3378, 2937, 2905, 2889, 2851, 1734, 1437, 1377, 1245, 1227, 1196, 1165, 1154, 1048, 1023, 1007, 639, 633, 624, 608 cm^{-1}; HRMS m/z calculated for $C_{24}H_{37}O_2$ $[M-OH]^+$ 357.2788, found 357.2805.

Methyl 3β-tert-butyldimethylsilyloxy-5-cholen-23-oate (22)

Tert-butyldimethylsilyl chloride (282 mg, 1.9 mmol) was added to a solution of **21** (231 mg, 0.61 mmol) and DIPEA (200 µg, 0.002 mmol) in dry DMF (5 mL) at room temperature. After 17 hours, the reaction mixture was extracted with EtOAc and washed three times with saturated sodium chloride. The organic layer was died over Na_2SO_4 and concentrated under reduced pressure. The residue was purified by flash column chromatography (hexane-EtOAc, 7:3) to afford the white solid (**22**) (110 mg, 37%). ^{1}H NMR (400 MHz, $CDCl_3$) δ 5.29 (1H, d, J = 4.0 *H6*), 3.64 (3H, s, *COO<u>Me</u>*), 3.47 (1H, sept, J = 4.8, 10.8, 15.6, 31.2 Hz, *H3*), 2.42-2.11 (3H, m, ster.), 2.00-1.67 (7H, m, ster.), 1.61-1.01 (13H, m, ster.), 0.96 (6H, d, J = 7.6, *H19, H21*), 0.86 (9H, s, (t-Bu)-Si) 0.69 (3H, s, *H18*), 0.04 (6H, s, $(CH_3)_2Si$). ^{13}C NMR (101 MHz, $CDCl_3$) δ 173.95, 141.49, 120.96, 72.52, 56.67, 55.82, 51.26, 50.03, 42.71, 42.37, 41.36, 39.55, 37.28, 36.47, 33.70, 31.97, 31.79, 28.11, 25.84, 24.13, 20.92, 19.45, 19.32, 18.17, 11.78, -4.68; IR (neat) 2958, 2934, 2894, 2881, 2862, 1738, 1472, 1432, 1275, 1256, 1226, 1194, 1095, 1018, 1005, 890, 869, 837, 802, 775 cm^{-1}; HRMS m/z calculated for $C_{24}H_{37}O_2$ $[M-OTBS]^+$ 357.2788, found 357.2792.

3-β-tetrahyropyranyloxy-chol-5-en-24-ol (23)

A solution of lithium aluminium hydride (65 mg, 1.7 mmol) in dry THF (2 mL) was added dropwise over 10 minutes to a solution of **22** (105 mg, 0.21 mmol) in dry THF (2 mL). After 4 hours the reaction was quenched with saturated sodium bicarbonate, filtered and extracted with EtOAc. The organic layer was washed with brine, dried over $MgSO_4$ and concentrated under reduced pressure. The residue was purified by flash column chromatography (hexane-EtOAc, 4:1) to afford the white solid **23** (79 mg, 80%). ^{1}H NMR (400 MHz, $CDCl_3$) δ 5.29

(1H, q, J = 2.0, 3.2, 5.2 Hz, *H6*), 3.47 (1H, sept, J =4.8, 10.8, 15.6, 31.3 Hz, *H23*), 3.47, 2.28-2.12 (2H, m, ster.), 2.01-1.67 (6H, m, ster.), 1.61-0.93 (23H, m, ster., 0.98 (3H, s, *H19*), 0.93 (3H, d, J = 6.4, *H21*)), 0.87 (9H, s, (t-Bu)-Si) 0.67 (3H, s, *H18*), 0.03 (6H, s, $(CH_3)_2Si$); ^{13}C NMR (100MHz, $CDCl_3$) δ ^{13}C NMR (101 MHz, $CDCl_3$) δ^{13}C NMR (101 MHz, $CDCl_3$) δ 141.80, 121.34, 121.34, 72.86, 61.15, 57.02, 56.57, 50.39, 43.04, 42.65, 40.02, 39.23, 37.60, 36.80, 33.15, 32.30, 32.13, 28.57, 26.16, 24.49, 21.27, 19.65, 19.09, 12.04, -4.37; IR (neat) 3279, 2959, 2935, 2896, 2881, 2854, 1471, 1460, 1383, 1255, 1085, 1035, 1005, 959, 888, 870, 838, 729 cm^{-1}; HRMS m/z calculated for $C_{23}H_{37}O$ $[M-OH]^+$ 329.2839, found 329.2855.

Chol-5-en-3β-ol-23-propargyl ether (4)

The reaction was performed under anhydrous conditions. To a stirring solution of sodium hydride 60% dispersion in mineral oil (65 mg, 1.6 mmol) in dry THF (1.0 mL) at 0 °C under nitrogen was added a mixture of **23** (75 mg, 0.16 mol) and 18-crown-6 (430 mg, 1.6 mol) dissolved in dry THF (5 mL). The reaction was stirred for 30 minutes at 0 °C then propargyl bromide (43 µL, 0.5 mmol) was added. The reaction mixture was heated to 50 °C for 17 hours, and then cooled to rt. The mixture was extracted with EtOAc and washed twice with water and twice with saturated sodium chloride. The organic layer was died over $MgSO_4$ and concentrated under reduced pressure. The residue obtained (85 mg), was used without purification for the next reaction, by dissolving in dry THF (3 mL) containing 1.0 M TBAF in THF (0.5 mL, 0.5 mmol). The reaction was stirred overnight at rt, then extracted with EtOAc and washed three times with water. The organic layer was died over $MgSO_4$ and concentrated under reduced pressure. The residue was purified by flash column chromatography (hexane-EtOAc, 4:1) to afford the white solid (**4**) (43 mg, 68%); ^1H NMR (400 MHz, $CDCl_3$) δ 5.31 (1H, *H6*), 4.09 (2H, d, J= 2.4 Hz, O$\underline{CH_2}$CCH), 3.50 (3H, m, *H23*, *H3*), 2.39 (1H, t, J = 2.4, C$\underline{CH}$) 2.29-2.16 (2H, m, ster.), 2.07-1.87 (3H, m, ster.), 1.84-1.70 (4H, m, ster.), 1.69-1.30 (8H, m, ster.), 1.27-0.99 (7H, m, ster.), 0.97 (3H, s, *H19*), 0.91 (3H, d, J = 6.4, *H21*), 0.65 (3H, s, *H18*); ^{13}C NMR (101 MHz, $CDCl_3$) δ 140.93, 121.84, 80.24, 74.31, 71.94, 68.48, 58.18, 56.92, 56.55, 56.16, 50.27, 42.59, 42.42, 39.95, 37.42, 36.67, 35.67, 33.39, 32.05, 31.78, 28.42, 24.45, 21.25, 19.57, 18.99, 12.02; IR (neat) 3414, 3273, 2963, 2935, 2900, 2888, 2861, 2851, 1442, 1377, 1353, 1098, 1062, 1054, 1022, 1010, 955, 801, 658, 643, 626, 614 cm^{-1}; HRMS *m/z* calculated for $C_{26}H_{39}O$ $[M-HO]^+$ 367.2995, found 367.3008.

2.5 Synthesis of 3β-hydroxychol-5-en-23-yne (5)

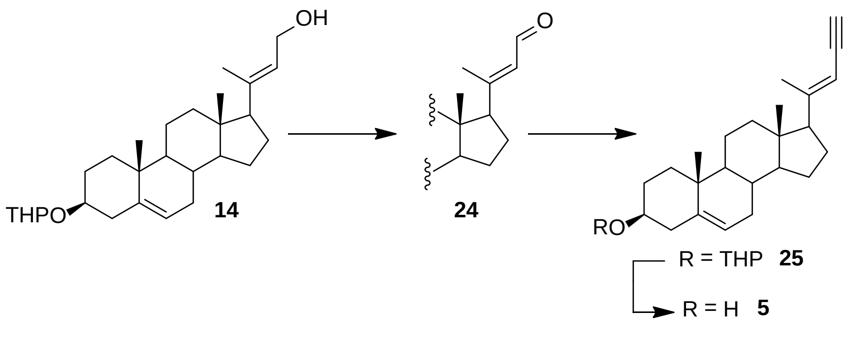

3β-tetrahydropyranyloxy-24-norchol-5,20(22-trans)-dien-23-al (24)

A solution of **14** (198 mg, 0.46 mmol) dissolved in DCM (3 mL) was cooled to 0 ˚C. Dess-Martin periodinane (297 mg, 0.7 mmol) in DCM (3 mL) was added and the solution stirred for 2 hours at 0 ˚C. The reaction was quenched by the addition of aqueous sodium bicarbonate (2 mL), extracted with DCM and washed three times with water. The organic layer was died over $MgSO_4$ and concentrated under reduced pressure. The residue was purified by flash column chromatography (hexane-EtOAc, 9:1) to afford the white solid (**24**) (105 mg, 53%). ^{1}H NMR (400 MHz, $CDCl_3$) δ 10.06 (1H, d, J = 8.0 Hz, CO<u>H</u>) 5.90 (1H, d, J = 8.0 Hz, *H22*), 5.32 (1H, t, J = 5.6, 11.2 Hz, *H6*), 4.69 (1H, d, J = 4.4 Hz, *THP*), 3.89 (1H, m, *THP*), 3.47 (2H, m, *THP*, *H3*), 2.35-2.30 (1H, m, ster.), 2.23-2.14 (4H, t, J = 9.2 Hz, *H17*), 2.17 (3H, d, J = 0.8 Hz, *H21*)), 2.01-1.67 (9H, m, ster.), 1.63-1.37 (10H, m, ster.), 1.30-1.01 (4H, m, ster.), 0.98 (3H, s, *H19*), 0.96-0.92 (1H, m, ster.), 0.59 (3H, s, *H18*); ^{13}C NMR (101 MHz, $CDCl_3$) δ 191.86, 164.77, 141.66, 128.43, 121.77, 121.69, 97.49, 76.38, 63.43, 60.68, 57.34, 50.71, 45.63, 40.72, 39.11, 37.96, 37.71, 37.31, 32.67, 32.28, 31.78, 30.16, 28.45, 26.00, 25.34, 24.87, 21.52, 20.59, 19.92, 13.63.; IR (neat) 2937, 2904, 2971, 2848, 1669, 1452, 1439, 1377, 1365, 1197, 1135, 1112, 1056, 1037, 1024, 975, 896, 868 cm^{-1}; HRMS *m/z* calculated for $C_{23}H_{33}O$ [M-OTHP]$^+$ 325.2526, found 325.2540.

3β-tetrahydropyranyloxy-24-norchol-5,20(22-trans)-dien-23-yne (25)

To a stirring solution of diisopropylamine (35 µL, 0.25 mmol) in dry THF (2 mL) was added 1.2 M n-buthyllithium in hexane (280 µL, 0.34 mmol) at −78 °C. The reaction was stirred for 30 minutes at −78 °C, prior to addition of 2.0 M trimethylsilyldiazomethane in hexane (150 µL, 0.3 mmol). After stirring at −78 °C for 30 minutes, a solution of **24** (90 mg, 0.21 mmol) in dry THF (2 mL) was added and left for 1 hour, before being allowed to warm to rt over 2 hours. The reaction was quenched by pouring over ice-water and extracted with Et_2O. The organic layer was died over $MgSO_4$ and concentrated under reduced pressure. The residue was purified by flash column chromatography (hexane-EtOAc, 95:5) to afford the white solid (**25**) (43 mg; 48%). ^{1}H NMR (400 MHz, $CDCl_3$) δ 5.33 (2H, m, *H6, H22*), 4.69 (1H, d, J = 4.4 Hz, *THP*), 3.90 (1H, m, *THP*), 3.49 (2H, m, *THP*, *H3*), 3.08 (1H, d, J = 2.0 Hz, C<u>CH</u>) 2.36-2.31 (1H, m, ster.), 2.21-2.00 (2H, m, ster, 2.19 (1H, t, J = 13.2, 25.2, *H17*)), 1.95 (3H, 2, *H21*), 1.90-1.37 (18H, m, ster.), 1.22-1.02 (4H, m, ster.), 1.00 (3H, s, *H19*), 0.98-0.91 (1H, m, ster.), 0.57 (3H, s, *H18*); ^{13}C NMR (101 MHz, $CDCl_3$) δ 154.68, 141.16, 121.58, 104.55, 97.18, 97.03, 82.35, 80.76, 76.11, 63.15, 58.56, 56.64, 50.47, 44.44, 40.42, 38.93, 38.63, 37.64, 37.39, 32.33, 32.00, 31.48, 29.86, 28.14, 25.68, 25.17, 24.46, 21.36, 20.30, 19.62, 13.10; HRMS *m/z* calculated for $C_{29}H_{43}O_2$ [M+H]$^+$ 432.3258, found 423.3260.

3β-hydroxychol-5-en-23-yne (5)

To a stirring solution of **25** (27 mg, 0.06 mmol) dissolved in dry DCM/methanol 1:1 mixture (1 mL) was added a solution of p-toluenesulphonic acid monohydrate (3 mg, 0.01 mol) dissolved in dry methanol (1 mL) at rt. The reaction was left stirring overnight, before being extracted with DCM and washed three times with water. The organic layer was died over $MgSO_4$ and concentrated under reduced pressure. The residue was purified by flash column

chromatography (hexane-EtOAc, 4:1) to afford the white solid (**5**) (17 mg, 77%). ^{1}H NMR (400 MHz, $CDCl_3$) δ 5.33 (1H, t, J = 2.0Hz, *H6)* 5.30 (1H, quin, J = 0.8, 2.0, 3.2, 4.0, *H22*), 3.51 (1H, m, *H3*), 3.08 (1H, d, J = 2.0 Hz, CCH) 2.28-2.09 (2H, m, ster., 2.11 (1H, t, J = 8.8, *H17*)), 1.94 (3H, s, *H21*), 1.85-1.75 (3H, m, ster.), 1.72-1.52 (7H, m, ster.), 1.50-1.39 (3H, m, ster.), 1.26-1.15 (4H, m, ster.), 1.12-1.02 (2H, m, ster.), 0.99 (3H, s, *H19*), 0.56 (3H, s, *H18*); ^{13}C NMR (101 MHz, $CDCl_3$) δ 154.60, 141.04, 121.71, 104.63, 82.35, 80.79, 71.96, 58.59, 56.68, 50.44, 44.45, 42.47, 38.67, 37.47, 36.76, 32.36, 31.98, 31.84, 25.20, 24.47, 21.33, 21.23, 19.63, 13.11; IR (neat) 3390, 3278, 2965, 2932, 2902, 2867, 2851, 1462, 1437, 1377, 1111, 1050, 1011, 1092, 954, 843, 836, 800, 636, 624 cm^{-1}; HRMS *m/z* calculated for $C_{24}H_{33}$ [M-OH^{+} 321.2577, found 321.2608.

2.6 Synthesis of 3β-hydroxychol-5-en-24-yne (6)

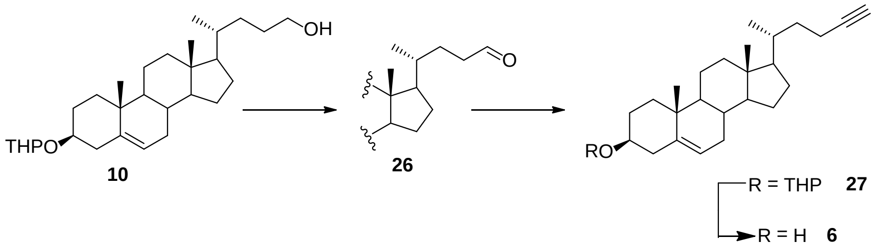

3β-(tetrahydro-2H-pyran-2-yloxy)chol-5-en-24-al (26)

A solution of **10** (198 mg, 0.45 mmol) dissolved in DCM (3 mL) was cooled to 0 ˚C. Dess-Martin periodinane (126 mg, 0.3 mmol) in DCM (3 mL) was added and the solution stirred for 2 hours at 0 ˚C. The reaction was quenched with sodium thiosulphate, diluted with distilled water and extracted three times with EtOAc. The combined organic extracts were dried over $Na_2SO_4$and concentrated *in vacuo* to yield the beige-coloured solid (**26**) (81 mg, 41%). ^{1}H NMR (400 MHz, $CDCl_3$) δ 9.74 (1H, t, J = 1.6, 3.2 Hz, CHO), 5.32 (1H, t, J = 6.0, 11.2 Hz, *H6*), 4.69 (1H, m, THP), 3.988 (1H, m, THP), 3.48 (2H, m, THP, *H3*), 2.46-2.15 (4H, m, ster.), 1.99-1.67 (8H, m, ster.), 1.61-1.38 (12H, m, ster.), 1.34-1.23 (3H, m, ster.) 1.18-1.00 (4H, m, ster.), 0.98 (3H, s, *H19*), 0.91(3H, d, J = 6.4 Hz, *H21*), 0.67 (3H, s, *H18*); ^{13}C NMR (101 MHz, $CDCl_3$) δ 203.48, 171.36, 169.98, 141.70, 141.00, 140.80, 134.04, 133.12, 131.74, 127.96, 121.55, 121.47, 96.93, 96.76, 94.57, 76.78, 76.04, 62.84, 62.74, 60.48, 56.67, 55.76, 50.10, 50.04, 49.06, 42.36, 40.90, 40.17, 39.69, 38.72, 37.41, 37.16, 36.75, 35.32, 31.85, 31.22, 30.73, 29.63, 28.18, 27.94, 25.45, 24.24, 21.08, 21.02, 19.99, 19.91, 19.38, 18.41, 14.19, 11.86;IR (neat) 2941, 2872, 1707, 1467, 1381, 1289, 1250, 1200, 1184, 1163, 1135, 1110, 1074, 1016, 912, 741, 667, 635 cm^{-1}; MS (ESI) obtained for $C_{24}H_{37}O$ [M-OTHP]$^{+}$ 341.3.

3β-(tetrahydro-2H-pyran-2-yloxy)chol-5-en-24-yne (27)

To a stirring solution of diisopropylamine (20 µL, 0.14 mmol) in dry THF (1 mL) was added 1.2 M n-butyllithium in hexane (150 µL, 0.20 mmol) at −78 °C. The reaction was stirred for 30 minutes at −78 °C, prior to addition of 2.0 M trimethylsilyldiazomethane in hexane (60 µL, 0.12 mmol). After stirring at −78 °C for 30 minutes a solution of **26** (35 mg, 0.08 mmol) in dry THF (3 mL) was added and the reaction stirred for 1 hour, before being

allowed to warm to rt over 2 hours. The reaction was quenched by pouring over ice-water and extracted with Et_2O. The organic layer was died over Na_2SO_4 and concentrated under reduced pressure. The residue was purified by flash column chromatography (hexane-EtOAc, 95:5) to afford the white solid (**27**) (13 mg; 37%). ^{1}H NMR (400 MHz, $CDCl_3$): δ 5.34 (1H, t, J = 5.6, 11.2 Hz, *H6*), 4.69 (1H, m, THP), 3.89 (1H, m, THP), 3.48 (2H, m, THP, *H3*), 2.34-2.03 (4H, m, ster.; 2.1 (1H, s, CCH)), 1.99-1.36 (20H, m, ster.), 1.31-1.01 (8H, m, ster.), 0.98 (3H, s, *H19*), 0.90 (3H, d, J = 6.4 Hz, *H21*), 0.66 (3H, s, *H18*); ^{13}C NMR (101 MHz, $CDCl_3$): δ 140.91, 121.49, 96.84, 85.22, 75.98, 67.90, 62.87, 56.72, 55.84, 50.10, 46.10, 45.74, 42.42, 40.24, 39.73, 38.76, 37.45, 37.19, 36.77, 34.81, 31.87, 31.27, 29.70, 28.17, 25.49, 24.27, 23.65, 21.04, 20.06, 19.40, 18.17, 15.48, 11.87; MS (ESI) obtained for $C_{25}H_{37}$ $[M\text{-}OTHP]^+$ 337.3.

3β-hydroxychol-5-en-24-yne (6)

To the stirring solution of **27** (10 mg, 0.02 mmol) dissolved in dry DCM/methanol 1:1 mixture (1 mL) was added a solution of para-toluenesulphonic acid monohydrate (1 mg, 0.005 mol) dissolved in dry methanol (1 mL) at rt. The reaction was stirred overnight, then extracted with DCM and washed three times with water. The organic layer was died over $MgSO_4$ and concentrated under reduced pressure. The residue was purified by flash column chromatography (hexane-EtOAc, 4:1) to afford the white solid (**6**) (3 mg, 38%). ^{1}H NMR (400 MHz, $CDCl_3$): δ 5.33 (1H, t, J = 2.4, 5.2 Hz, *H6*), 3.51 (1H, sept, J = 4.4, 10.8, 15.6, 31.2 Hz, *H3*), , 2.12-1.79 (8H, m, ster.; 1.93 (1H, d, J = 2.8 Hz, CCH)), 1.72-1.01 (18H, m, ster.), 0.99 (3H, s, *H19*), 0.94-0.82 (4H, m, ster.,0.90 (3H, d, J = 6.8 Hz, *H21*)), 0.68 (3H, s, *H18*); ^{13}C NMR (101 MHz, $CDCl_3$): δ 140.99, 121.90, 85.40, 72.02, 68.10, 56.97, 56.09, 50.32, 42.66, 42.52, 39.98, 37.47, 36.72, 35.38, 35.04, 32.11, 31.88, 29.93, 28.37, 24.49, 21.29, 19.62, 18.39, 15.71, 12.09; IR (neat): 3262, 3238, 2932, 2850, 1460, 1443, 1374, 1258, 1050, 1040, 953, 928, 838, 798 cm^{-1}; HRMS *m/z* calculated for $C_{25}H_{37}$ $[M\text{-}OH]^+$ 337.2890, found 337.2914.

^{1}H NMR Chol-5-en-3β-ol-24-propargyl ether (**1**)

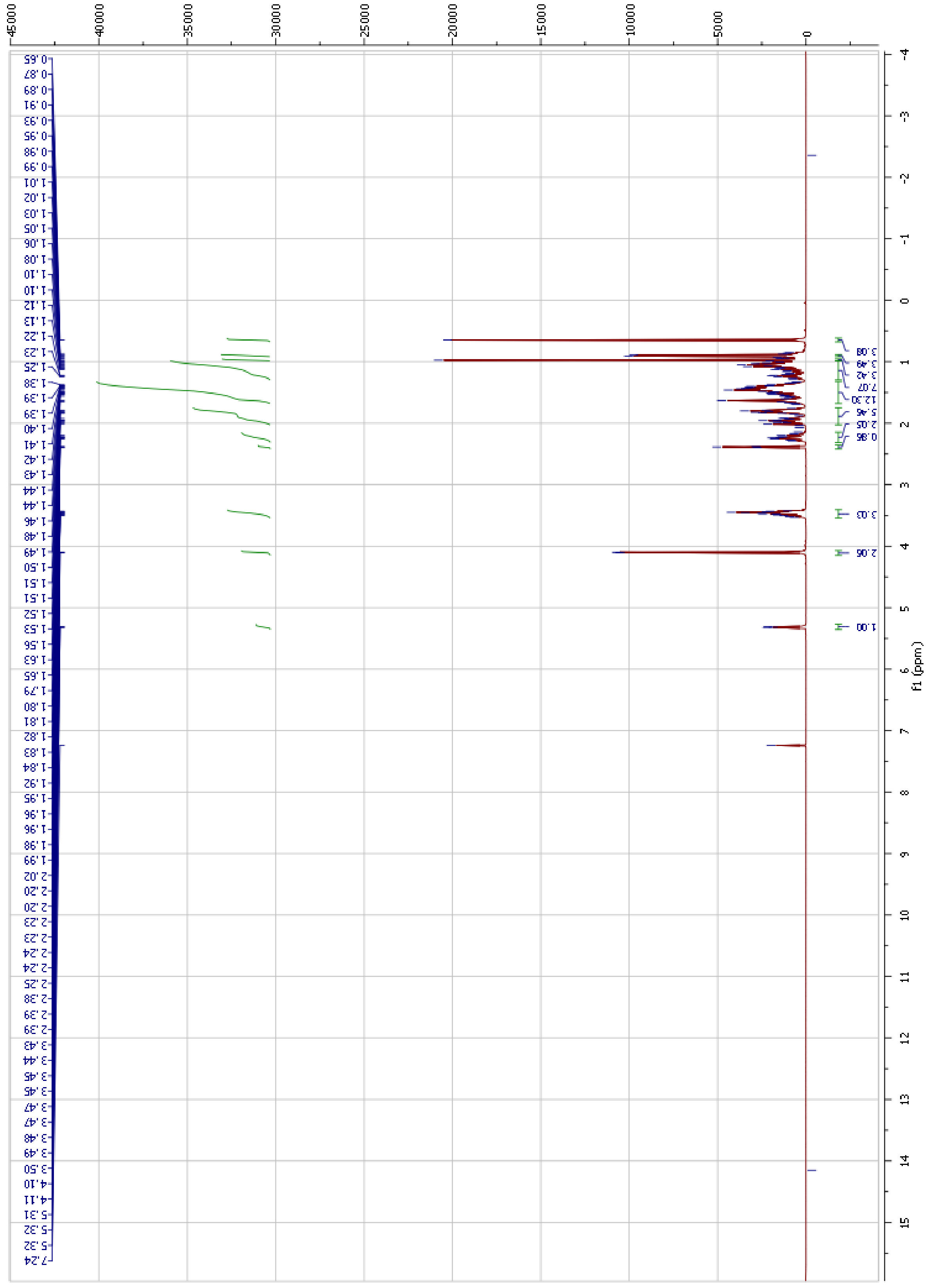

^{13}C NMR Chol-5-en-3β-ol-24-propargyl ether (**1**)

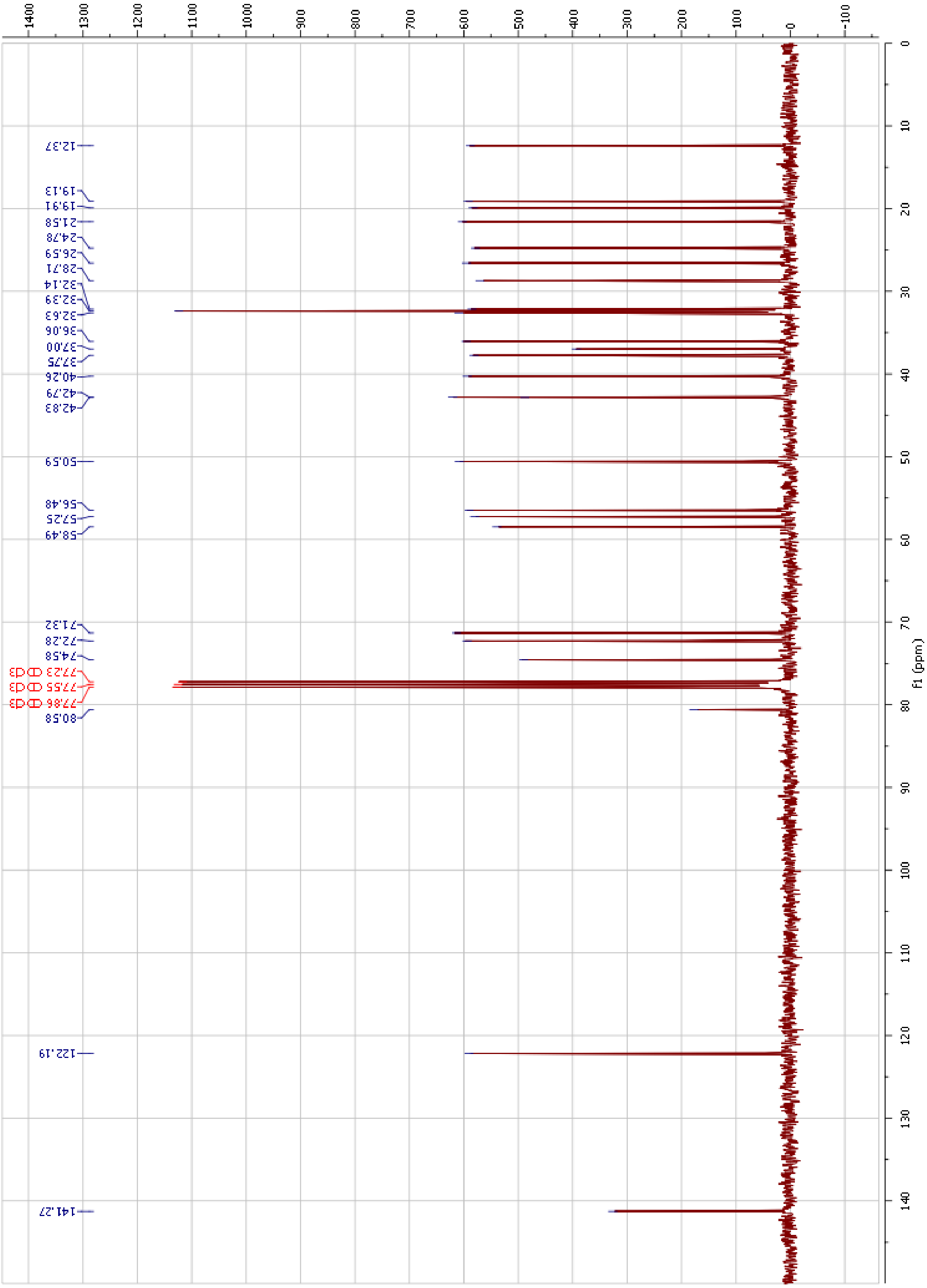

^{1}H NMR 24-norchol-5,20(22-trans)-dien-3β-ol-23-propargyl ether (**2**)

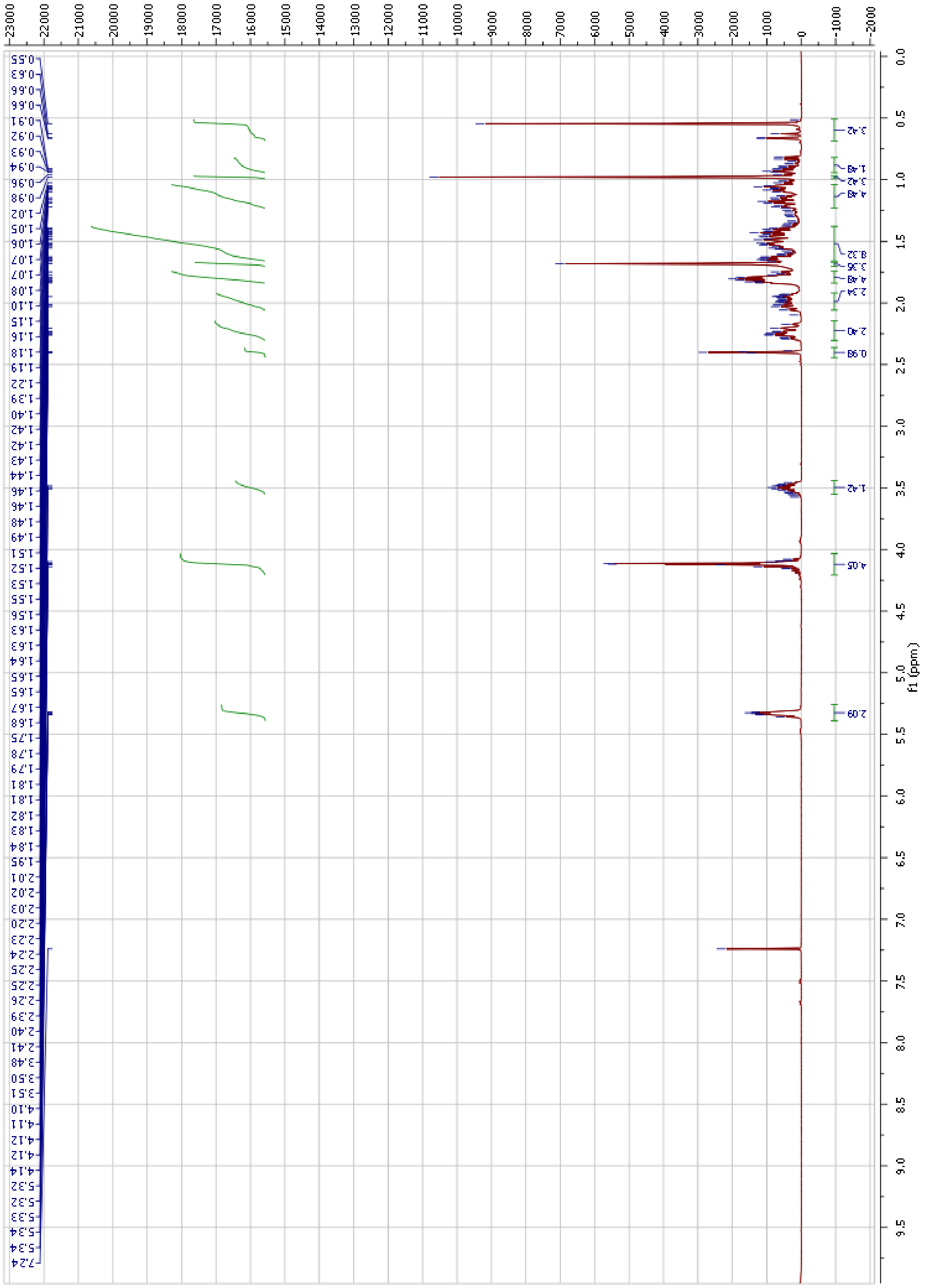

^{13}C NMR 24-norchol-5,20(22-trans)-dien-3β-ol-23-propargyl ether (**2**)

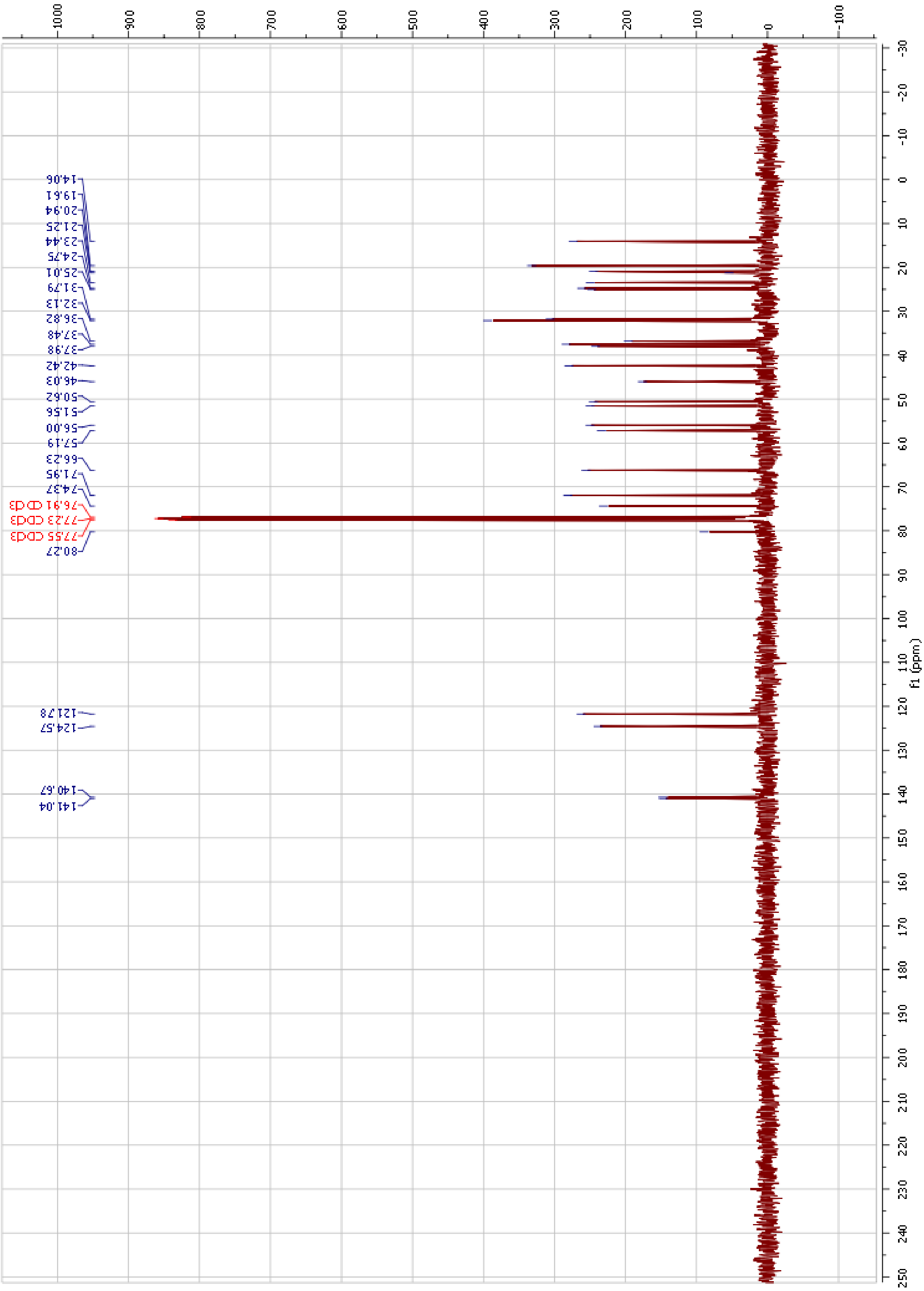

NOE NMR (black) and COSY (blue) 24-norchol-5,20(22-trans)-dien-3β-ol-23-propargyl ether (**2**)

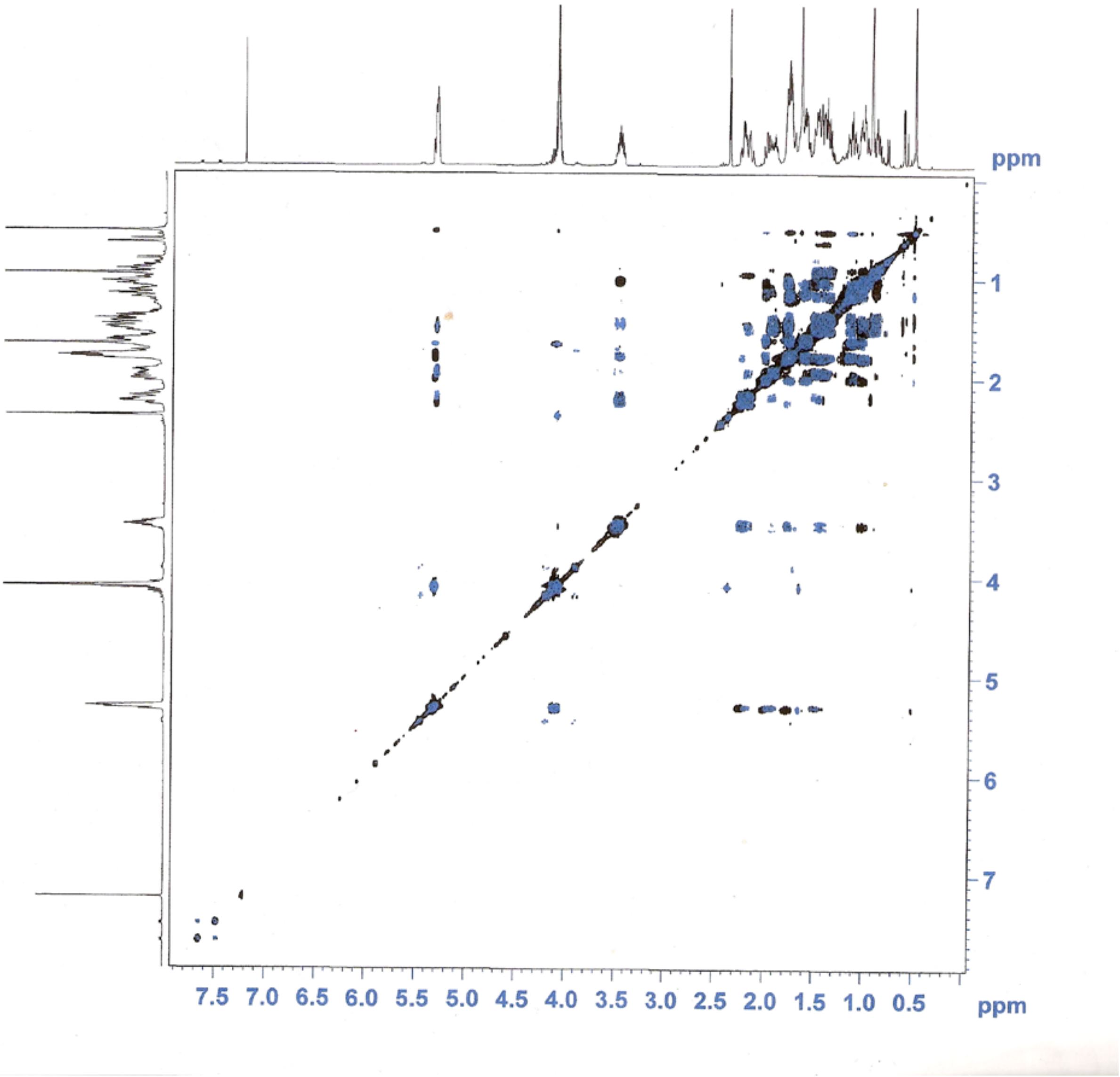

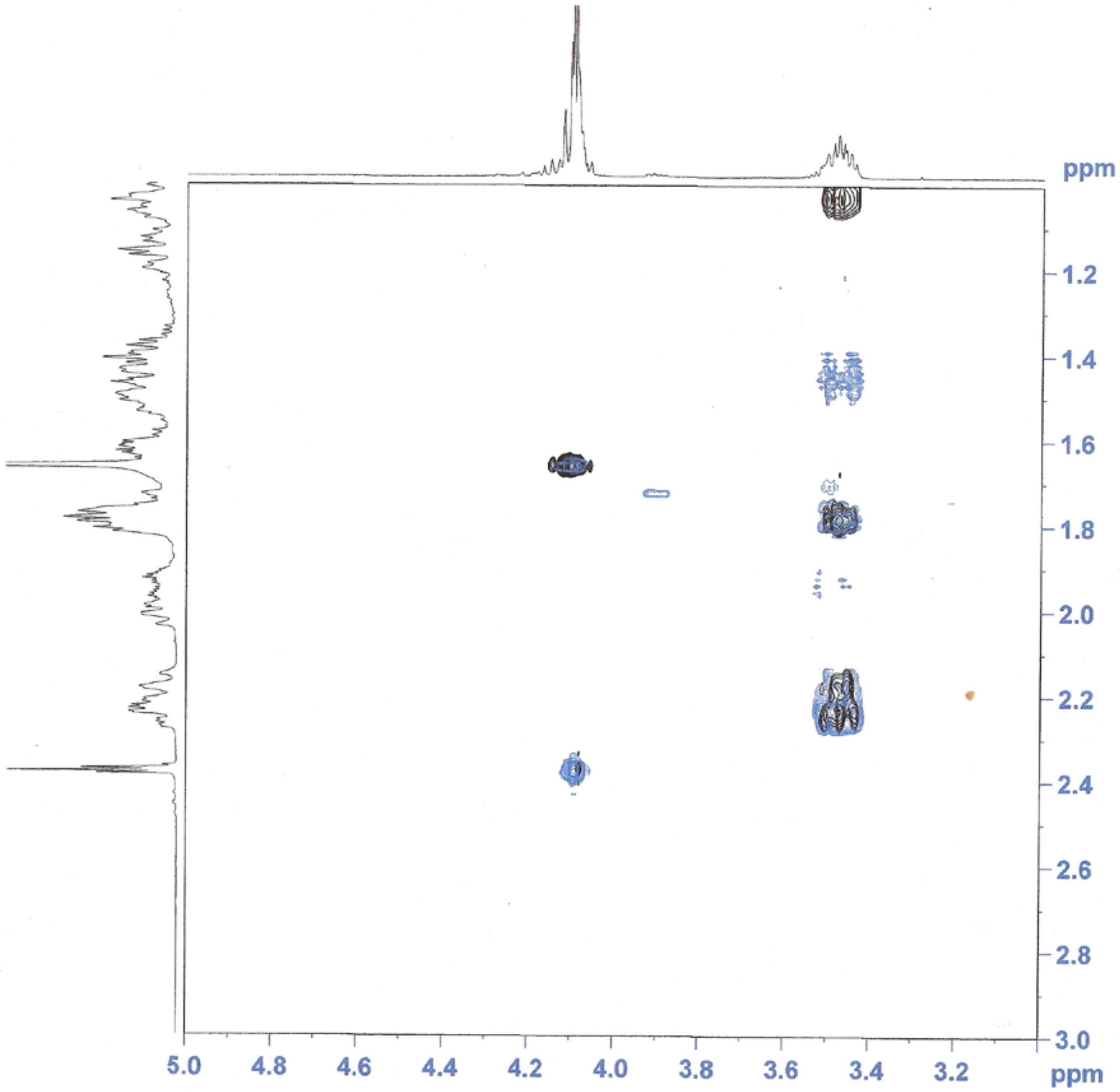
ppm
1.2
1.4
1.6
1.8
2.0
2.2
2.4
2.6
2.8
3.0
5.0
4.8
4.6
4.4
4.2
4.0
3.8
3.6
3.4
3.2
ppm

^{1}H NMR 24-norchol-5,20(22-cis)-dien-3β-ol-23-propargyl ether (**3**)

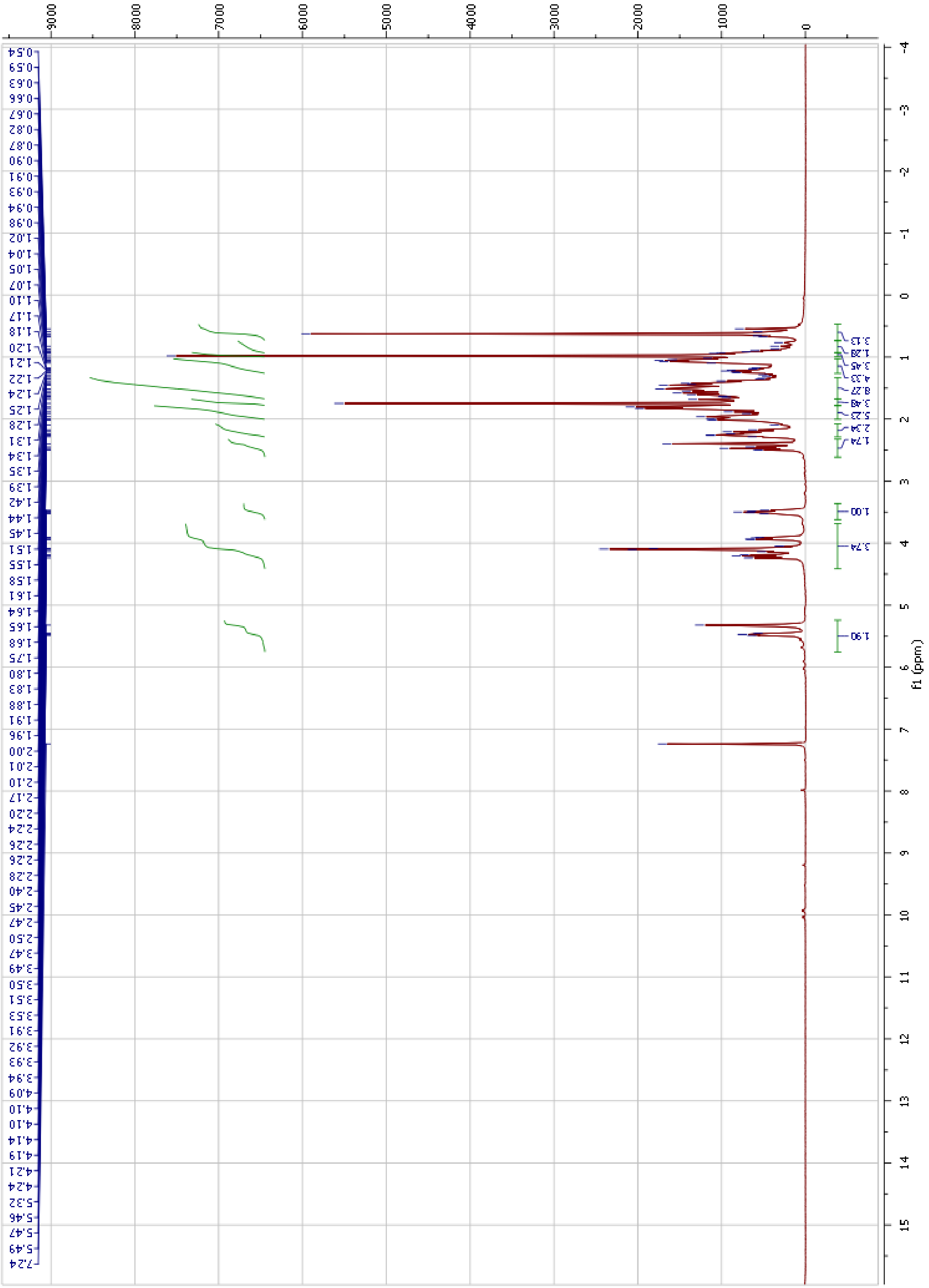

^{13}C NMR 24-norchol-5,20(22-cis)-dien-3β-ol-23-propargyl ether (**3**)

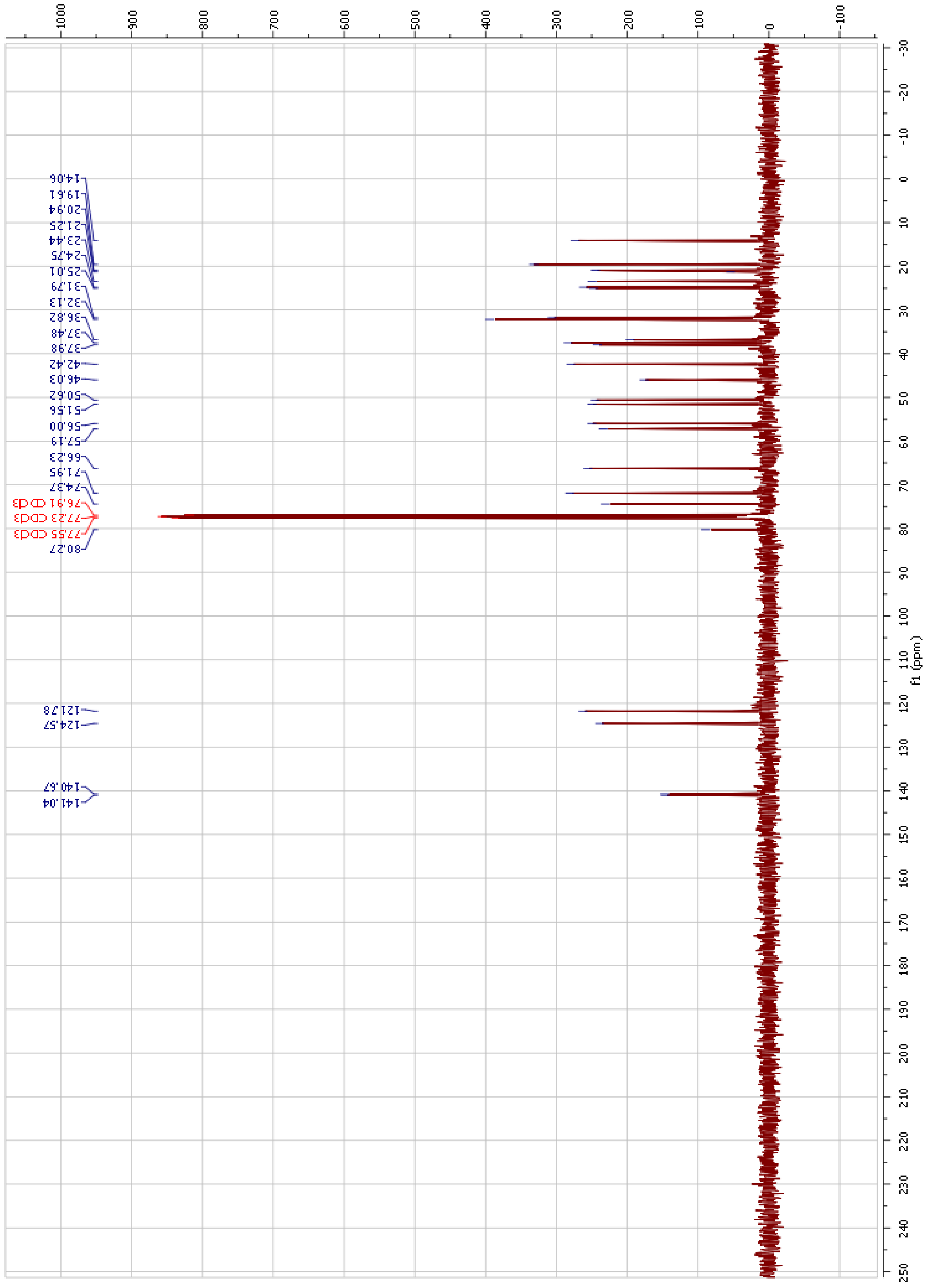

NOE NMR (black) and COSY (blue) 24-norchol-5,20(22-cis)-dien-3β-ol-23-propargyl ether (**3**)

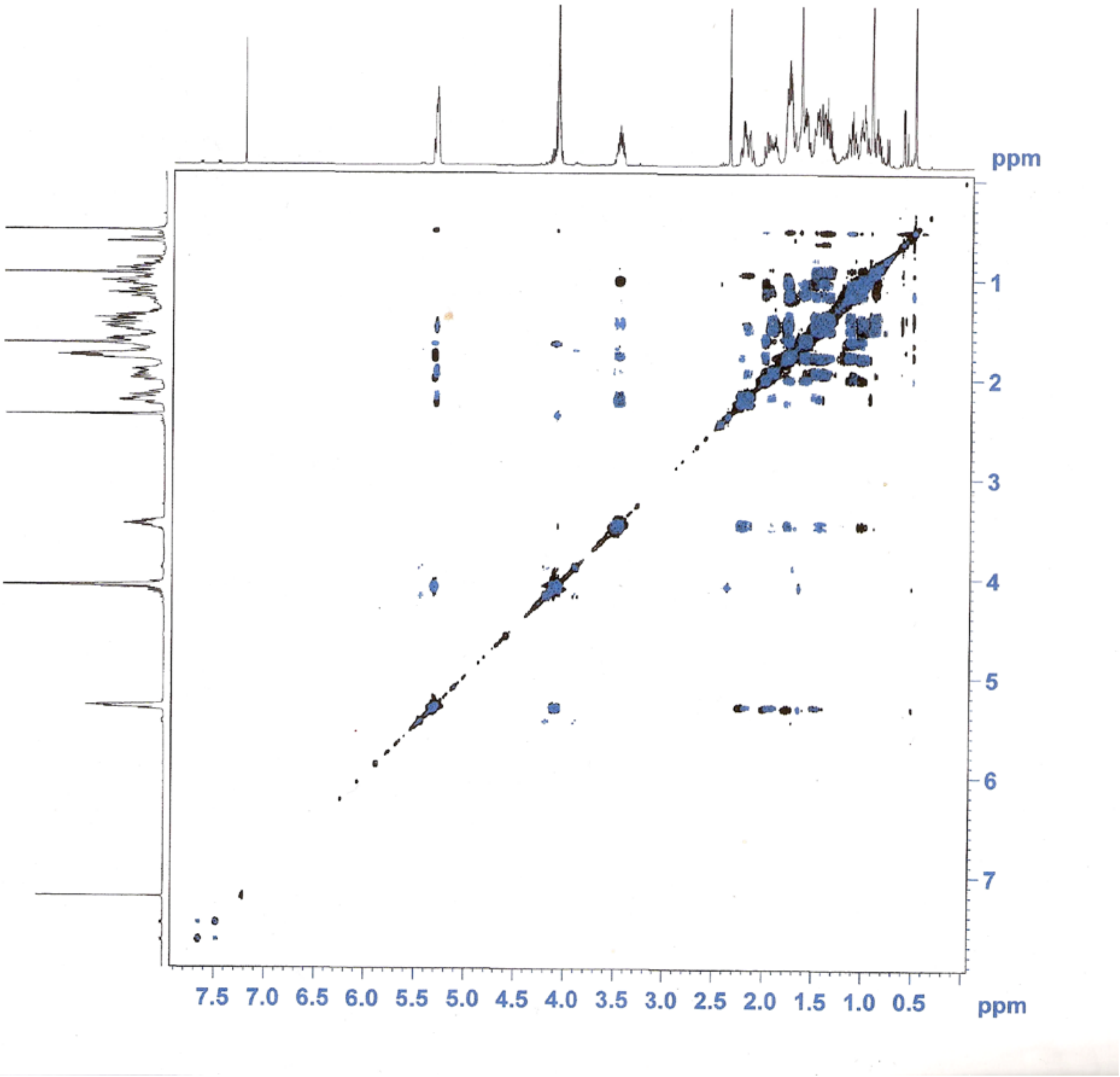

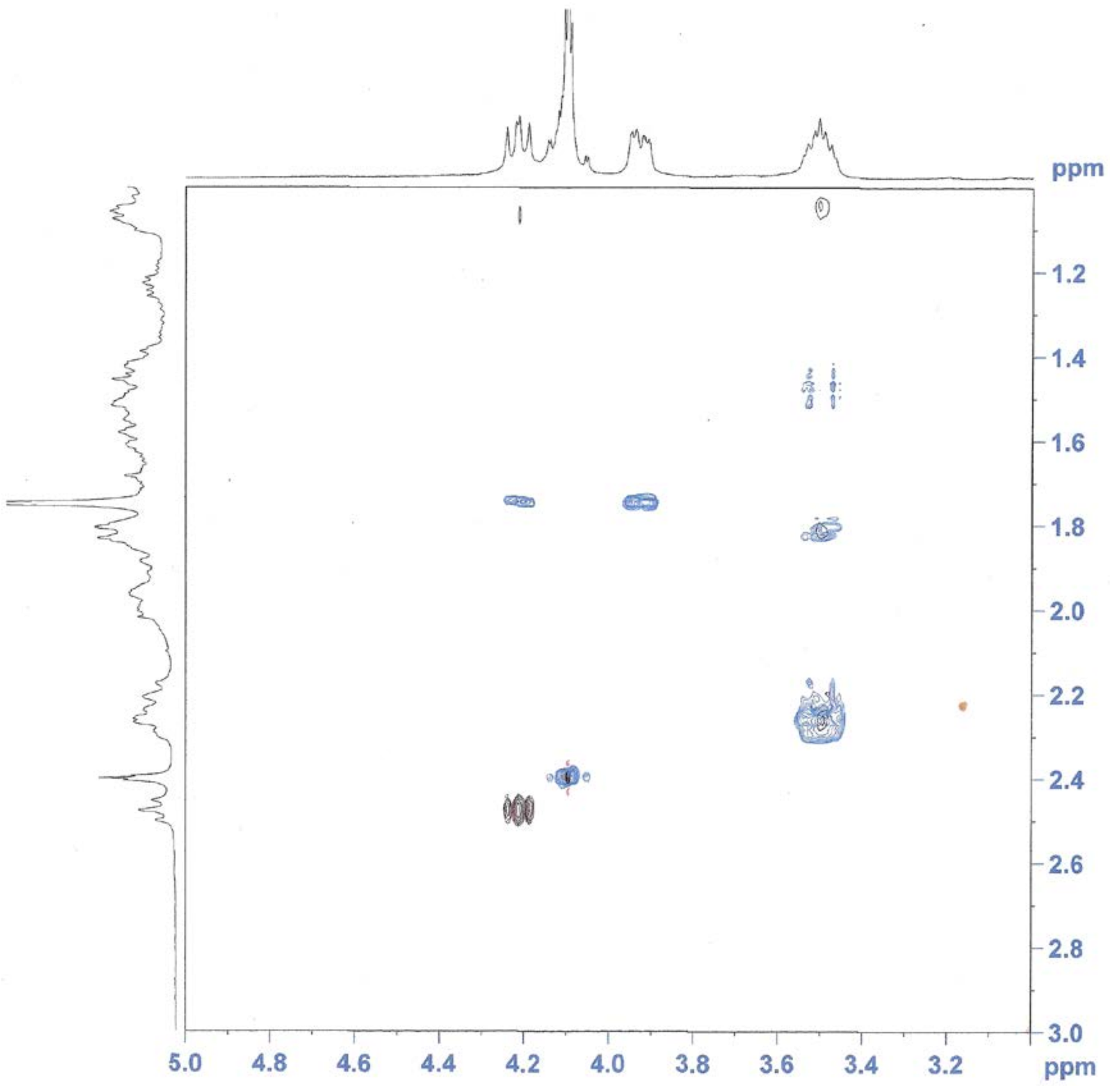
ppm
1.2
1.4
1.6
1.8
2.0
2.2
2.4
2.6
2.8
3.0
5.0
4.8
4.6
4.4
4.2
4.0
3.8
3.6
3.4
3.2
ppm

^{1}H NMR Chol-5-en-3β-ol-23-propargyl ether (**4**)

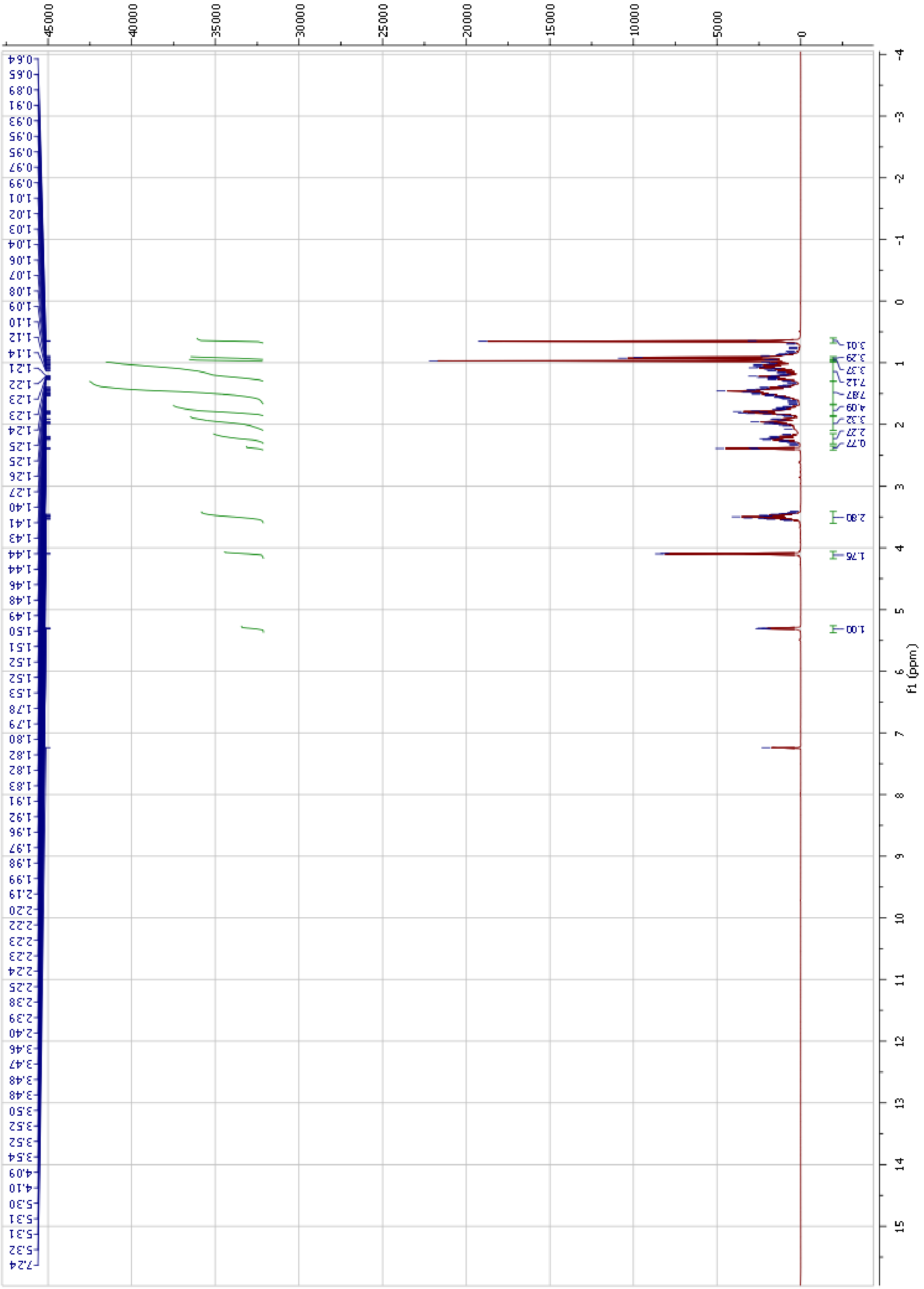

^{13}C NMR Chol-5-en-3β-ol-23-propargyl ether (**4**)

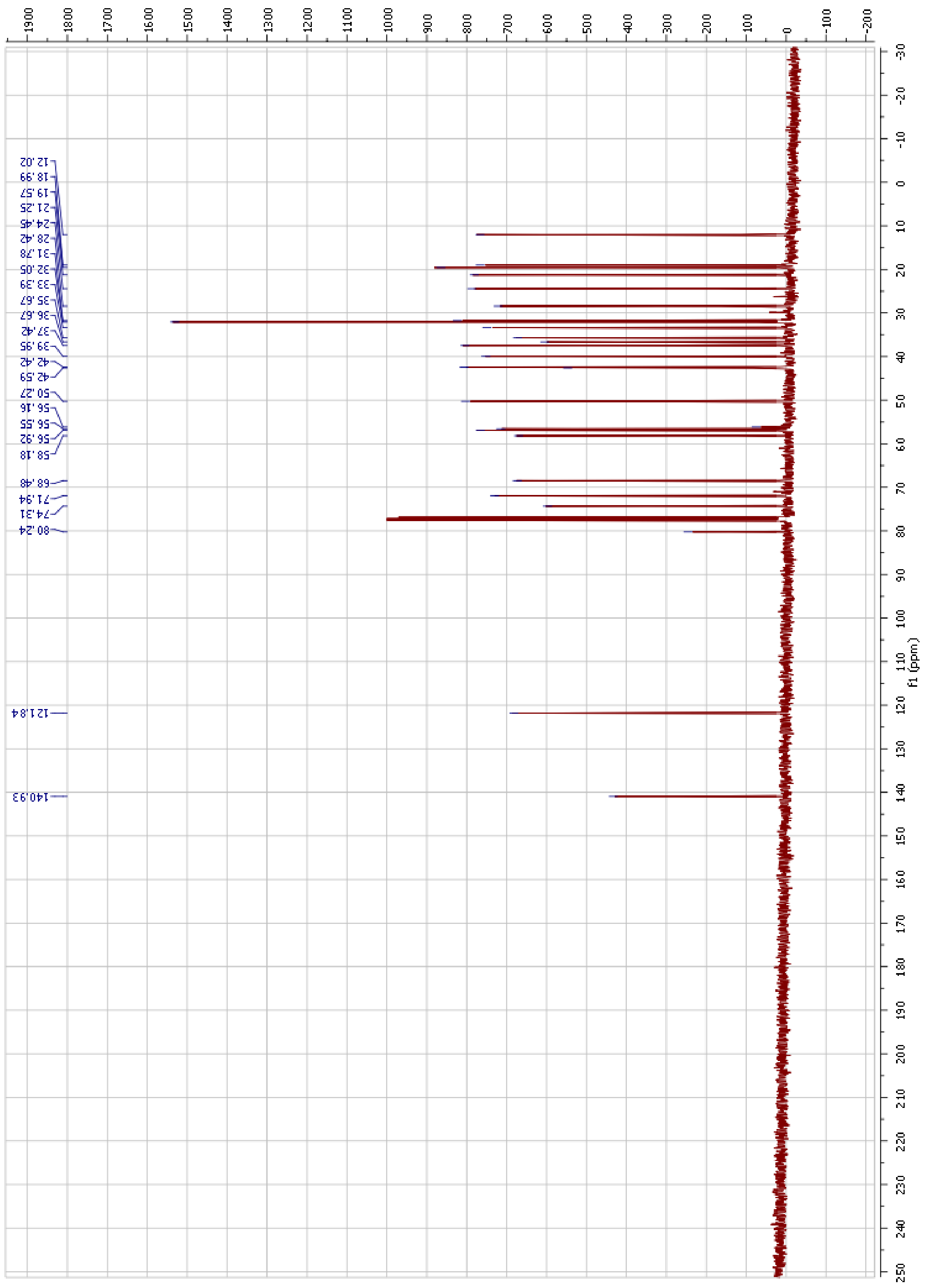

^{1}H NMR 3β-hydroxychol-5-en-23-yne (**5**).

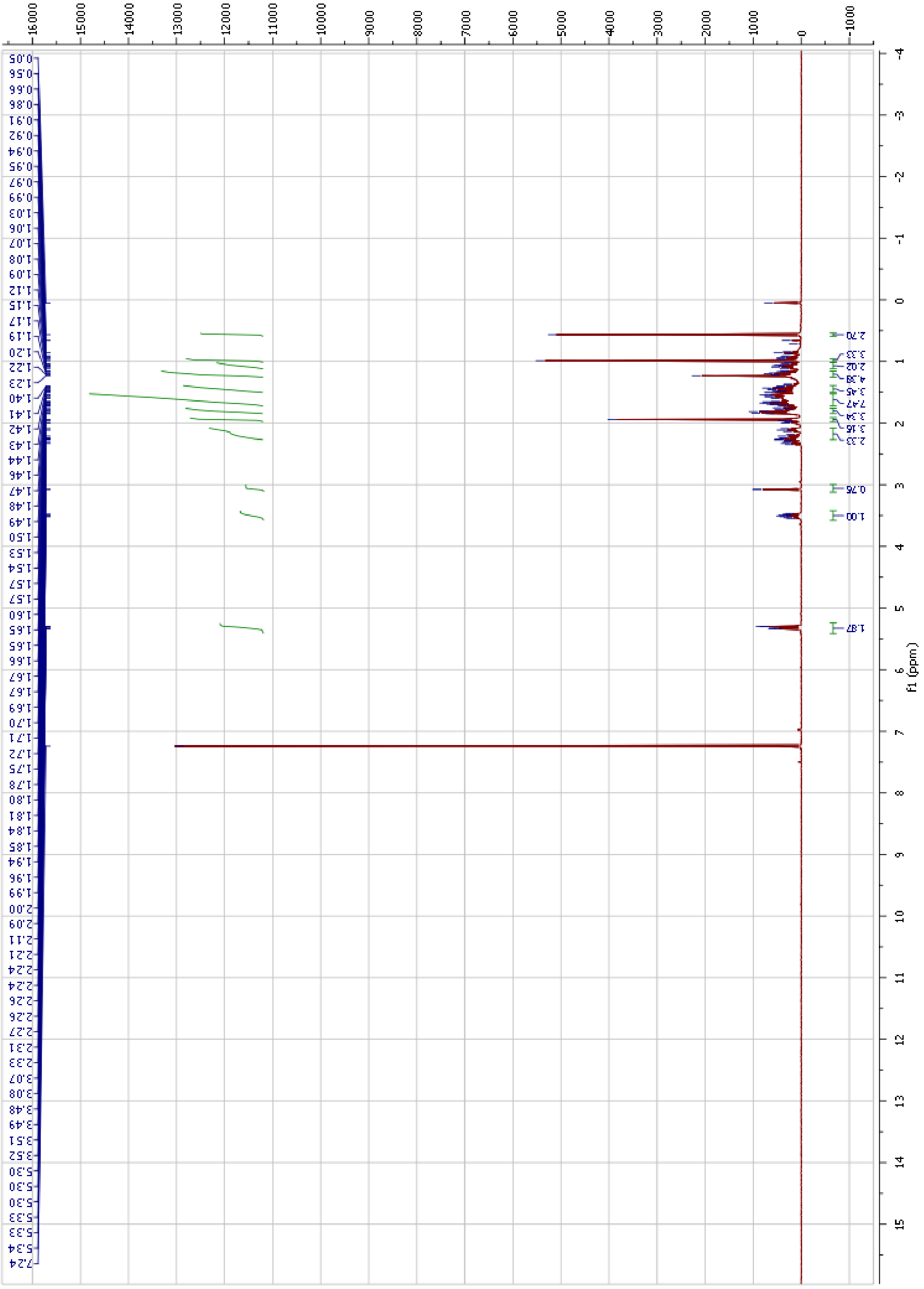

^{13}C NMR 3β-hydroxychol-5-en-23-yne (**5**).

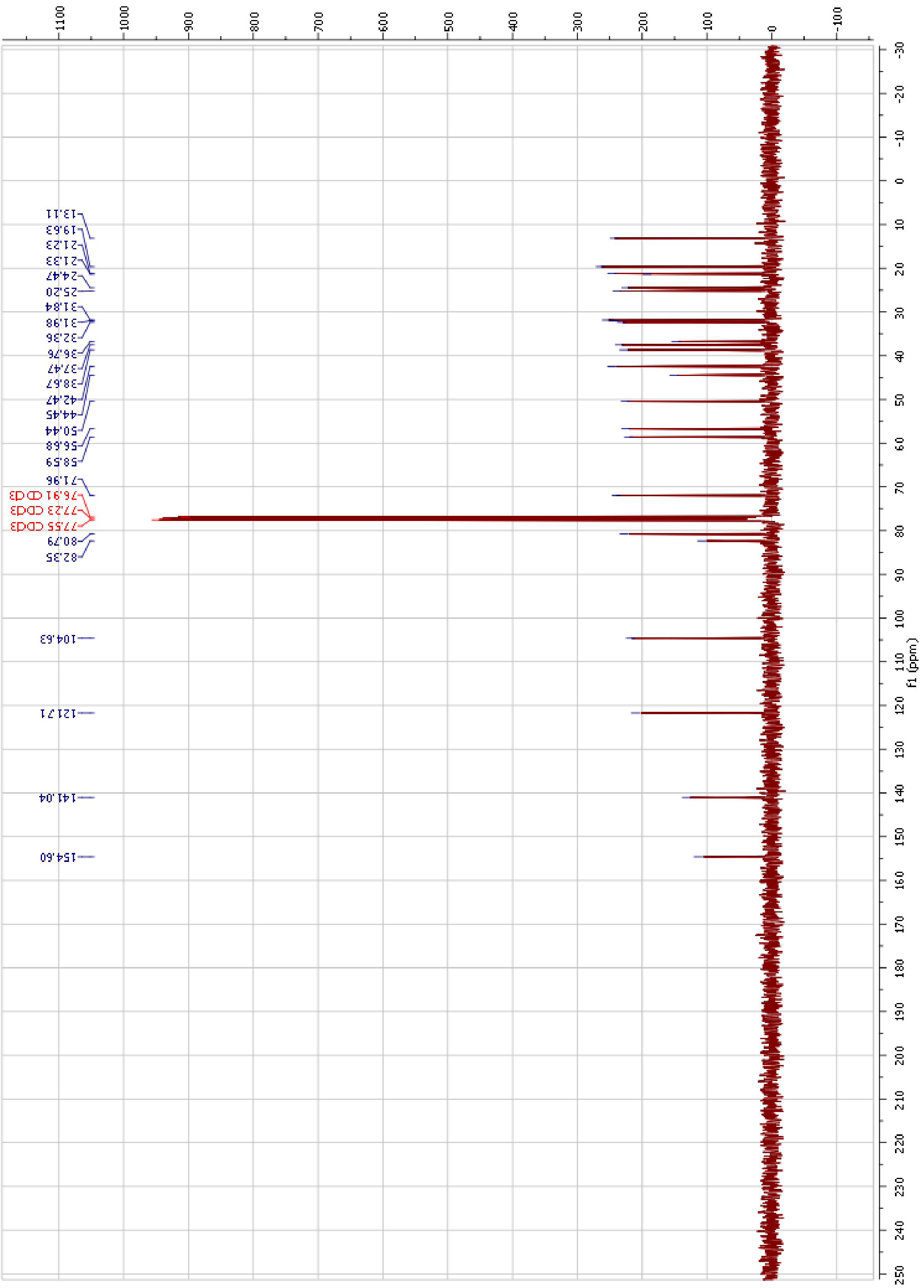

^{1}H NMR 3β-hydroxychol-5-en-24-yne (**6**).

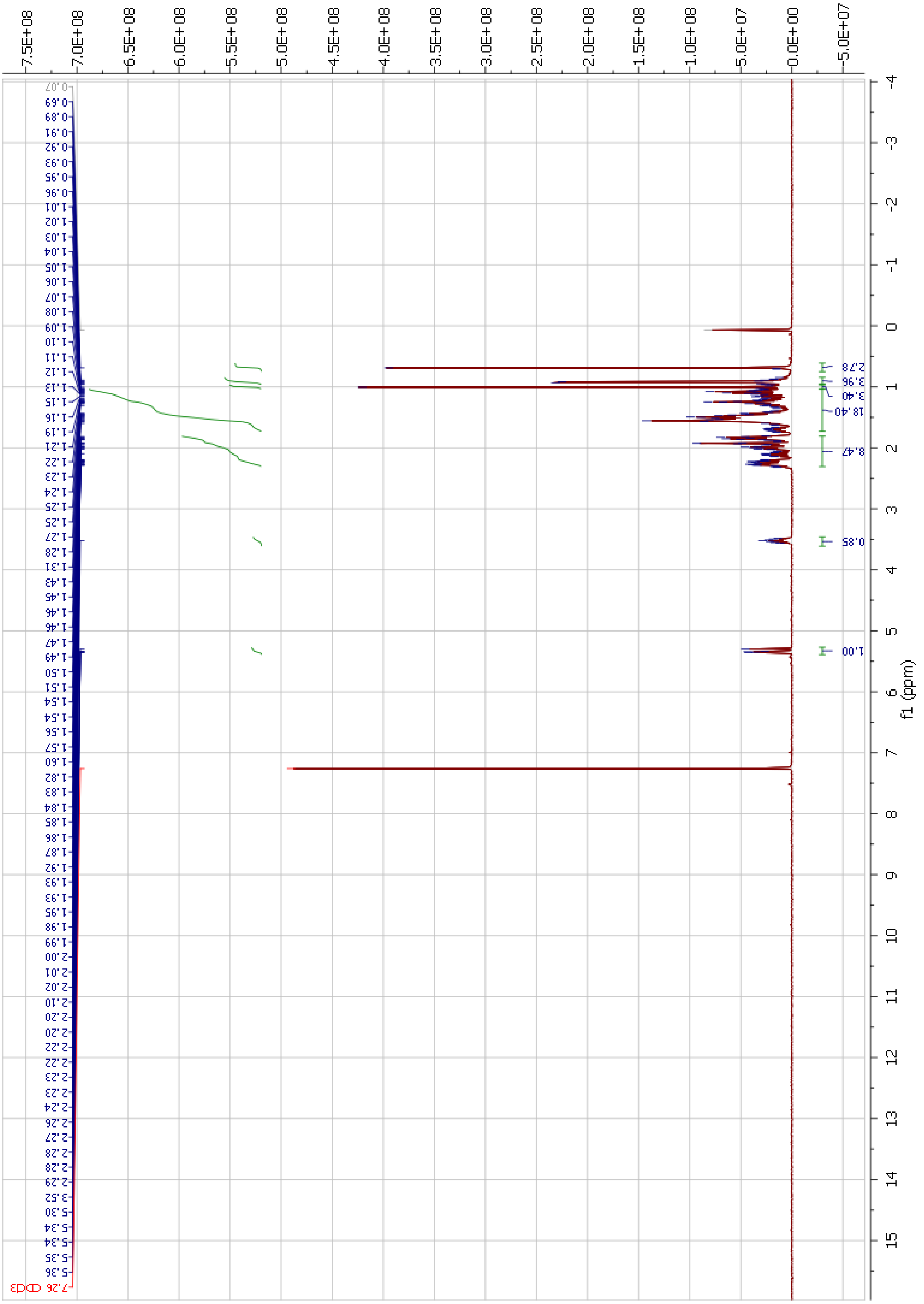

^{13}C NMR 3β-hydroxychol-5-en-24-yne (**6**).

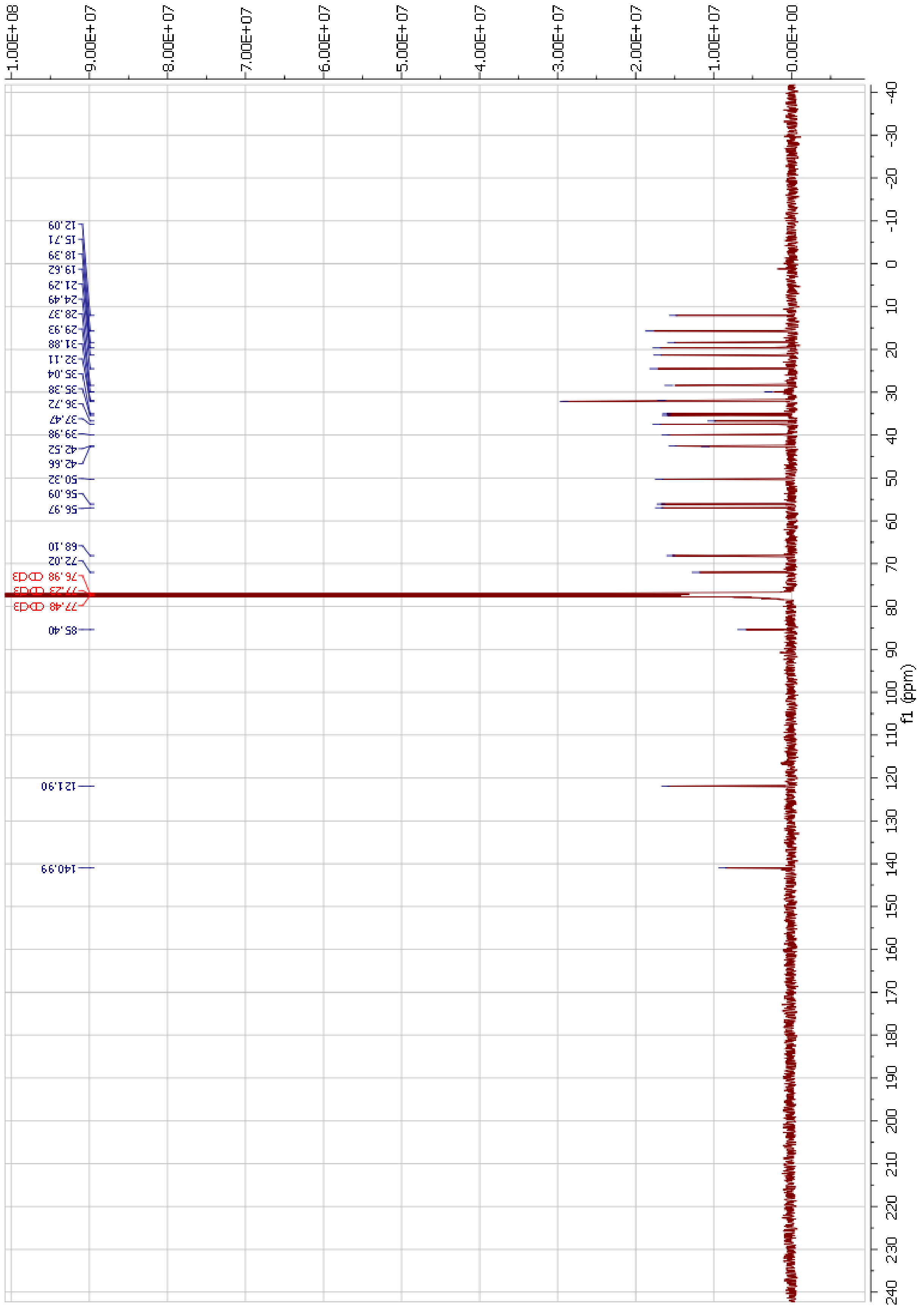

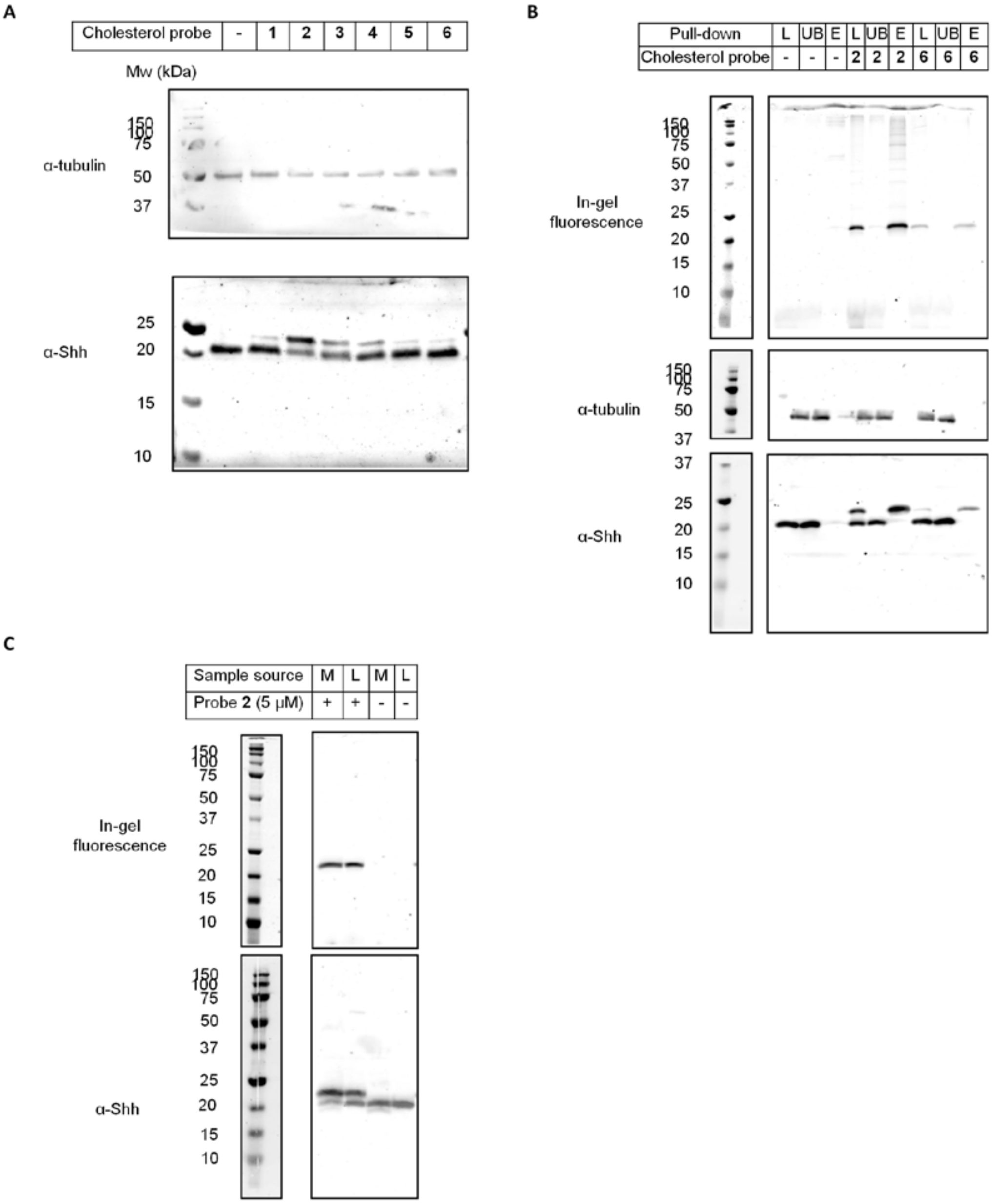

Fig. S6 Full gel images. (A) Immunoblot image for Figure 3A; (B) In-gel fluorescence image and immunoblot for Figure 3B; (C) In-gel fluorescence image and immunoblot for Figure 5B.

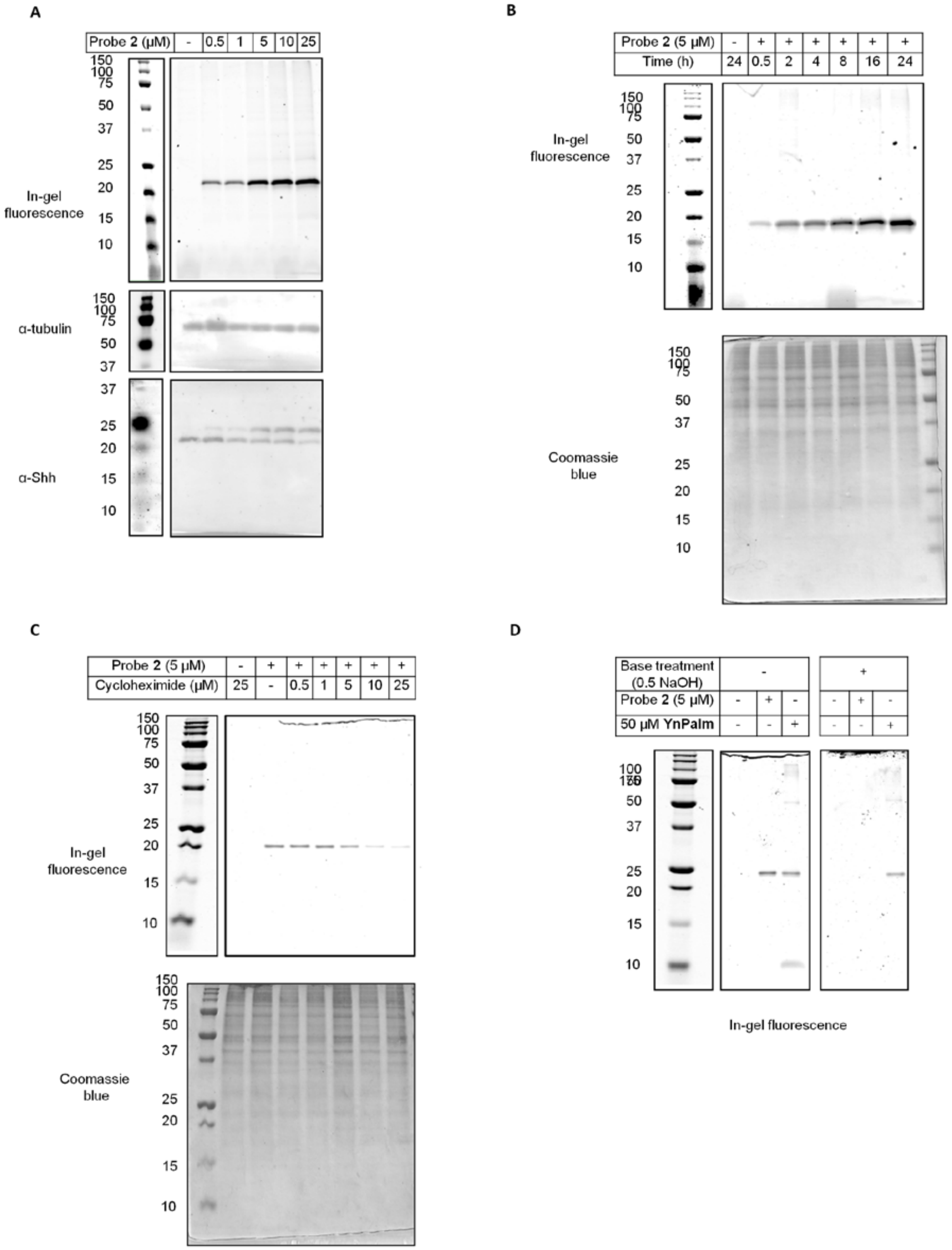

Fig. S7 Full gel images. (A) In-gel fluorescence image and immunoblot for Figure 4A; (B) In-gel fluorescence image and Coomassie blue staining of gel for Figure 4B; (C) In-gel fluorescence image and Coomassie blue staining of gel for Figure 4C; (B) In-gel fluorescence image of gels for Figure 4D.

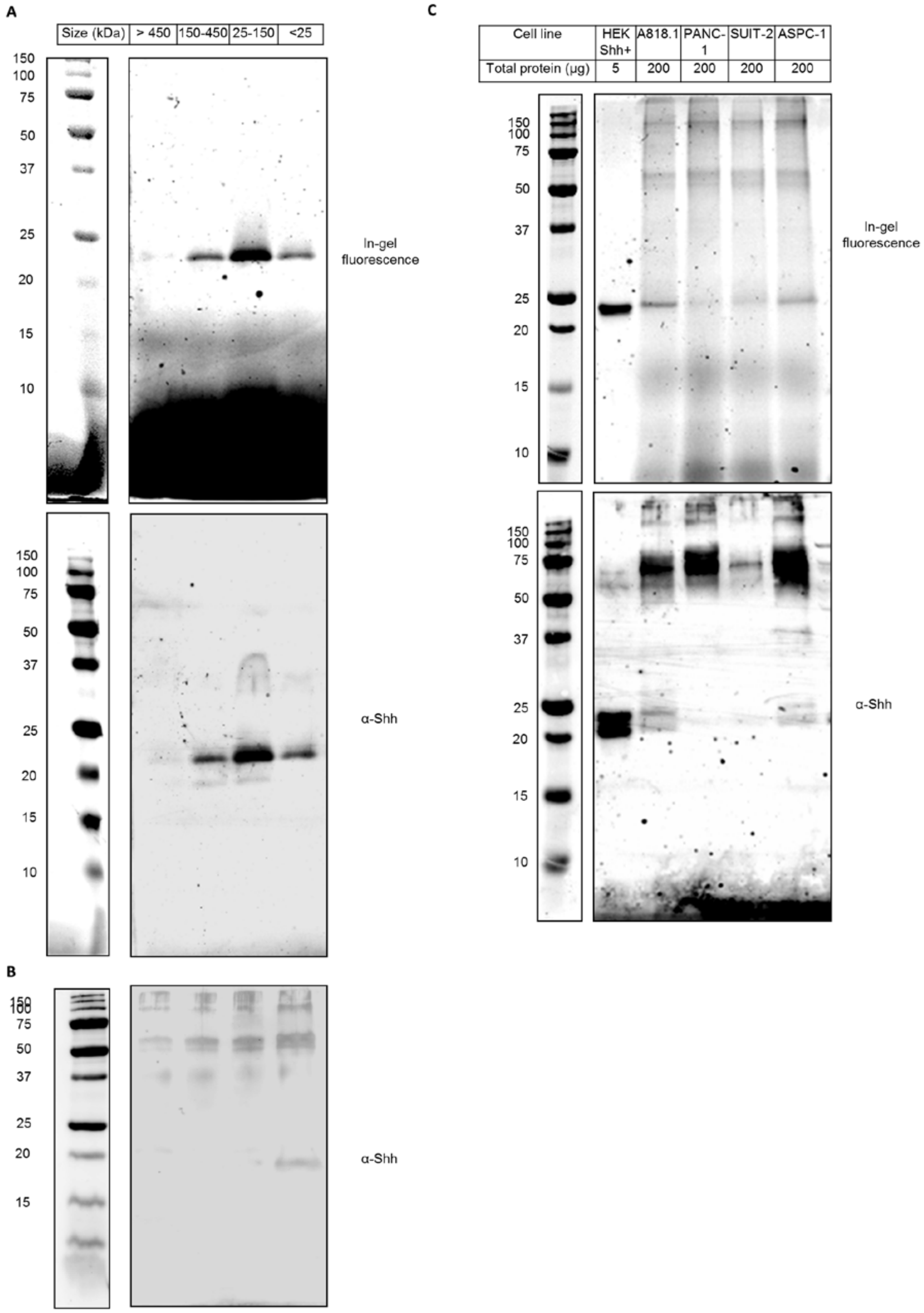

Fig. S8 Full gel images. (A) In-gel fluorescence image and immunoblot for Figure 6A; (B) In-gel fluorescence image and immunoblot for Figure 6B; (C) In-gel fluorescence image and immunoblot for Figure 7A.

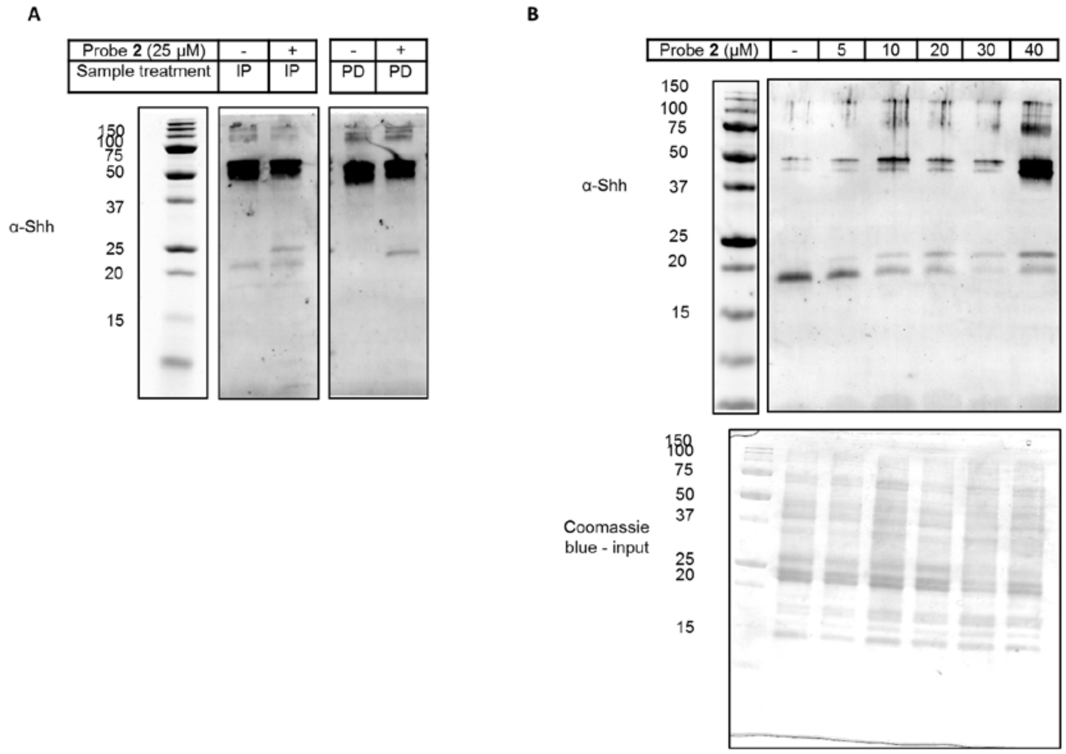

Fig. S9 Full gel images. (A) Immunoblot for Figure 9A; (B) Immunoblot and Coomassie blue staining of gel for Figure 9B.

References:

1. S. T. Laughlin, J. M. Baskin, S. L. Amacher and C. R. Bertozzi, *Science*, 2008, 320, 664-667.
2. V. Link, A. Shevchenko and C.-P. Heisenberg, *BMC Developmental Biology*, 2006, 6, 1.
3. F. I. Hinz, D. C. Dieterich, D. A. Tirrell and E. M. Schuman, *ACS Chemical Neuroscience*, 2011, 3, 40-49.
4. J. Vestergaard, M. W. Pedersen, N. Pedersen, C. Ensinger, Z. Tümer, N. Tommerup, H. S. Poulsen and L. A. Larsen, *Lung Cancer*, 2006, 52, 281-290.
5. J. Rappsilber, M. Mann and Y. Ishihama, *Nat. Protoc.*, 2007, 2, 1896-1906.
6. J. Cox and M. Mann, *Nat. Biotechnol.*, 2008, 26, 1367-1372.
7. K. R. Bharucha, G. C. Buckley, C. K. Cross, L. J. Rubin and P. Zlegler, *Can. J. Chem.*, 1956, 34, 982-990.
8. M. D. Rahman, H. M. Seidel and R. A. Pascal, *J. Lipid Res.*, 1988, 29, 1543-1548.
9. C. D. Schteingart and A. F. Hofmann, *J. Lipid Res.*, 1988, 29, 1387-1395.
10. V. Sepe, R. Ummarino, M. V. D'Auria, B. Renga, S. Fiorucci and A. Zampella, *Eur. J. Org. Chem.*, 2012, 2012, 5187-5194.
